# Prenatal and childhood exposure to common plasticizers in relation to emotional and behavioral development through adolescence

**DOI:** 10.1016/j.scitotenv.2026.181869

**Published:** 2026-05-12

**Authors:** Lilly Meerts, Hanan El Marroun, Yuchan Mou, Mengling Liu, Leonardo Trasande, Henning Tiemeier, Kurunthachalam Kannan, Vincent W.V. Jaddoe, Tonya White, Akhgar Ghassabian

**Affiliations:** a Department of Child and Adolescent Psychiatry, Erasmus MC – Sophia Children’s Hospital, Rotterdam, the Netherlands; b Department of Psychology, Education and Child Studies, Erasmus School of Social and Behavioral Sciences, Rotterdam, the Netherlands; c Department of Population Health, NYU Grossman School of Medicine, New York, NY, USA; d Department of Pediatrics, New York University Grossman School of Medicine, New York, NY, USA; e Department of Social and Behavioral Sciences, Harvard T.H. Chan School of Public Health, Boston, MA, USA; f Department of Environmental Health Sciences, College of Integrated Health Sciences, State University of New York at Albany, New York, NY, USA; g Wadsworth Center, New York State Department of Health, Albany, New York, NY, USA; h The Generation R Study Group, Erasmus MC, University Medical Centre, Rotterdam, the Netherlands; i Department of Pediatrics, Erasmus MC, University Medical Centre, Rotterdam, the Netherlands; j Section on Social and Cognitive Developmental Neuroscience, National Institute of Mental Health, Bethesda, MD, USA; k Department of Epidemiology, Harvard T.H. Chan School of Public Health, Boston, MA, USA

**Keywords:** Endocrine disruptors, Environmental exposure, Phthalates, Bisphenol, Child mental health, Behavior, Emotions

## Abstract

**Background:**

Individuals are ubiquitously exposed to bisphenols and phthalates, common plasticizers that may affect neurodevelopment. We examined associations of prenatal and childhood bisphenol and phthalate exposure with internalizing and externalizing problems from early childhood through adolescence.

**Methods:**

Within the Generation R study, prenatal urinary concentrations of bisphenol A (BPA) and phthalate metabolites were assessed in early, mid- and late pregnancy and in childhood at age 6 years. Pregnancy levels were averaged and used in analyses. Internalizing and externalizing problems were reported by parents at child age 3, 6, 10 and 14 years and by children at ages 10 and 14 years. Mother-child dyads with at least one prenatal exposure measure and one internalizing or externalizing problem score during follow-up were included (*n* = 1361). Among children with childhood exposure measures, *n* = 651 had at least one internalizing or externalizing problem score. Associations were examined using linear mixed models. Mixture analysis was performed for self-reported scores at age 14 with G-computation.

**Findings:**

Associations between phthalate/bisphenol levels and child outcomes were largely null. Except, prenatal mono-n-butylphthalate and mono-(2-ethyl-5-hydroxyhexyl)phthalate were associated with higher self-reported internalizing problem scores in girls (B_mBP_ = 0.16, 95%CI: 0.03, 0.28 and B_mEHHP_ = 0.11, 95%CI: 0.03,0.19, respectively). Prenatal mono-isobutyl phthalate, mono-(2-ethyl-5-carboxypentyl)phthalate and mono-(4-carboxymethyl-hexyl)phthalate were associated with higher self-reported externalizing problem scores in boys (B_mIBP_ = 0.12, 95%CI: 0.03, 0.20, B_mECPP_ = 0.17, 95%CI: 0.07, 0.28 and B_mCMHP_ = 0.19, 95%CI: 0.08, 0.30, respectively). Childhood mono-methyl was associated with lower parent-reported externalizing problem scores in boys (B_mMP_ = −0.12, 95%CI: −0.22, −0.03). Childhood mono-Benzyl phthalate was associated with higher self-reported externalizing problem scores in boys (B_mBzP_ = 0.12, 95%CI: 0.04, 0.20). No associations with BPA were found. G-computation showed positive, but non-significant, associations for the same metabolites as in single chemical analyses.

**Conclusions:**

Associations of BPA and phthalate exposure with internalizing and externalizing problem scores in adolescents were largely null, associations with childhood phthalate exposure were less consistent and harder to interpret.

## Graphical Abstract

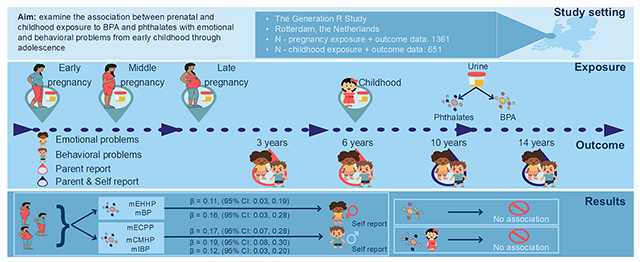

## 1. Introduction

An increasing body of research suggests that environmental exposures, particularly endocrine disrupting chemicals (EDCs), such as bisphenols and phthalates, influence neurodevelopment (Yesildemir and Celik, 2024). Bisphenols and phthalates are synthetic chemicals widely used in consumer products, such as food and beverage packaging, personal care products, flooring material and clothing (Sathyanarayana, 2008; Michalowicz, 2014). The widespread use of bisphenols and phthalates leads to ubiquitous exposure in the general population (Philips et al., 2018; Casas et al., 2013). Sensitive periods of development, such as the prenatal period, may be particularly vulnerable to the impact of bisphenols and phthalates on neurodevelopment (Sarigiannis et al., 2021; Oskar et al., 2024; Bellinger, 2013), due to rapid development of several organs and critical processes occurring during this period. On the other hand, childhood may represent another important window for neurodevelopmental impact of bisphenols and phthalates, given the continued maturation of the brain including processes such as synaptic pruning and myelination, combined with sustained exposure to these chemicals (National Research Council Committee on the Health Risks of P, 2008; Dualde et al., 2021; Taki and Kawashima, 2012). Experimental data indicate that thyroid and sex hormone disruption, oxidative stress, epigenetic modifications, and direct neurotoxicity leading to brain structural and functional alterations are potential underlying mechanisms for neurodevelopmental influences of exposure to bisphenols and phthalates (Jankowska et al., 2021; Minatoya and Kishi, 2021). These mechanistic findings in animal studies provide biological plausibility for the potential impact of bisphenol and phthalate exposure on neurodevelopment.

Empirical research suggests that childhood emotional and behavioral problems can be classified into internalizing problems (i.e. more inwardly directed), such as anxiety, shyness and depression, and externalizing problems (more outwardly directed), such as aggression, rule-breaking behavior and attention problems (Achenbach et al., 2016; Ogundele, 2018). These clusters of internalizing and externalizing symptoms in childhood are shown to be strong predictors of adult mental health (Caspi et al., 1996; Schlack et al., 2021). Several studies have examined the extent to which prenatal and childhood phthalate exposure is associated with internalizing and externalizing problems in childhood. However, findings have been inconclusive (Minatoya and Kishi, 2021; Radke et al., 2020). For example, one study reported that higher prenatal urinary concentrations of mono-(3-carboxypropyl) phthalate (mCPP) were associated with higher internalizing and externalizing problem scores in children aged 2–8 years, whereas other phthalates were not related to internalizing and externalizing problems. In the same study, higher childhood mono-benzyl phthalate (mBzP), mono-carboxy-isononyl phthalate (mCNP), mono-carboxy-isooctyl phthalate (mCOP), mCPP and mono-ethyl phthalate (mEP) concentrations were associated with higher externalizing problem scores (Li et al., 2020a). Additionally, a recent study by Oh et al. found that mono-Benzyl phthalate (mBzP) and Mono-hexyl phthalate (MHxP) specifically were associated to higher externalizing problems, but they found null-associations for internalizing problems all measured in children aged 1.5–5 years (Oh et al., 2025). Hyland et al. found largely null associations, except that higher prenatal exposure to low-molecular weight (LMW) phthalates, that are mostly used in cosmetic and personal care products, was associated with higher self-reported anxiety, attention problems and hyperactivity in children aged 16 (Hyland et al., 2019). In another study, higher prenatal exposure to di(2-ethylhexyl) phthalate metabolites (DEHP) was associated with lower rather than higher internalizing problem scores in children aged 2–9 years (Messerlian et al., 2017). Metabolites of DEHP are so-called legacy phthalates that are currently regulated in baby products in several countries, including European Unions and the United States.

In addition to phthalates, bisphenols—particularly bisphenol A (BPA)—are another class of endocrine-disrupting chemicals of concern for child neurodevelopment with largely inconsistent findings (Inadera, 2015). For example, a Japanese cohort study found no relationship between prenatal BPA exposure and externalizing problems (Minatoya et al., 2018). However, in another study prenatal, but not childhood, BPA exposure was associated with internalizing problems in children aged 2–5 years (Grohs et al., 2019). Li et al. reported higher internalizing and externalizing problems in children aged 2–4 years, who had higher prenatal BPA exposure (Li et al., 2020b). In contrast, Evans et al. reported no association between prenatal BPA exposure and externalizing or internalizing outcome in children aged 6–10 (Evans et al., 2014). In another study, childhood exposure to BPA associated with higher total problem scores in children aged 9–11 years (Perez-Lobato et al., 2016). These mixed findings highlight the need for further research.

Previous studies explored sex differences in the associations between bisphenols, phthalates, and neurodevelopmental outcome (Jankowska et al., 2021; Minatoya and Kishi, 2021), given the potential of these chemicals to modulate the endocrine system, such as sex hormone pathways (Ullah et al., 2022). For phthalates, some studies reported associations primarily in boys. Prenatal mono-butyl phthalate (mBP) concentrations were associated with higher internalizing problem scores in boys aged 3–5 years (Philippat et al., 2017). Similarly, maternal pregnancy urinary concentrations of mono-isobutyl phthalate (mIBP) and mBzP were associated with higher anxiety scores in boys aged 7 years. In the same study, a mixture analysis of phthalates showed no associations in either sex (Daniel et al., 2020). Regarding BPA, one study reported that the associations of prenatal BPA exposure with internalizing and externalizing problems were more strongly associated in boys than girls (Li et al., 2020b). In contrast, another study found that prenatal BPA exposure was associated with higher externalizing problem scores in girls but not boys aged 1–8 years (Braun et al., 2017). Stacey et al. reported that prenatal BPA exposure was associated with higher externalizing problem scores in girls, while childhood exposure was associated with more externalizing problems in boys (Stacy et al., 2017). These studies had small to moderate sample sizes with typically 350–400 participants. The inability to replicate findings on sex differences underscores the need for larger studies that are sufficiently powered to examine associations in boys and girls separately.

While, in general, these findings suggest that prenatal and childhood exposure to specific phthalates and prenatal BPA exposure may be associated with internalizing and externalizing problems, inconsistencies remain across studies. Moreover, most of these studies had follow up through early and mid-childhood and long-term follow-up periods beyond the age of 10 years are lacking. In addition, only a few studies examined both prenatal and childhood exposure to bisphenols and phthalates (Li et al., 2020a; Grohs et al., 2019; Stacy et al., 2017).

Against this background, we examined the longitudinal association of prenatal and childhood exposure to bisphenols and phthalates with internalizing and externalizing problems from early childhood through adolescence. We further formally examined sex differences in these associations by first including interaction terms in the single chemical models, followed by stratified analyses. We hypothesized that prenatal exposure to bisphenols and phthalates would be associated with higher internalizing and externalizing problem scores through adolescence. Additionally, we hypothesized that associations would differ between boys and girls, although the directions of expected differences were uncertain due to inconsistencies in previous studies.

## 2. Methods

### 2.1. Participants

We used data from the Generation R Study, a population-based prospective cohort focused on early-life environmental and genetic influences on health and development in Rotterdam, The Netherlands (White et al., 2018; Kooijman et al., 2016). In brief, pregnant women residing in Rotterdam, who had an estimated delivery date between April 2002 and January 2006 were invited to participate in the study. A total of 9778 participants enrolled in the study during pregnancy. Urine sample collection started in February 2004. From 4918 pregnant participants enrolled between 2004 and 2006 (the period in which urine collection was implemented), 2089 participants provided three spot urine samples at <18 weeks, 18–25 weeks and >25 weeks of gestation, which were used for measurements of phthalate metabolites and bisphenols (Fig. S1). We included mother-child dyads with urinary measures of phthalates and bisphenols in pregnancy and one or more measures of internalizing and externalizing outcomes from age 3 to 14 years (*n* = 1361). A subset of children provided a spot urine sample at age 6 years (*n* = 775 children). Among these children with childhood urinary measures of phthalates and bisphenols, *n* = 651 children had one or more measures of internalizing and externalizing outcomes during follow-up and were included for analyses of childhood exposure. Among all included mother-child dyads there were 258 instances during which, at a specific age, only an internalizing or externalizing score, but not the other, could be derived because of missing item-level data.

Approval for the study was granted by the Medical Ethics Committee of the Erasmus Medical Center, Rotterdam. Adult participants provided written informed consent and children older than 12 years provided assent for participation in the study (Kooijman et al., 2016).

### 2.2. Bisphenol and phthalate exposure assessment

Bisphenols and phthalate metabolite levels were quantified in spot urine samples of pregnant participants three times: <18 weeks (i.e. early pregnancy), 18–25 weeks (i.e. mid pregnancy), and >25 weeks of gestation (i.e. late pregnancy) and children at age 6 years at the Wadsworth Center, New York State Department of Health, Albany, New York. Sample collection, storage, and analytical procedures of urine samples have been discussed in detail elsewhere (Philips et al., 2018; Kruithof et al., 2014).

Phthalate metabolites were quantified by enzymatic deconjugation of glucuronidated phthalate monoesters, solid-phase extraction and high performance liquid chromatography, coupled with electrospray ionization-tandem mass spectrometry (HPLC-ESI-MS/MS) (Asimakopoulos et al., 2016). This method detected eighteen phthalate metabolites with each a limit of detection (LOD) of 0.008–0.3 ng/mL, which is considered the lower limit for reliable detection. The metabolites include: mono-methyl phthalate (mMP), mEP, mBP, mIBP, mono-(2-ethyl-5-carboxypentyl) phthalate (mECPP), mono-(2-ethyl-5-hydroxyhexyl)phthalate (mEHHP), mono-(2-ethyl-5-oxohexyl) phthalate (mEOHP), mono-[(2-carboxymethyl)hexyl]phthalate (mCMHP), mCPP, mBzP, mono-hexylphthalate (mHxP), mono-2-heptylphthalate (mHpP), monooctylphthalate (mOP), monoisononylphthalate (mINP), mono-hydroxy-isodecyl phthalate (mIDP), mono-cyclohexyl phthalate (mCHP), and mono(7-carboxyheptyl) phthalate (mCHpP). We also quantified phthalic acid (PA) as a proxy of total phthalate exposure (Philips et al., 2018).

Bisphenol concentrations were quantified using HPLC-ESI-MS/MS. This method enabled detection of eight bisphenols with LODs of 0.03–0.79 ng/mL. The bisphenol biomarkers analyzed include: BPA, Bisphenol S (BPS), Bisphenol Z (BPZ), Bisphenol B (BPB), Bisphenol F (BPF), Bisphenol AP (BPAP), Bisphenol AF (BPAF) and Bisphenol P (BPP). Urinary creatinine concentration was quantified using HPLC-ESI-MS/MS and had an LOD of 0.30 ng/mL. Creatinine levels were used to account for urinary dilution. All bisphenol and phthalate metabolite concentrations were divided by the individual’s creatinine concentration at the timepoint of measurement and standardized for the population median creatinine concentration (Kuiper et al., 2022).

Urinary biomarkers were included for further statistical analysis if >70% of metabolite concentrations exceeded the LOD. Levels below the LOD were substituted by LOD/√2, a method demonstrated to minimize bias for small proportions of values below the LOD (Seok et al., 2023).

### 2.3. Child internalizing and externalizing problems

Child emotional and behavioral problems were repeatedly assessed at ages 3, 6, 10 and 14 years with the Achenbach System of Empirically Based Assessment (ASEBA) forms. The ASEBA forms are validated tools widely used for evaluating emotional and behavioral functioning across the life span with good reliability (Achenbach and Rescorla, 2001a, 2001b; Achenbach, 2000). Main care givers (mainly mothers) completed the Child Behavioral Checklist (CBCL) and the children completed the Brief Problem Monitor Youth (BPM–Y) and Youth Self-Report (YSR) (White et al., 2018; Tiemeier et al., 2012; Blok et al., 2022). For ages 3 and 6 years, the CBCL preschool version was used (Achenbach, 2000). At age 6 years assessments, the CBCL preschool version was used for all children participating in the respective examination to ensure uniformity, as half of the children were younger than 6 years. For ages 10 and 14 years, the CBCL school-age version was administered (Achenbach and Rescorla, 2001a). At age 10 years the BPM–Y, a 19-item short form derived from the YSR, was completed rather than the full YSR, to limit participant burden at this relatively young age (White et al., 2018; Achenbach et al., 2011). However, the BPM and YSR are highly correlated and the BPM has been validated (Rognstad et al., 2022; Piper et al., 2014). At age 14 the YSR was completed (Achenbach, 1991). For all instruments, items were answered with a three-point Likert scale (0 = not true, 1 = somewhat true, 2 = very true). For the CBCL, BPM-Y and YSR, internalizing problem scores, reflecting emotional problems, and externalizing problem scores, reflecting behavioral problems, were calculated (see Table S1 for an overview of instrument characteristics). Both scores demonstrate good reliability in assessing internalizing and externalizing problems in Dutch populations (Koot et al., 1997). All internalizing and externalizing problem scores were standardized for harmonization across ages, raters and for comparability across scales. *Z*-scores were calculated for each instrument and time of assessment (e.g. for each age group) separately, by subtracting the mean from the raw score and then dividing the score by the standard deviation (SD). This approach was preferred over standardized T-scores, as the latter are based on age- and instrument-specific norms, limiting comparability across repeated assessments. Applying this to every datapoint yields a standard normal distribution of internalizing and externalizing problem scores per age with mean = 0 and SD = 1. *Z*-scores for internalizing and externalizing problem scores were used in all models. Theoretically, z-scores are unbounded, but in our sample observed z-scores ranged from approximately −1.3 to 6.8 across scales and instruments (Table S1).

### 2.4. Covariates

Information on covariates was collected through questionnaires and/or objective assessments. Sociodemographic factors, such as parental age, highest education level finished, country of origin, parity, marital status and family income were retrieved from questionnaires (Kooijman et al., 2016). Maternal smoking in pregnancy and alcohol consumption in pregnancy were also obtained from questionnaires during pregnancy (Kooijman et al., 2016). Maternal and child dialkylphosphate (DAP) concentrations, non-specific metabolites of organophosphate (OP) pesticides were measured in spot urine samples with gas chromatography coupled with tandem mass spectrometry (Kruithof et al., 2014; van den Dries et al., 2018). Dialkylphosphates, which are metabolites of organophosphate pesticides were included in our analysis as a covariate, since dietary intake is a shared source of exposure to OP pesticides, bisphenols and phthalates, and OP pesticides are shown to have neurotoxic effects (Gunderson, 1995; Muncke, 2009).

Pre-pregnancy maternal body mass index (BMI) was calculated using pre-pregnancy weight and height measurements at the first prenatal visit. Further, we included children’s age at emotional and behavioral assessments and BMI at age 6 years, which was calculated based on weight and height measured during in-person visits. Gestational age at urine sampling was calculated based on ultrasound scans during in-person routine visits (Gaillard et al., 2014). Other child variables, such as gestational age at birth, birth weight and sex assigned at birth were obtained from medical records.

### 2.5. Statistical analysis

Descriptive statistics, i.e., medians and interquartile ranges (IQRs) for continuous covariates and percentages, for categorical covariates, were used to describe the study population. We also reported median (IQR), percentage below LOD, and the intra class correlations (ICCs) between the three pregnancy measures of chemicals. These ICCs were calculated to assess reliability of the measurements. The ICC was calculated using the single measurement and absolute agreement two-way mixed-effects model (Koo and Li, 2016). Correlations among bisphenols and phthalate metabolites and between measurements at different time points were assessed with Spearman correlation matrices and correlation plots.

Bisphenol and phthalate concentrations were log2 transformed to improve the distribution of residuals in linear models. Average concentrations for each chemical were calculated across pregnancy to provide a more stable approximation of prenatal bisphenol and phthalate exposure. This approach accounts for the short half-lives of the chemicals, which can otherwise lead to high within-individual variability over time (Perrier et al., 2016; Spaan et al., 2015). These average concentrations were subsequently used in models investigating prenatal exposure.

For single pollutant analyses, linear mixed models (LMM) were used to examine associations of urinary bisphenol and phthalate metabolite concentrations with repeated internalizing and externalizing problem scores through age 14 years, with parental and self-reported scores analyzed separately. The sample size differed across analyses due to availability of exposure and outcome data (Table S2). With LMMs, correlations of within-child measurements across timepoints are taken into account by subject-specific random intercepts, modelling the average developmental pattern. Random slopes for age were not included, as these models did not converge and thus we focused on random intercepts for simplicity and interpretability, though we acknowledge heterogeneity in behavioral trajectories. This model type does not rely on the independence of data assumption and is specifically designed to be applied in hierarchical data structures, such as with repeated measurements (Yang et al., 2014). Further, LMMs flexibly accommodate unbalanced data structures, such as with different numbers of observations across participants. Model estimations appear to be robust even when model assumptions are violated (Schielzeth et al., 2020). Among the limitations, LMMs could produce biased estimates when the random effects structure is misclassified. This statistical approach optimally utilizes repeated outcome measurements, which thereby conceptually aligned best with our primarily longitudinal research question, rather than, for instance, single time point analyses. In addition, this approach allows for the investigation of interactions with age, handles missing outcome data more efficiently and reduces potential multiple testing error. Prenatal and age 6 years exposures were examined in separate models, each adjusting for exposure in the other window. In models with chemical exposure at age 6 years, only internalizing and externalizing scores measured after exposure measurement were included, namely 10 years and 14 years.

Confounders were selected based on Directed Acyclic Graphs (DAGs; Fig. S2) informed by prior literature on bisphenol and phthalate exposure and behavioral outcome or based on biologically plausible relationships between confounders, exposures, and outcomes (Philips et al., 2018). Models were adjusted for sociodemographic factors, including maternal age (years), highest education level finished (low/medium or high), country of origin (Netherlands, Morocco, Suriname, Turkey, other European and other), parity (0 or 1+) marital status (married/partner or single) and family income (<€1200/month, €1200–€2000/month or >€2000/month), as well as history of maternal smoking (no smoking, smoked until pregnancy recognized and continued smoking during pregnancy) and alcohol consumption (no alcohol consumption, alcohol consumption until pregnancy recognized, continued occasionally and continued frequently) in pregnancy. Additionally, maternal and child urinary concentrations DAPs were included as confounders. DAP molar sums were calculated using concentrations of metabolites with >70% above the LOD (Table S4). Collinearity of DAP molar sums with bisphenol and phthalate metabolite levels was assessed and no substantial multicollinearity was detected. Models also included child sex (boy/girl) and age at assessment. We did not adjust prenatal exposure models for gestational age at birth, birth weight, and maternal psychopathology, as they may lie on the causal pathway (Jacobson et al., 2022). We hypothesize these covariates likely to be mediators and adjusting for those would introduce over-adjustment bias. Maternal psychopathology was also not included as a covariate in childhood exposure models. Prenatal chemical exposure models were additionally adjusted for pre-pregnancy BMI, gestational age at exposure measurement, and child BPA and PA concentrations (precision variables). Childhood 6 years chemical exposure models were additionally adjusted for child BMI (precision variable), gestational age at birth and prenatal BPA and PA concentrations (confounders). Interaction with sex was tested and all models were run for boys and girls separately to assess sex as a potential effect modifier.

Missing data on confounders were imputed using the Multivariate Imputation by Chained Equations (MICE) algorithm (van Buuren and Groothuis-Oudshoorn, 2011; Team RC, 2024), since our data suggested that missingness was associated with observed covariates, supporting a Missing At Random mechanism. Within MICE, the Random Forest method was used in R (R package). A total of 30 imputed datasets were generated, each based on 30 iterations and 100 trees. Both bisphenol and phthalate concentrations, as well as CBCL, BPM and YSR scores were included as predictors, in addition to covariates, gestational age and birth weight.

Inverse probability weights were applied to LMMs to account for potential selection bias due to loss to follow up. Weights were calculated using the full Generation R cohort and with child sex, gestational age at birth, maternal age, maternal pre-pregnancy BMI, parity, country of origin, marital status, maternal smoking and drinking in pregnancy as predictors. Multiple testing correction was performed by estimating the effective number of independent tests based on the correlation matrices of exposures as proposed by Li and Ji (Li et al., 2012; Li and Ji, 2005). In this approach, eigenvalue decomposition of exposure correlation matrices is used to estimate the number of independent comparisons. The number of independent comparisons was estimated for prenatal and childhood exposure separately. With the number of estimated independent comparisons, the nominal significance level (α = 0.05) is divided, resulting in an adjusted significance level of α = 0.010 for models with prenatal exposure and α = 0.011 for models with childhood exposure. The adjusted significance levels were applied across all analyzed exposures, outcomes and for main models as well as for sex stratified models.

Several additional analyses were performed to test robustness of findings. First, we used quantile g-computation to estimate the joint association of the prenatal exposure mixture with self-reported internalizing and externalizing problem scores at age 14 years. Outcomes at this age were selected, because of our interest in whether prenatal exposures have lasting effects through adolescence. G-computation regression models were adjusted for aforementioned covariates. Second, DAP molar sums were excluded as a covariate for all models to test the impact of adjusting for OP pesticide exposure. Third, LMM with pregnancy exposures were run, adjusting for all covariates but without adjusting for childhood BPA and PA concentrations, to investigate the effect of adjustment for exposure in another time window. Additionally, LMM with childhood exposures were run, adjusting for all covariates but without adjusting for pregnancy BPA and PA concentrations. Fourth, the effect of time on the exposure-outcome relationship was investigated by including an interaction term with children’s age at assessment. Fifth, non-linearity of the exposure-outcome relationship was investigated by comparing outcomes of children with exposures in the highest tertile to children with exposures in the lowest tertile. Sixth, we refit all the models on complete cases only to evaluate the robustness of the results under varying assumptions regarding missing data.

All analyses were carried out using R (version 4.4.1) (Team RC, 2024).

## 3. Results

### 3.1. Sample characteristics

Table 1 presents sample characteristics. Women from the included mother/child dyads (*n* = 1361) had a median age of 31 years at intake and median BMI of 22.7 kg/m^2^. Most women were nulliparous (61.2%), of Dutch origin (54.9%), finished higher professional education or had a university degree (51.6%), were married or cohabiting (89.1%) and never smoked (75.3%) during pregnancy. Half of the children in the sample were boys (50.6%). Children were born at median gestational age of 40 weeks and had a median age of 5.8 years at the time of spot urine sample collection. Missingness in sample characteristics ranged from 0.5% to 14.3% per variable (See Footnote, Table 1).

Median prenatal bisphenol concentrations ranged from 0.1 to 1.7 ng/mL across early, mid and late pregnancy (Table 2). Median bisphenol concentrations in children at age 6 years ranged from 0.2 ng/mL to 0.8 ng/mL (Table 2). Only BPA had less than 30% of concentrations below the LOD; therefore, other bisphenols were excluded from further analysis. The ICC for BPA across pregnancy was 0.01 (Table S3), the proportion of missingness was lowest parity for and highest for household income.

Median prenatal phthalate metabolite concentrations ranged from 0.1 to 153.5 ng/mL across early, mid and late pregnancy (Table 2). Median phthalate metabolite concentrations in children at age 6 years ranged from 0.1 ng/mL to 15.4 ng/mL. Metabolites mINP, mCHP, mOP, mIDP, mHxP, mHpP and mCHpP had more than 30% of concentrations below the LOD, meaning that more than 30% of measurements were too low to be reliably quantified, and were excluded from further analysis. The ICC for phthalate exposure across pregnancy ranged from 0.00 to 0.18 (Table S3).

Correlations between prenatal BPA and phthalate metabolite concentrations ranged from −0.03 between mEOHP in mid pregnancy and mBzP in late pregnancy to 0.97 between mEOHP and mEHHP both measured in early pregnancy (Fig. S3A). The correlations between childhood BPA and phthalate metabolite concentrations ranged from 0.12 between BPA and mBzP, to 0.98 between mEOHP and mEHHP (Fig. S3B). Correlations between pregnancy average BPA and phthalate metabolites and childhood BPA and phthalate metabolites were weak and ranged from −0.04 between pregnancy mBP and childhood BPA, to a maximum of 0.25 between pregnancy mEP and childhood mEP (Fig. S3C).

Median concentrations for DAP metabolites during pregnancy varied from 0 ng/mL (for DEDTP) to 12.2 ng/mL (for DMP). In children, median DAP concentrations varied from 0 ng/mL to 6.7 ng/mL (Table S4).

Median parent-rated internalizing problem scores ranged from 3 to 4 and median externalizing problem scores ranged from 2 to 8 (Table 3). For Self-reports, median internalizing problem scores ranged from 1 to 9 and median externalizing problem scores ranged from 1 to 7. Correlations between parent reported and child self-reported outcome scores were in the range of 0.20–0.68 (Fig. S4). At age 10 years correlations between CBCL and YSR scores were 0.30 for internalizing problems and 0.40 for externalizing problems respectively. Correlations between parent reported and child self-reported outcome scores at age 14 years were 0.49 for both internalizing problems and externalizing problems. Correlations between self-reported scores at 10 and 14 years were 0.38 for internalizing problems and 0.32 for externalizing problems.

### 3.2. Average prenatal exposure to bisphenols and phthalates

#### 3.2.1. Internalizing problems

**Fig. 1.**
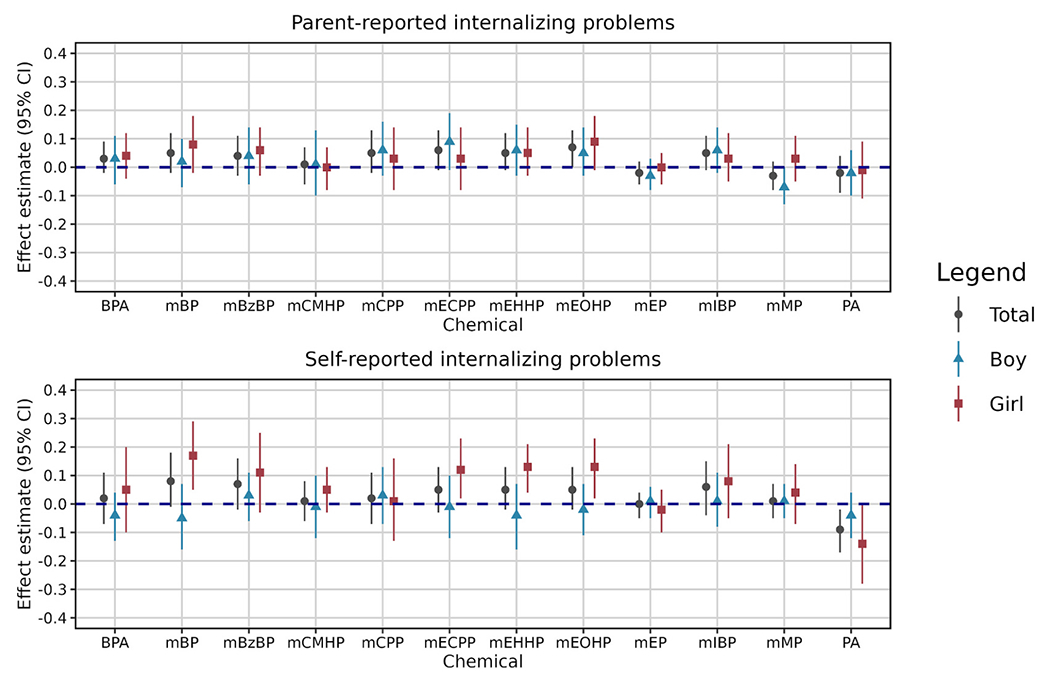
Associations of prenatal BPA and phthalate exposure with internalizing problem score through age 14 years. *Figure note*: Models were adjusted for maternal age, pre-pregnancy body mass index, parity, country of origin, maternal educational levels, marital status, maternal smoking and alcohol drinking habits and urinary concentrations of organophosphate pesticides during pregnancy, as well as gestational age at the time of chemical measurements, child sex (only in models with all children) and child age at outcome measurement. Effect estimates are reported per log-2 unit increase in creatinine adjusted concentrations of BPA and phthalate metabolites averaged across three measurements in pregnancy. For parent-reports, internalizing problem scores were obtained at ages 3, 6, 10, and 14 years, and for child self-reports internalizing problem scores were obtained at ages 10 and 14 years. All internalizing problem scores were standardized. Vertical lines represent 95% Confidence Intervals. Based on models including interactions between sex and single chemicals, significant interaction terms (*p* < 0.10) were observed for: Parent-reported internalizing problems – mMP; Self-reported internalizing problems – mBP, mECPP, mEHHP, mEOHP. Based on models including interactions between age and single chemicals, significant interaction terms (p < 0.10) were observed for: Parent-reported internalizing problems – none; Self-reported internalizing problems – none.

Overall, we did not find associations between BPA or phthalate exposure and child internalizing problems as reported by parents in LMM (Fig. 1). Two associations emerged, but did not survive multiple testing correction. A 2-fold increase in creatinine-adjusted mIBP concentrations were associated with higher internalizing problems in boys only (B = 0.08 per 2-fold increase in creatinine-adjusted prenatal mIBP, 95% CI: 0.00, 0.15). Higher prenatal mEOHP concentration was associated with higher internalizing problem scores in all children (B = 0.06, 95% CI: 0.00, 0.13).

For self-reported internalizing problems, each 2-fold increase in creatinine-adjusted prenatal mBP (B = 0.16, 95% CI: 0.03, 0.28), mECPP (B = 0.13, 95% CI: 0.03, 0.23), mEHHP (B = 0.11, 95% CI: 0.03, 0.19) and mEOHP (B = 0.11, 95% CI: 0.01, 0.21) concentrations were associated with higher internalizing problems only in girls (Fig. 1). The associations of mBP and mEHHP concentrations with internalizing problems in girls survived multiple testing correction. In contrast, maternal pregnancy urinary PA was associated with lower internalizing problem scores (B = −0.09, 95% CI: −0.17, −0.02). This association did not survive multiple testing correction. No other prenatal phthalate metabolites or BPA were associated with self-reported internalizing problem scores.

#### 3.2.2. Externalizing problems

**Fig. 2.**
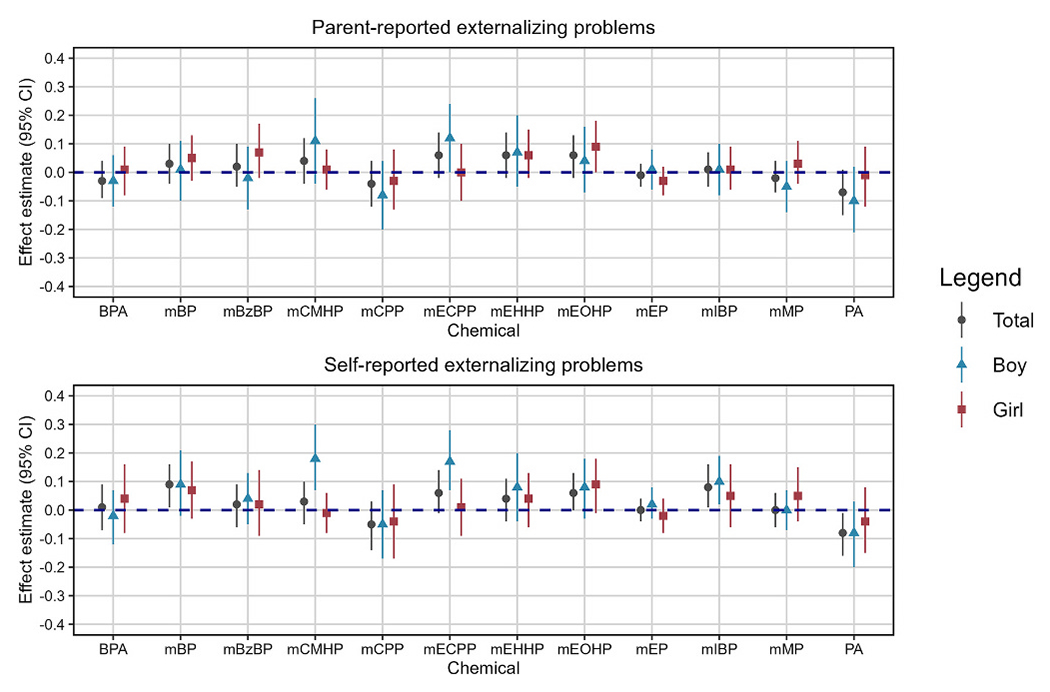
Associations of prenatal BPA and phthalate exposure with externalizing problem score through age 14 years. *Figure note*: Models were adjusted for maternal age, pre-pregnancy body mass index, parity, country of origin, maternal educational levels, marital status, maternal smoking and alcohol drinking habits and urinary concentrations of organophosphate pesticides during pregnancy, as well as gestational age at the time of chemical measurements, child sex (only in models with all children) and child age at outcome measurement. Effect estimates are reported per log-2 unit increase in creatinine adjusted concentrations of BPA and phthalate metabolites averaged across three measurements in pregnancy. For parent-reports, externalizing problem scores were obtained at ages 3, 6, 10, and 14 years, and for child self-reports externalizing problem scores were obtained at ages 10 and 14 years. All internalizing problem scores were standardized. Vertical lines represent 95% Confidence Intervals. Based on models including interactions between sex and single chemicals, significant interaction terms (p < 0.10) were observed for: Parent-reported externalizing problems – none; Self-reported externalizing problems – mECPP, mCMHP.. Based on models including interactions between age and single chemicals, significant interaction terms (p < 0.10) were observed for: Parent-reported externalizing problems – mBP; Self-reported externalizing problems – BPA.

Fig. 2 represents the associations of prenatal BPA and phthalate metabolites with child externalizing problems in LMM. No associations were observed for BPA or phthalate metabolite concentrations and externalizing problem scores as reported by parents and there was no indication of sex differences by interaction terms or in stratified analyses.

A doubling of creatinine-adjusted prenatal mBP concentration was associated with higher self-reported externalizing problems (B = 0.08 per 2-fold increase in creatinine-adjusted prenatal mBP, 95% CI: 0.01, 0.16), with no indication for sex differences. Additionally, higher mIBP concentrations were associated with higher externalizing problems (B = 0.08, 95% CI: 0.01, 0.16), particularly in boys (B = 0.12, 95% CI: 0.03, 0.20). In boys only, higher mCMHP (B = 0.19, 95% CI: 0.08, 0.30) and mECPP (B = 0.17, 95% CI: 0.07, 0.28) were associated with higher self-reported externalizing problem scores. In contrast, higher PA concentrations were associated with lower self-reported externalizing problem scores (B = − 0.09, 95% CI: − 0.17, − 0.01), with no indication for sex differences by interaction terms or in stratified analyses. The associations between mIBP, mECPP and mCMHP concentrations and higher self-reported externalizing problems in boys survived multiple testing correction. No other associations were found for prenatal phthalate or BPA exposure and self-reported externalizing problem scores.

### 3.3. Childhood exposure to bisphenols and phthalates

#### 3.3.1. Internalizing problems

**Fig. 3.**
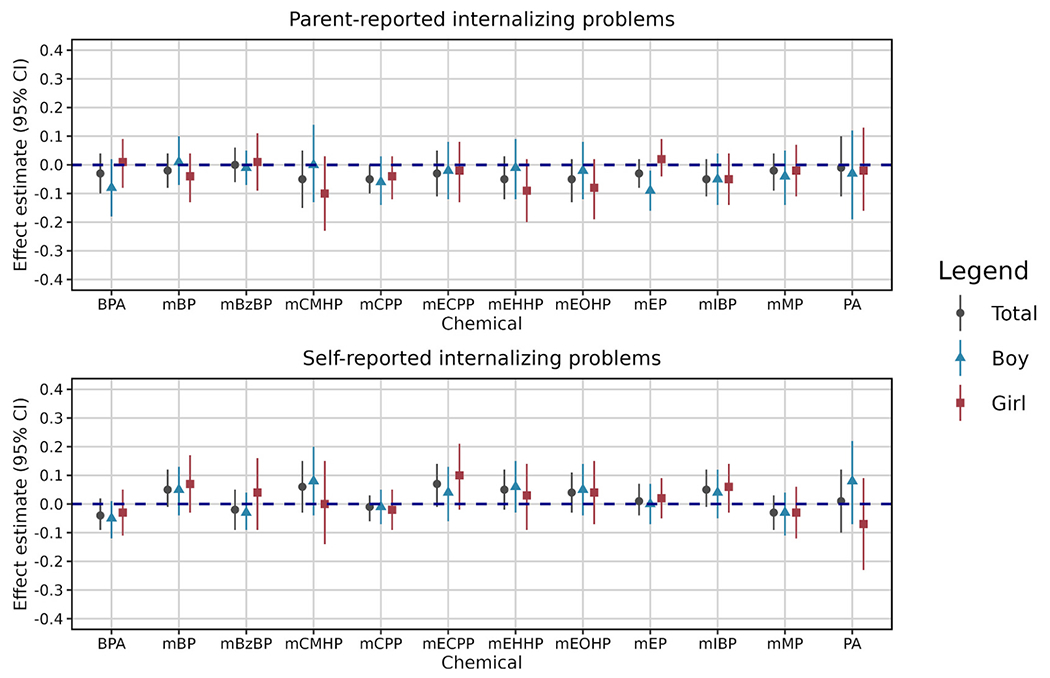
Associations of childhood BPA and phthalate exposure with internalizing problem score through age 14 years. *Figure note*: Models were adjusted for maternal age, pre-pregnancy body mass index, parity, country of origin, maternal educational levels, marital status, maternal smoking and alcohol drinking habits and urinary concentrations of childhood organophosphate pesticides, as well as gestational age at birth, birthweight, child sex (only in models with all children) and child age at outcome measurement.. Effect estimates are reported per log-2 unit increase in creatinine adjusted concentrations of childhood BPA and phthalate metabolites. Internalizing problem scores for both parent-reports and self-reports were obtained at ages 10, and 14 years, and standardized. Vertical lines represent 95% Confidence Intervals.. Based on models including interactions between sex and single chemicals, significant interaction terms (p < 0.10) were observed for: Parent-reported internalizing problems – mEP; Self-reported internalizing problems – none.. Based on models including interactions between age and single chemicals, significant interaction terms (p < 0.10) were observed for: Parent-reported internalizing problems – BPA, mMP; Self-reported internalizing problems – BPA.

Overall, there were no associations between childhood exposure to phthalates or BPA and parent-reported child internalizing problem scores or self-report measures, as shown in Fig. 3. The two exceptions were that higher childhood mEP concentrations were associated with lower parent-reported child internalizing problem scores in boys only (B = − 0.09 per 2-fold increase in creatinine-adjusted child mEP, 95% CI: − 0.16, − 0.02). Second, higher mECPP levels were associated with higher self-reported child internalizing problem scores in girls only (B = 0.11, 95% CI: 0.00, 0.22). These findings did not survive multiple testing correction.

#### 3.3.2. Externalizing problems

**Fig. 4.**
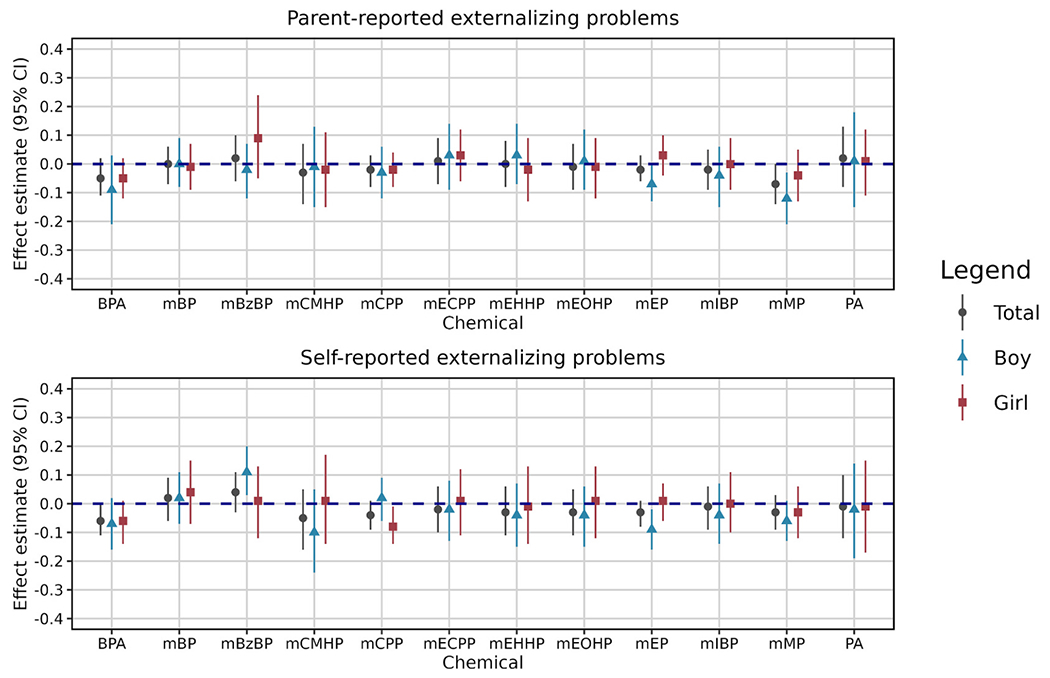
Associations of childhood BPA and phthalate exposure with externalizing problem score through age 14 years.. *Figure note*: Models were adjusted for maternal age, pre-pregnancy body mass index, parity, country of origin, maternal educational levels, marital status, maternal smoking and alcohol drinking habits and urinary concentrations of childhood organophosphate pesticides, as well as gestational age at birth, birthweight, child sex (only in models with all children) and child age at outcome measurement.. Effect estimates are reported per log-2 unit increase in creatinine adjusted concentrations of childhood BPA and phthalate metabolites. Externalizing problem scores for both parent-reports and self-reports were obtained at ages 10, and 14 years, and standardized. Vertical lines represent 95% Confidence Intervals.. Based on models including interactions between sex and single chemicals, significant interaction terms (p < 0.10) were observed for: Parent-reported externalizing problems – mMP; Self-reported externalizing problems – mEP. Based on models including interactions between age and single chemicals, significant interaction terms (p < 0.10) were observed for: Parent-reported externalizing problems – mECPP, mEOHP; Self-reported externalizing problems – none.

Fig. 4 illustrates associations of child chemical exposure and child externalizing problem scores. In boys only, higher childhood mEP concentrations were associated with lower parent-reported child externalizing problem scores (B = − 0.06 per 2-fold increase in creatinine-adjusted child mEP, 95% CI: − 0.12, 0.00). Similarly, higher mMP concentrations were associated with lower parent-reported child behavior problem scores (B = − 0.07, 95% CI: − 0.14, 0.00), which was mainly driven by associations in boys (B = − 0.12, 95% CI: − 0.22, − 0.03). The association of mMP concentrations with higher parent-reported externalizing problems in boys survived multiple testing correction. No associations were found for other childhood phthalate or BPA concentrations with parent-reported externalizing problem scores.

Further, Fig. 4 demonstrates associations of child phthalate concentrations and self-reported externalizing problems. We found an association between higher childhood mEP concentrations and lower externalizing problems in boys only (B = − 0.08 per 2-fold increase in creatinine-adjusted child mEP, 95% CI: − 0.15, − 0.01). Higher mCPP concentrations were associated with lower externalizing problem scores in girls only (B = − 0.07, 95% CI: − 0.14, − 0.00). In contrast, a doubling of creatinine-adjusted childhood mBzP concentration was associated with higher externalizing problem scores in boys only (B = 0.12, 95% CI: 0.04, 0.20). We further observed that higher childhood BPA was associated with lower externalizing problem scores (B = − 0.06, 95% CI: − 0.12, 0.00), with associations mainly driven by girls (B = − 0.09, 95% CI: − 0.17, − 0.01) in stratified analyses. The association of childhood mBzP concentrations with higher self-reported externalizing problems in boys survived multiple testing correction.

### 3.4. Additional analyses

Results of mixture analyses by quantile g-computation showed positive, but non-significant, trends in the overall associations of the prenatal chemical mixture with both internalizing and externalizing problem scores (Figs. S5–S6). Furthermore, mMP and mEHHP were the main contributing chemicals in the overall associations of the prenatal chemical mixture and internalizing problem scores in the total sample and in girls specifically. Further, positive weights were observed for prenatal mBP levels, among others, and internalizing problem scores in girls specifically (Fig. S7), suggesting a trend for higher internalizing problem scores with higher exposure to these chemicals. Additionally, BPA and mIBP were the main contributing chemicals in the associations between the prenatal chemical mixture and externalizing scores in the total sample, while positive weights were also observed for prenatal mIBP and mCMHP levels, among others, in the total analysis sample and in boys and girls separately (Fig. S8). These results suggested a trend for higher exposure and higher externalizing problem scores with higher exposure to chemicals. Exclusion of the DAP molar sum as a covariate from analyses did not change associations substantially (Figs. S9–S12). When childhood BPA and PA concentrations were removed from pregnancy exposure LMMs, coefficients remained largely the same compared to when childhood BPA and PA were included, only 95% CI’s became wider (Figs. S13–S14). When pregnancy BPA and PA concentrations were removed from childhood exposure LMMs, both coefficients and 95% CI’s remained largely the same (Figs. S15–16). We observed no interaction between age at outcome assessment and exposure, indicating that associations between exposure and internalizing or externalizing problem scores did not differ by age at outcome assessment (data not shown). There was also no indication for a non-linear relationship between BPA and/or phthalate concentrations and internalizing/externalizing problem scores (Supplementary Tables S5–S12). The complete case analyses overall showed similar patterns of associations and directions of effect estimates as compared to the main (adjusted) models, with less associations reaching significance in the complete case analyses (Figs. S17–S20).

## 4. Discussion

We performed a longitudinal study of prenatal and childhood exposure to common plasticizers, i.e. BPA and phthalates, and assessed internalizing and externalizing outcomes from childhood into adolescence, incorporating important confounders and co-exposures with potential neurotoxicity. We applied a dual-informant approach to obtain information on children and adolescent internalizing and externalizing problems. Overall, associations between chemical exposure data and internalizing and externalizing problem scores from early childhood to adolescence were largely null, since most did not withstand multiple testing correction. Additionally, effect estimates should be interpreted as relative differences within the population rather than in relation to clinical cut-offs, since *Z*-scores where used for internalizing and externalizing problems, but not T-scores. However, our data showed a few consistent associations with prenatal phthalate exposures, mainly present for child self-reported outcomes, with higher levels of mBP and mEHHP associated with more internalizing problems in girls, and higher levels of mIBP, mECPP and mCMHP associated with more externalizing problems in boys. However, findings with regard to childhood exposure to phthalates were less consistent with no clear differences between boys and girls as compared to prenatal exposure, and largely did not withstand multiple testing correction. We found no association for both prenatal and childhood BPA exposure with internalizing and externalizing problems in children and adolescents. Despite positive trends in the overall effects of the prenatal exposure mixture on both internalizing and externalizing problem scores at age 14 years, these associations did not reach significance. Mixture analyses demonstrated positive weights for prenatal mBP and mEHHP levels related to internalizing problems in girls, and for metabolites prenatal mIBP and mCMHP levels related to externalizing problems in boys, girls and in the total sample. Notably, these metabolites also showed positive betas in single pollutant analyses, indicating consistent directionality. This may suggest that while the combined effects were not significant, the individual metabolites could play a role in behavioral problems, warranting further investigation.

In comparison to previous literature, it is not surprising that associations of BPA and phthalate exposure with child internalizing and externalizing problem scores were generally absent. For instance, recent systematic reviews concluded that there was little evidence to support associations of prenatal and childhood BPA and phthalate exposure with neurodevelopmental outcomes, including internalizing and externalizing problems (Minatoya and Kishi, 2021; Antoniou and Otter, 2024). The null associations may reflect biological complexities and methodological limitations. Among these is exposure misclassification, since phthalates and bisphenols are non-persistent chemicals and single spot urine samples in a particular time window may lead to measurement error (Perrier et al., 2016). While urinary phthalate and bisphenol concentrations can vary significantly depending on the time of day the sample is collected, the variation in sampling timing is unlikely to lead to differential misclassification related to the outcomes of interest (internalizing and externalizing problems). The timing of urine collection is unlikely to be systematically related to the child’s behavioral or emotional problems. However, timing variation could still reduce the precision of our exposure measures, and this non-differential misclassification could attenuate true associations, potentially contributing to the null findings observed in our study. On the biological level, there may be periods of heightened susceptibility for adverse health effects of BPA and phthalate exposure. If indeed phthalate sensitivity is limited to specific developmental windows, studies may be unable to capture associations, since chemical exposure is measured at various time points which could be outside the window of susceptibility for neurodevelopment. Additionally, the CBCL and YSR instrument may not be sensitive enough to capture associations of environmental exposure which may be too subtle (McClendon et al., 2011).

Our limited findings on prenatal phthalate exposure with internalizing problems in offspring partly align with previous literature. For example, three studies found associations of prenatal mMP, mBP, mEP and mBzP with anxiety, depression and internalizing problems in children (Huang et al., 2024; Engel et al., 2010; Guilbert et al., 2021). However, internalizing problems were assessed at a much younger age in these studies (e.g. at age two years), limiting comparability to later developmental stages. The study by Chen et al. found associations between higher DEHP exposure, measured in single spot urine samples, and higher internalizing problem scores in children aged 8–14 years (Chen et al., 2019), which is similar to the age range of participants in our study. Although sex-stratified analyses were performed, the relatively small study sample of 122 children may have limited the ability to detect sex-specific differences and they focused only on a single phthalate group. A recent study by Oh et al. found largely null-associations between phthalate exposure and internalizing problems (Oh et al., 2025), which is partly in alignment with the current results. Notably, Oh et al. is one of the few studies that assessed prenatal exposure to the alternative plasticizer cyclohexane-1,2-dicarboxylic acid mono carboxyisooctyl ester (DINCH) next to phthalate exposure (Oh et al., 2025). Though, they found no associations between prenatal DINCH exposure and child externalizing problems, previous studies demonstrated that DINCH is associated with endocrine disrupting activity and is capable of modifying thyroid function (Cathey et al., 2019; Yu et al., 2023; Morales-Grahl et al., 2024). The relevance of this activity for externalizing development, combined with the rising exposure to DINCH and other alternative plasticizers, underscores the need for future research to focus on large-scale epidemiological studies investigating the effects of alternative plasticizers on children’s behavior.

Our study builds on previous findings by extending the assessment age through adolescence and relying on child self-reported internalizing problems rather than solely the parent-report. On average, adolescent girls may better recognize and report their emotions (He et al., 2023; Chaplin and Aldao, 2013), which could explain why we do find a few associations with self-reported internalizing problems. Additionally, during adolescence internalizing problems such as depression and anxiety become more prevalent in girls (Apicella et al., 2025), which could have increased our statistical power to detect associations in girls. Importantly, we found few associations for prenatal phthalate exposure, but associations for childhood phthalate exposure were largely absent and did not withstand multiple testing correction. This contrast supports the idea that brain development occurring during the prenatal stages of life could be uniquely sensitive to environmental exposures such as phthalates (Grandjean and Landrigan, 2014; Rice and Barone Jr., 2000). Overall, our findings suggest that prenatal and childhood phthalate exposure show limited to no associations with internalizing problems, which aligns with the broader, inconsistent literature on this topic and highlights the complexity of detecting consistent associations in developmental research.

Consistent with some prior studies, we also found limited associations between prenatal phthalate exposure and externalizing problems, albeit involving different phthalates. Like in our study, prenatal mIBP exposure was previously associated with aggression, rule-breaking behavior and conduct problems (England-Mason et al., 2020a; Kobrosly et al., 2014), specifically in boys (Oh et al., 2025), though this association did not withstand multiple testing correction in our study. Other studies reported associations with other prenatal phthalate metabolites, such as mCPP and mEP, rather than mECPP or mCMHP in this study (Minatoya et al., 2018; Guilbert et al., 2021). The study by Minatoya et al. used a single phthalate measurement and assessed externalizing problems in children (*n* = 458) aged 5 years and found no associations (Minatoya et al., 2018). At this age, and prior to school age, some externalizing problems may yet not have emerged. This, together with a relatively small sample size, may explain the largely null findings. Hyland et al. measured externalizing problems repeatedly until children were 16 years of age and found no significant associations with prenatal phthalate exposure measured across two time points in pregnancy (Hyland et al., 2019). However, only parent-reported externalizing problem scores were used, which arguably could lead to different results, since our associations were mostly found with child self-reported scores. Additionally, their sample had higher phthalate concentrations compared to our study. This could reflect a non-monotonic dose-response relationship, most likely an inverted U-shaped curve, where moderate exposures may be more harmful than higher ones. This type of relationship has previously been suggested for phthalate toxicity (Astuto et al., 2023; Fauziah et al., 2023) and supported by a preclinical study (Oudir et al., 2018). Hyland et al. did find a significant association between prenatal mECPP concentrations and conduct problems, similar to ours. Interestingly, parent reports are generally more reliable for externalizing problems, particularly in younger aged children, whereas self-reports become increasingly informative for internalizing problems, when children grow into adolescence (Bartels et al., 2003; Chen et al., 2017), which highlights the utility of youth reports for some externalizing and internalizing traits in youth. Hyland et al. reported no sex differences, although this may be reflective of the small sample size. Our sample size provided sufficient power to test hypotheses on sex-specific associations between phthalate exposure and adolescents’ internalizing and externalizing problems. Moreover, our findings support the hypothesis that associations of phthalates with internalizing and externalizing problems may be sex-specific. Overall, previous studies often relied on a single prenatal urine sample, assessed outcome in early childhood, used only parent reports, and had modest sample sizes. These factors may all contribute to inconsistencies in findings on internalizing and externalizing problems, both compared to our study and more broadly in the field.

With regard to childhood exposure to EDCs, some studies found associations between mEP measured in children aged 1–8 years and internalizing and externalizing problems (Li et al., 2020a; Daniel et al., 2020), which differs from our study. Huang et al. reported associations between age 1.5–3 years mBP and mBzP levels in a single spot urine sample and higher internalizing problems at the same age (Huang et al., 2024). In contrast, a study with a longitudinal design found no associations for age 6–13 years mEP levels, despite similar or higher phthalate concentrations as compared to our study (Huang et al., 2019). Daniel et al. assessed phthalate exposure in children at two time points; at ages 3 and 5 years, and found that non-DEHP metabolites were associated with internalizing problems in boys and DEHP metabolites were associated with externalizing problems in girls, both at age 7 years. This study differed from ours by measuring internalizing and externalizing problems at a younger age and having a smaller sample size (*n* = 411), which may have contributed to differences in the findings.

Overall, literature reporting on childhood phthalate exposure and behavioral functioning remains inconsistent. This inconsistency may be partly due to the frequent use of cross-sectional designs (Huang et al., 2024; Hu et al., 2017; Jankowska et al., 2019), small sample sizes and measuring phthalate exposure in single spot urine samples. In contrast, in our longitudinal study, associations with childhood exposure were less pronounced, suggesting that early childhood may not be a particularly sensitive period for phthalate effects on brain and behavior. However, these findings should be interpreted with caution due to our smaller childhood sample size and reliance on a single exposure measurement, both of which may have introduced measurement error and reduced statistical power. Particularly, non-differential misclassification from single-point measurement could attenuate true associations (Armstrong, 1998). To better understand the role of exposure timing, future research should include exposure measurements in different childhood developmental windows such as pre- and peri-adolescence, while accounting for prenatal exposure, to capture and isolate potential important exposure periods more accurately.

We found no associations between prenatal or childhood BPA exposure and internalizing or externalizing problems. Other studies have found associations, particularly with internalizing problems (Li et al., 2020a; Grohs et al., 2019; Perez-Lobato et al., 2016; Roen et al., 2015), which may be due to higher BPA concentrations in their samples. The null result for BPA contrasts with experimental studies showing that BPA disrupts hormonal and neurodevelopmental processes, including synapse plasticity and neural stem cell proliferation (Welch and Mulligan, 2022). This could in turn affect emotional and behavioral development. Translating findings from animal models to human populations remains challenging with varying exposure levels and individual differences.

Among the limited reported significant associations, a consistent pattern was reported of associations for prenatal phthalate exposure with internalizing problems in girls and externalizing problems in boys. Notably, sex-stratified internalizing and externalizing problems showed no differences for parent-reported scores, whereas self-reported slightly higher internalizing problems in girls and slightly higher externalizing problems in boys. Overall, this is in line with prior literature (Sappenfield et al., 2018). Importantly, parent- and self-reports were collected at different ages, hence these differences could reflect age-related developmental changes, which aligns with literature showing that sex differences in internalizing symptoms typically emerge after onset of puberty (Hankin, 2009). These baseline sex-differences are important when interpreting our sex-specific results. However, because sex was included as a confounder and analyses were stratified, these baseline differences are unlikely to fully explain the observed sex specific associations. Moreover, internalizing and externalizing problem scores were extended into adolescence in the present study. Together, an important consideration, when interpreting the sex-specific pattern is the potential mediating role of pubertal status. Puberty is an important developmental stage that involves considerable hormonal changes that affect the adolescent brain (Peper and Dahl, 2013). Bisphenols and phthalates on the other hand, are endocrine disrupting chemicals potentially affecting the human sex hormone systems, for associations with puberty status have been demonstrated (Berger et al., 2018; Wolff et al., 2014). Therefore, prenatal BPA and phthalate exposure could interact with puberty-related brain changes, particularly in relation to sex hormone levels. In turn, this interaction may account partially for the observed associations with internalizing and externalizing problems. To capture puberty as a mediator in the reported associations, a longitudinal dataset is required containing repeated measures on both internalizing and externalizing problems, as well as on puberty status. This type of data is beyond the scope of our present study. However, the proposed analyses represent and interesting and important direction for future research, aimed at understanding the causal pathway of associations between prenatal BPA and phthalate exposure with internalizing and externalizing behaviors.

### 4.1. Potential mechanisms

Experimental research showed that bisphenols and phthalates disrupt the sex hormone system (Engel et al., 2017; Amir et al., 2021; Pathak et al., 2024; Radke et al., 2018; Moche et al., 2021), potentially contributing to sex differences in brain influences of these chemicals. Alterations of the hormonal milieu could affect both prenatal and childhood brain development (Auyeung et al., 2013; Miranda and Sousa, 2018; Servin-Barthet et al., 2025). Animal research has further shown that bisphenols and phthalates interact with the thyroid hormone system (Morales-Grahl et al., 2024; Hu et al., 2023), which is another important hormonal system for neurodevelopment (Williams, 2008; Ghassabian et al., 2014; Korevaar et al., 2016). In line with animal experimental data, epidemiological studies showed adverse effects of phthalates and bisphenols on thyroid function in both pregnant women and in children (Zhu et al., 2024; Liu et al., 2024; Maia and Vieira-Coelho, 2024; Derakhshan et al., 2021). Additionally, some human studies showed that prenatal and early-childhood exposure to both phthalates and BPA was associated to alterations in brain structure and subsequently internalizing or externalizing behavioral problems (England-Mason et al., 2020b), providing another potential mechanism.

Yet, we did not observe evidence for associations of BPA exposure with internalizing and externalizing problems in this study. This could be because thyroid and sex hormone disruption may be more closely linked to cognitive brain outcomes than to behavioral functioning, as some studies find associations between thyroid hormones and IQ (Ghassabian et al., 2011), whereas another study did not find an association of maternal thyroid hormones with internalizing and externalizing problems (Fetene et al., 2020). Additionally, the timing of EDC exposure in our study might not have coincided with sensitive windows for thyroid disruption or brain development. To illustrate, one study found that maternal thyroid function at 8 weeks of gestation was associated with brain morphology and (Jansen et al., 2019). Further, findings on thyroid disruption by EDCs from animal experiments are not replicated in humans, as seen in the case of OP pesticides, another class of EDCs that disrupt thyroid signaling (Campos and Freire, 2016), but showed no effects in the Generation R cohort (Mulder et al., 2019). Lastly, any potential influences of phthalates and bisphenols on behavioral problems might not be lasting through adolescents as earlier studies did not have follow ups beyond childhood.

### 4.2. Strengths and limitations

This study is unique in including repeated measures of internalizing and externalizing problems from early childhood through adolescence and incorporating exposures to phthalates and bisphenols during two critical developmental windows: the prenatal period and early childhood. The prenatal period is often considered a sensitive window for environmental influences such as EDCs, due to the potential effects on fetal brain development (Sarigiannis et al., 2021; Oskar et al., 2024; Bellinger, 2013). Childhood exposure to bisphenols and phthalate might influence internalizing and externalizing outcomes through ongoing endocrine disruption and ongoing development of brain processes, such as emotion regulation, impulsivity and (social) cognition. Assessing both periods allows for a more comprehensive understanding of timing effects. Additional strengths include a large sample size that allowed examination of associations in boys and girls separately, and including important confounders and co-exposures.

However, the following limitations should be considered when interpreting our results. First, measurement error may lead to exposure misclassification, particularly for childhood exposure as we only had one spot urine sample for this window. In addition, we lacked information on the timing of spot urine sampling, which could affect chemical concentrations (Boeniger et al., 1993; Barr et al., 2005). Although timing variations are unlikely to confound associations, information on the exact time of day of sampling could have improved precision. This is especially relevant considering that phthalates and bisphenols are non-persistent chemicals, and timing variations may introduce non-differential misclassification, which is unlikely to bias the direction of associations but may lead to imprecision and potentially contribute to null results. Third, we adjusted for urinary dilution using creatinine concentration. Creatinine adjustment is advantageous because of the low costs, relative measurement easiness and the wide availability of assays (Johnson et al., 2014). However, using specific gravity could be more suitable, since creatinine levels may vary over pregnancy (MacPherson et al., 2018; Abduljalil et al., 2012; Davison et al., 1980; Cheung and Lafayette, 2013). In addition, creatinine levels may be more affected by age, gender, body size and meat intake as compared to specific gravity (Suwazono et al., 2005). Finally, residual confounding by unmeasured factors (e.g. exposure to heavy metals, triclosan, parabens and other EDCs) cannot be ruled out.

## 5. Conclusion

To conclude, associations of BPA and phthalate exposure with internalizing and externalizing problem scores in adolescents were largely null. However, we found that prenatal exposure to a few specific phthalate metabolites was associated with internalizing and externalizing problems through adolescence, with higher internalizing problems in girls and higher externalizing problems in boys. These findings suggest that the prenatal period is a potentially important window for some phthalates influencing neurodevelopment. The observed sex-specific associations point to possibly moderating roles of biological or hormonal systems, specifically sex-hormones, that should be further investigated in future research. Our study addresses a gap in the literature by being one the first studies with follow-up through adolescence, which should be replicated. Future research should build on the current work by measuring bisphenol and phthalate concentrations in different childhood exposure windows such as pre- and peri-adolescence, while accounting for prenatal exposure. Additionally, potential involved mechanisms should be explored in large population-based cohorts such as generation R, for example by measuring sex and thyroid hormone, and assessing brain MRI scans to study brain structural and functional alterations.

## Supplementary Material

Supplementary Materials

## Figures and Tables

**Table 1:** Characteristics of Generation R study participants.

	Study population (*n* = 1361)	Population at baseline (*n* = 9901)
Maternal characteristics Age (median (IQR); in years)	31 (27.6–34)	30.5 (26.2–33.8)
BMI (median (IQR); in kg/m^2^)	22.7 (20.8–25.3)	22.6 (20.8–25.4)
Parity (percentage)		
0	61.2	54.9
1+	38.7	45.1
Country of origin (percentage)		
Netherlands	54.9	50.6
Morocco	5.4	6.5
Suriname	8.8	8.8
Turkey	7.5	8.7
Other European	8.3	8
Other	15.1	17.4
Education level^a^ (percentage)		
Low/Intermediate	48.4	56.9
High	51.6	43.1
Household income (percentage)		
<€1200,-	7.9	12.2
€1200,- to €2000,-	18.3	21.8
>€2000,-	73.8	66.1
Marital status (percentage)		
Married/Living with partner	89.1	85.7
No partner	10.9	14.3
Maternal smoking		
No smoking	75.3	73.5
Until pregnancy recognized	10.5	8.6
Continued during pregnancy	14.2	17.9
Maternal alcohol (percentage)		
Never drank alcohol	40	47.9
Until pregnancy recognized	17.3	13.3
Continued during pregnancy occasionally	36.4	31.6
Continued during pregnancy frequently	6.2	7.3
Gestational age per period^b^		
Early pregnancy (median (IQR); in weeks)	12.9 (12.1–14.4)	13.2 (12.2–14.9)
Mid pregnancy (median (IQR); in weeks)	20.4 (19.9–20.9)	20.4 (19.9–21.2)
Late pregnancy (median (IQR); in weeks)	30.2 (29.9–30.8)	30.2 (29.9–30.8)
Child characteristics		
Sex, female (percentage)	49.4	49.4
Gestational age at birth (median (IQR); in weeks)	40.3 (39.3–41)	40 (38.9–40.9)
Age at research visit (median (IQR); in years)	5.8 (5.7–5.9)	5.9 (5.8–6.2)
Birthweight (median (IQR); in grams)	3450 (3120–3770)	3420 (3070–3765)

*Table note*: Medians and inter quartile ranges are reported for continuous variables and percentages are reported for categorical variables. The proportion of missing data in the analytical sample was 11.6% for BMI, 0.5% for parity, 0.6% for country of origin, 3.9% for education level, 14.3% for household income, 4.6% for marital status, 9.2% for maternal smoking, 6.4% for maternal alcohol and 8.7% for child age at research visit. BMI = Body Mass Index.

a Low/Medium: No education finished/primary education only/Secondary education; High: university degree or higher vocational training.

b The gestational age windows are the gestational ages during urine sampling. These gestational age windows do not exactly correspond to trimester divisions, hence the labelling “early pregnancy”, “Mid pregnancy” and “Late pregnancy”. Early pregnancy is defined as a gestational age of <18 weeks, mid pregnancy as a gestational age of 18–25 weeks, and late pregnancy as a gestational age of >25 weeks. For each collection window, the median gestational age of the participants within that window is presented.

**Table 2:** Descriptive information on urinary bisphenol and phthalate metabolite concentrations in pregnancy and childhood.

	<18 weeks pregnancy		18–25 weeks pregnancy		> 25 weeks pregnancy		Childhood, age 6 years	
	Median (IQR) (ng/mL)	% < LOD	Median (IQR) (ng/mL)	% < LOD	Median (IQR) (ng/mL)	% < LOD	Median (IQR) (ng/mL)	% < LOD
BPA	1.7 (0.7–3.6)	21.3	1.4 (0.8–3.2)	7.8	1.7 (0.9–3.3)	10.9	0.8 (0.5–1.5)	26.2
BPS	0.4 (0.2–1.1)	33.6	0.2 (0.1–0.5)	70.6	0.6 (0.2–1.4)	80.7	0.2 (0.1–0.4)	75
BPZ	0.2 (0.1–0.2)	87.5	0.2 (0.1–0.2)	96.2	1.1 (1–1.2)	99.9	–	100
BPB	0.2 (0.1–0.3)	90.5	0.1 (0.1–0.2)	97.8	–	100	–	100
BPF	0.6 (0.3–1.3)	60.1	0.5 (0.3–1.3)	88.6	1.2 (0.7–2.1)	71.2	0.5 (0.3–0.6)	93
BPAP	0.2 (0.1–0.4)	92.5	–	100	0.2 (0.2–0.2)	99.9	0.2 (0.2–0.2)	99.9
BPAF	–	100	–	100	3.7 (3.7–3.7)	99.9	–	100
BPP	0.2 (0.1–0.3)	90.5	0.1 (0.1–0.2)	97.8	–	100	–	100
mMP	5.4 (2.7–9.9)	0.8	3.5 (1.8–6.3)	1.1	4.1 (2–8)	1.4	3.7 (1.6–6.6)	17.3
mEP	138.6 (41–486.8)	0.7	73.1 (25.1–224.5)	1	129.9 (44.8–409.8)	0.9	15.4 (6.9–37.3)	0
mCPP	1.4 (0.8–2.8)	0.6	0.9 (0.5–1.7)	1	1.8 (1–3.1)	1	1.3 (0.7–2.5)	0
mIBP	21 (9.5–45.2)	0.8	9 (4.6–18.1)	1	17.7 (9.2–37.7)	1.2	14.6 (8.6–27.5)	0.5
mBP	16.1 (6.8–30.9)	1.4	9.7 (5.5–19.1)	1	12 (6.1–24.6)	1.1	7.1 (3.6–13.1)	2.1
mECPP	16.2 (8.1–31.6)	0.8	10.6 (5.7–20.1)	1.1	18.1 (9.5–33.8)	0.9	9 (4.9–17.3)	0
mCMHP	14 (7.6–26.3)	0.7	4.1 (2.3–7.4)	1.2	3.5 (1.9–6.5)	2	4.1 (2.5–6.8)	0
mEHHP	12 (5.7–23.2)	0.8	5.6 (3–10.9)	1.1	10.1 (5.1–19.7)	1.1	7.3 (4.1–12.6)	0
mBzP	6.5 (3–12.6)	8.8	5.4 (2.3–11.2)	2.6	3.2 (1.3–6.4)	4.4	2.9 (1.4–6.5)	40.4
mINP	0.8 (0.4–1.7)	86.4	0.2 (0.2–0.4)	98.6	16.9 (16.9–16.9)	99.9	–	100
mCHP	0.2 (0.1–0.4)	81.3	0.1 (0–0.1)	94.5	0.3 (0.2–1.3)	99.4	0.1 (0.1–0.1)	95.5
mOP	0.5 (0.3–0.8)	90.3	0.6 (0.5–0.7)	99.5	0.4 (0.4–0.7)	99.4	–	100
PA	56.9 (30.6–122.1)	0.9	153.5 (61.8–286.4)	1.1	69.5 (34.2–132.8)	1.4	27.8 (17.8–43.8)	0
mIDP	1.9 (1.3–2.7)	92.8	1.2 (1.1–1.5)	98.2	3 (2.1–5.6)	96.9		100
mHxP	0.3 (0.2–0.6)	24.2	0.1 (0.1–0.2)	98.9	0.7 (0.4–1.2)	98.1	0.3 (0.1–0.5)	95.9
mHpP	1.1 (0.6–2.3)	35.9	0.6 (0.4–0.7)	96.8	1.4 (0.7–2.5)	98.6	–	100
mCHpP	0.1 (0.1–0.1)	99.2	–	100	1.5 (0.9–2.6)	99.8	–	100
mEOHP	7.8 (3.5–15.4)	0.6	7.6 (3.7–16.6)	1	7.2 (3.8–14)	1.1	4 (2.2–7)	0

*Table note*: Descriptive statistics were computed using the observed chemical concentrations. For every chemical the median observed concentration in ng/mL together with the Inter Quartile Range (IQR) is reported. Further, the percentage of chemical concentrations below the Limit Of Detection (LOD) is reported. Chemicals with 30% or more concentrations below LOD were excluded from analysis. The Intra Class Correlation (ICC) was computed for the three pregnancy concentrations of every chemical, using a two-way mixed-effects model with a single measurements and absolute agreement.

BPA: Bisphenol A; BPS: Bisphenol S; BPZ: Bisphenol Z; BPB: Bisphenol B; BPF: Bisphenol F; BPAP: Bisphenol AP; BPAF: Bisphenol AF; BPP: Bisphenol P; mMP: mono-methyl phthalate; mEP: mono-ethyl phthalate; mCPP: mono(3-carboxypropyl) phthalate; mIBP: mono-isobutyl phthalate; mBP: mono-n-butyl phthalate; mECPP: mono-(2-ethyl-5-carboxypentyl) phthalate; mCMHP: mono-[(2-carboxymethyl)hexyl]phthalate; mEHHP: mono-(2-ethyl-5-hydroxyhexyl)phthalate; mBzP: mono-benzyl phthalate; mINP: monoisononylphthalate; mCHP: mono-cyclohexyl; mOP: monooctylphthalate; PA: phthalic acid; mIDP: mono-hydroxy-isodecyl phthalate; mHxP: mono-hexylphthalate; mHpP: mono-2-heptylphthalate; mCHpP: mono(7-carboxyheptyl); mEOHP: mono-(2-ethyl-5-oxohexyl) phthalate.

**Table 3:** Outcome descriptives for the study population.

	Study population (n = 1361)					
	Raw scores			*Z*-scores		
	Total sample	Boys	Girls	Total Sample	Boys	Girls
Parent reported outcome (CBCL)						
Internalizing problem scores						
3 years (median (IQR))	4 (2–7)	4 (2–7)	3 (1–7)	−0.2 (−0.6–0.4)	−0.2 (−0.6–0.4)	−0.4 (−0.8–0.4)
6 years (median (IQR))	4 (2–8)	4 (2–9)	4 (2–8)	−0.3 (−0.7–0.3)	−0.3 (−0.7–0.5)	−0.3 (−0.7–0.3)
10 years (median (IQR))	3 (1–7)	3 (1–7)	3 (1–7)	−0.4 (−0.8–0.4)	−0.4 (−0.8–0.4)	−0.4 (−0.8–0.4)
14 years (median (IQR))	4 (2–8)	4 (1–7.8)	4 (2–8)	−0.3 (−0.6–0.4)	−0.3 (−0.8–0.4)	−0.3 (−0.6–0.4)
Externalizing problem scores						
3 years (median (IQR))	8 (4–12)	8 (4–12.6)	7 (3–11)	−0.1 (−0.7–0.6)	−0.1 (−0.7–0.7)	−0.2 (−0.9–0.4)
6 years (median (IQR))	6 (3–11)	7 (3–12)	5 (2–9.4)	−0.2 (−0.8–0.5)	−0.1 (−0.7–0.7)	−0.4 (−0.8–0.3)
10 years (median (IQR))	2 (0–5)	3 (1–6)	2 (0–4)	−0.4 (−0.8–0.2)	−0.2 (−0.6–0.4)	−0.4 (−0.8–0)
14 years (median (IQR))	2 (0–6)	3 (1–7)	2 (0–5)	−0.4 (−0.8–0.3)	−0.2 (−0.6–0.5)	−0.4 (−0.8–0.1)
Self-reported outcome						
Internalizing problem scores						
10 years (BPM—Y; (median (IQR)))	2 (1–3)	1 (0–3)	2 (1–4)	0.1 (−0.6–0.4)	−0.6 (−1–0.4)	−0.1 (−0.6–0.9)
14 years (YSR; (median (IQR)))	7 (3–13)	6 (3–10)	9 (5–14.8)	−0.2 (−0.8–0.6)	−0.4 (−0.8–0.2)	0 (−0.5–0.8)
Externalizing problem scores						
10 years (BPM—Y; (median (IQR)))	2 (0–3)	2 (0–3)	1 (0–3)	0.0 (−1.0–0.6)	0 (−1–0.6)	−0.5 (−1–0.6)
14 years (YSR; (median (IQR)))	6 (3–10)	7 (4–11)	6 (3–9)	−0.2 (−0.8–0.6)	0 (−0.6–0.7)	−0.2 (−0.8–0.4)

*Table note*: Medians and inter quartile ranges are reported for raw scores and Z-scores for internalizing and externalizing problems. Parent reported internalizing problem scores (e.g emotional problem scores) and externalizing problem scores (e.g. behavioral problem scores) are derived from the Child Behavioral Checklist (CBCL) preschool version at 3 and 6 years and school-age version at 10 and 14 years. Self-reported internalizing problem scores (e.g emotional problem scores) and externalizing problem scores (e.g. behavioral problem scores) are derived from the Brief Problem Monitor-Youth (BPM-Y) at 10 years and from the Youth Self-Report (YSR) at 14 years. Z-scores were calculated for harmonization across instrument versions, raters and ages. To calculate Z-scores, the mean is subtracted from the raw score, which is then divided by the standard deviation. Applying this calculation to every raw score yield a standard normal distribution.

## Data Availability

Data used in these analyses are available upon reasonable request from the corresponding author

## HIGHLIGHTS

- This study combined repeated pregnancy exposure with childhood exposure.
- Emotional and behavioral problems were repeatedly measured using two reporters.
- In girls, prenatal mBP and mEHHP levels were related to more emotional problems.
- In boys, prenatal mECPP and mCMHP levels were related to more behavioral problems.
- No associations were found for prenatal and childhood BPA or childhood phthalates.

## Appendix A. Supplementary data

Supplementary data to this article can be found online at https://doi.org/10.1016/j.scitotenv.2026.181869.

## Abbreviations:

BPA: Bisphenol A
BPAF: Bisphenol AF
BPAP: Bisphenol AP
BPB: Bisphenol B
BPF: Bisphenol F
BPP: Bisphenol P
BPS: Bisphenol S
BPZ: Bisphenol Z
CBCL: Child Behavioral Checklist
DEDTP: diethyldithiophosphate
DEP: diethylphosphate
DETP: diethylthiophosphate
DMDTP: dimethyldithiophosphate
DMP: dimethylphosphate
DMTP: dimethylthiophosphate
EDCs: Endocrine Disruptors
ICC: Intra Class correlation
LMM: Linear Mixed Models
LOD: Limit Of Detection
mBP: mono-n-butyl phthalate
mBzP: monobenzyl phthalate
mCHP: mono-cyclohexyl
mCHpP: mono(7-carboxyheptyl)
mCMHP: mono-[(2-carboxymethyl)hexyl]phthalate
mCPP: mono(3-carboxypropyl) phthalate
mECPP: mono-(2-ethyl-5-carboxypentyl) phthalate
mEHHP: mono-(2-ethyl-5-hydroxyhexyl)phthalate
mEOHP: mono-(2-ethyl-5-oxohexyl) phthalate
mEP: mono-ethyl phthalate
mHpP: mono-2-heptylphthalate
mHxP: mono-hexylphthalate
mIBP: mono-isobutyl phthalate
MICE: Multivariate Imputation by Chained Equations
mIDP: mono-hydroxy-isodecyl phthalate
mINP: monoisononylphthalate
mMP: mono-methyl
mOP: monooctylphthalate
PA: phthalic acid
YSR: youth self-report
