# Supplementary material for: Prenatal and childhood exposure to common plasticizers in relation to emotional and behavioral development through adolescence
Source: Sci Total Environ. Author manuscript; Available in PMC 2026 Jul 1. (PMC13320848; doi:10.1016/j.scitotenv.2026.181869)
Supplement: Supplementary Materials [file NIHMS2185941-supplement-Supplementary_Materials.zip › 1-s2.0-S0048969726005334-mmc2.docx]

Supplement for “Prenatal and childhood exposure to common plasticizers in relation to emotional and behavioral development through adolescence”

# Tables

**Table S1. Details on outcome measurement instruments**

| **Instrument** | **Number of items (total)** | | **Number of items (Internalizing problems)** | | **Number of items (Externalizing problems)** |
| --- | --- | --- | --- | --- | --- |
| **Child Behavioral checklist**  Preschool version |  |  | |  | |
| Age 3 years | 99 | 36 (range: -1.0 - 6.2) | | 24 (range: -1.3 – 4.7) | |
| Age 6 years | 99 | 36 (range: -1.0 - 6.2) | | 24 (range: -1.3 – 4.7) | |
| School version |  |  | |  | |
| Age 10 years | 112 | 32 (range: -1.0 – 6.5) | | 35 (range: -0.8 – 6.8) | |
| Age 14 years | 112 | 32 (range: -1.0 – 6.5) | | 35 (range: -0.8 – 6.8) | |
| **Brief problem monitor** |  |  | |  | |
| Age 9 years | 19 | 6 (range: -1.0 – 4.7) | | 7 (range: -1.0 – 4.2) | |
| **Youth-self report** |  |  | |  | |
| Age 14 years | 112 | 31 (range: -1.2 – 4.6) | | 32 (range: -1.3 – 4.5) | |

**Table S2. Sample size per analysis type.**

| **Analysis type** | **Total** | **Boys** | **Girls** |
| --- | --- | --- | --- |
| Prenatal chemical exposure |  |  |  |
| *Child Behavioral checklist* |  |  |  |
| Internalizing problem scores (n) | 1271 | 644 | 627 |
| Externalizing problem scores (n) | 1273 | 644 | 629 |
| *Youth self-report* |  |  |  |
| Internalizing problem scores (n) | 1030 | 515 | 515 |
| Externalizing problem scores (n) | 1031 | 515 | 516 |
|  |  |  |  |
| Childhood BPA or phthalate exposure |  |  |  |
| *Child Behavioral checklist* |  |  |  |
| Internalizing problem scores (n) | 726 | 378 | 348 |
| Externalizing problem scores (n) | 727 | 378 | 349 |
| *Youth self-report* |  |  |  |
| Internalizing problem scores (n) | 596 | 304 | 292 |
| Externalizing problem scores (n) | 596 | 303 | 293 |

**Table S3. Intra Class Correlations for urinary bisphenols, phthalate and organophosphate metabolite concentrations in pregnancy.**

| **ICC bisphenols** | | **ICC phthalates** | | **ICC organophosphates** | |
| --- | --- | --- | --- | --- | --- |
| BPA | 0.01 | mMP | 0.10 | DMP | 0.13 |
| BPS | -0.05 | mEP | 0.16 | DMTP | 0.18 |
| BPZ | - | mCPP | 0.07 | DMDTP | 0.06 |
| BPB | - | mIBP | 0.13 | DEP | 0.04 |
| BPF | 0.10 | mBP | 0.00 | DETP | 0.01 |
| BPAP | - | mECPP | 0.00 | DEDTP | 0.00 |
| BPAF | - | mCMHP | 0.00 |  |  |
| BPP | - | mEHHP | 0.05 |  |  |
|  |  | mBzP | 0.18 |  |  |
|  |  | mINP | - |  |  |
|  |  | mCHP | - |  |  |
|  |  | mOP | - |  |  |
|  |  | PA | 0.07 |  |  |
|  |  | mIDP | - |  |  |
|  |  | mHxP | - |  |  |
|  |  | mHpP | - |  |  |
|  |  | mCHpP | - |  |  |
|  |  | mEOHP | 0.00 |  |  |

*Table note: The Intra Class Correlation (ICC) was computed for the three pregnancy concentrations of every chemical, using a two-way mixed-effects model with a single measurements and absolute agreement. Some cells are empty because for those bisphenols or phthalate metabolites not enough measures were available to calculate the ICC*

**Table S4. Medians and IQRs for urinary organophosphate metabolite concentrations in pregnancy and childhood**

|  |  |  |  |  |  |  |  |  |
| --- | --- | --- | --- | --- | --- | --- | --- | --- |
|  | **< 18 weeks pregnancy** | | **18-25 weeks pregnancy** | | **> 25 weeks pregnancy** | | **Child, age 6 years** | |
|  | Median (IQR) (ng/mL) | %<LOD | Median (IQR) (ng/mL) | %<LOD | Median (IQR) (ng/mL) | %<LOD | Median (IQR) (ng/mL) | %<LOD |
| DMP | 12.2 (6.6 - 22.9) | 0.8 | 12.2 (6.6 - 21.3) | 0.3 | 11.5 (6.1 - 21.2) | 0.1 | 6.7 (3.6 - 12.3) | 0.4 |
| DMTP | 10.9 (4.7 - 21.1) | 4.1 | 11.7 (4.9 - 23.4) | 3.7 | 10.8 (5.1 - 22) | 2.7 | 4.8 (1.9 - 10.4) | 7.3 |
| DMDTP | 0.4 (0.1 - 0.9) | 20.4 | 0.4 (0.1 - 0.9) | 18.2 | 0.3 (0.1 - 0.8) | 18.1 | 0 (0 - 0.1) | 92 |
| DEP | 3.7 (1.8 - 7) | 3.4 | 3.3 (1.6 - 6.4) | 5.5 | 3.5 (1.7 - 7) | 4.2 | 3 (1.5 - 6.3) | 4.3 |
| DETP | 1 (0.4 - 2.4) | 14.2 | 0.8 (0.3 - 2.2) | 14.4 | 0.9 (0.3 - 2.4) | 13.6 | 0.3 (0.1 - 0.9) | 29.9 |
| DEDTP | 0 (0 - 0) | 81.2 | 0 (0 - 0) | 84.6 | 0 (0 - 0) | 84.9 | 0 (0 - 0) | 99.6 |

*Table note: Descriptive statistics were computed using the observed chemical concentrations. For every chemical the median observed concentration in ng/mL together with the Inter Quartile Range (IQR) is reported. Further, the percentage of chemical concentrations below the Limit Of Detection (LOD) is reported. Chemicals with concentrations below the limit of detection (LOD) in 30% or more of the samples were excluded from the analysis.*

DMP: dimethylphosphate; DMTP: dimethylthiophosphate; DMDTP: dimethyldithiophosphate; DEP: diethylphosphate; DETP: diethylthiophosphate; DEDTP: diethyldithiophosphate

**Table S5. Associations of categorical prenatal BPA and phthalate exposure with parent-reported internalizing problem score through age 14 years**

| **Exposure** | **Sample** | **Contrast** | **Beta** | **CI lower limit** | **CI upper limit** | **P value** |
| --- | --- | --- | --- | --- | --- | --- |
| BPA | Total | Mid_vs_Low | -0.06 | -0.23 | 0.10 | 0.438 |
| BPA | Total | High_vs_Low | 0.01 | -0.18 | 0.19 | 0.955 |
| BPA | Total | High_vs_Mid | 0.07 | -0.09 | 0.23 | 0.404 |
| BPA | Boys | Mid_vs_Low | 0.04 | -0.20 | 0.28 | 0.765 |
| BPA | Boys | High_vs_Low | -0.04 | -0.31 | 0.22 | 0.754 |
| BPA | Boys | High_vs_Mid | -0.08 | -0.31 | 0.16 | 0.510 |
| BPA | Girls | Mid_vs_Low | -0.21 | -0.45 | 0.03 | **0.080** |
| BPA | Girls | High_vs_Low | 0.01 | -0.24 | 0.27 | 0.912 |
| BPA | Girls | High_vs_Mid | 0.23 | 0.00 | 0.46 | 0.053 |
| PA | Total | Mid_vs_Low | -0.02 | -0.18 | 0.14 | 0.803 |
| PA | Total | High_vs_Low | -0.05 | -0.22 | 0.12 | 0.577 |
| PA | Total | High_vs_Mid | -0.03 | -0.21 | 0.15 | 0.745 |
| PA | Boys | Mid_vs_Low | 0.16 | -0.06 | 0.37 | 0.149 |
| PA | Boys | High_vs_Low | 0.00 | -0.24 | 0.24 | 0.981 |
| PA | Boys | High_vs_Mid | -0.16 | -0.42 | 0.09 | 0.212 |
| PA | Girls | Mid_vs_Low | -0.17 | -0.41 | 0.07 | 0.164 |
| PA | Girls | High_vs_Low | -0.06 | -0.29 | 0.17 | 0.628 |
| PA | Girls | High_vs_Mid | 0.11 | -0.11 | 0.33 | 0.319 |
| mMP | Total | Mid_vs_Low | 0.24 | 0.08 | 0.40 | **0.003** |
| mMP | Total | High_vs_Low | 0.04 | -0.12 | 0.20 | 0.647 |
| mMP | Total | High_vs_Mid | -0.20 | -0.38 | -0.02 | **0.025** |
| mMP | Boys | Mid_vs_Low | 0.26 | 0.03 | 0.48 | **0.025** |
| mMP | Boys | High_vs_Low | -0.05 | -0.28 | 0.17 | 0.636 |
| mMP | Boys | High_vs_Mid | -0.31 | -0.57 | -0.06 | **0.016** |
| mMP | Girls | Mid_vs_Low | 0.24 | 0.02 | 0.46 | **0.029** |
| mMP | Girls | High_vs_Low | 0.17 | -0.06 | 0.39 | 0.142 |
| mMP | Girls | High_vs_Mid | -0.08 | -0.32 | 0.17 | 0.532 |
| mEP | Total | Mid_vs_Low | 0.00 | -0.16 | 0.16 | 0.991 |
| mEP | Total | High_vs_Low | -0.08 | -0.26 | 0.09 | 0.357 |
| mEP | Total | High_vs_Mid | -0.08 | -0.25 | 0.08 | 0.314 |
| mEP | Boys | Mid_vs_Low | -0.02 | -0.25 | 0.22 | 0.879 |
| mEP | Boys | High_vs_Low | -0.03 | -0.27 | 0.22 | 0.835 |
| mEP | Boys | High_vs_Mid | -0.01 | -0.24 | 0.23 | 0.948 |
| mEP | Girls | Mid_vs_Low | 0.08 | -0.14 | 0.30 | 0.491 |
| mEP | Girls | High_vs_Low | -0.08 | -0.35 | 0.19 | 0.550 |
| mEP | Girls | High_vs_Mid | -0.16 | -0.39 | 0.07 | 0.175 |
| mCPP | Total | Mid_vs_Low | 0.15 | -0.01 | 0.32 | 0.063 |
| mCPP | Total | High_vs_Low | 0.08 | -0.08 | 0.25 | 0.329 |
| mCPP | Total | High_vs_Mid | -0.07 | -0.26 | 0.11 | 0.452 |
| mCPP | Boys | Mid_vs_Low | 0.21 | -0.05 | 0.48 | 0.116 |
| mCPP | Boys | High_vs_Low | 0.14 | -0.07 | 0.35 | 0.197 |
| mCPP | Boys | High_vs_Mid | -0.08 | -0.34 | 0.18 | 0.563 |
| mCPP | Girls | Mid_vs_Low | 0.08 | -0.14 | 0.30 | 0.471 |
| mCPP | Girls | High_vs_Low | 0.00 | -0.27 | 0.26 | 0.988 |
| mCPP | Girls | High_vs_Mid | -0.08 | -0.35 | 0.18 | 0.544 |
| mIBP | Total | Mid_vs_Low | -0.01 | -0.16 | 0.14 | 0.880 |
| mIBP | Total | High_vs_Low | 0.24 | 0.06 | 0.42 | **0.008** |
| mIBP | Total | High_vs_Mid | 0.25 | 0.08 | 0.43 | **0.005** |
| mIBP | Boys | Mid_vs_Low | 0.03 | -0.19 | 0.24 | 0.807 |
| mIBP | Boys | High_vs_Low | 0.27 | 0.04 | 0.50 | **0.021** |
| mIBP | Boys | High_vs_Mid | 0.24 | 0.00 | 0.48 | **0.046** |
| mIBP | Girls | Mid_vs_Low | -0.06 | -0.27 | 0.14 | 0.539 |
| mIBP | Girls | High_vs_Low | 0.24 | -0.03 | 0.50 | 0.077 |
| mIBP | Girls | High_vs_Mid | 0.30 | 0.04 | 0.56 | **0.021** |
| mBP | Total | Mid_vs_Low | 0.00 | -0.16 | 0.16 | 0.997 |
| mBP | Total | High_vs_Low | 0.13 | -0.05 | 0.3 | 0.149 |
| mBP | Total | High_vs_Mid | 0.13 | -0.04 | 0.29 | 0.128 |
| mBP | Boys | Mid_vs_Low | -0.04 | -0.27 | 0.20 | 0.752 |
| mBP | Boys | High_vs_Low | 0.20 | -0.04 | 0.45 | 0.104 |
| mBP | Boys | High_vs_Mid | 0.24 | 0.00 | 0.48 | 0.052 |
| mBP | Girls | Mid_vs_Low | 0.11 | -0.12 | 0.33 | 0.349 |
| mBP | Girls | High_vs_Low | 0.07 | -0.18 | 0.33 | 0.578 |
| mBP | Girls | High_vs_Mid | -0.03 | -0.27 | 0.20 | 0.773 |
| mECPP | Total | Mid_vs_Low | 0.02 | -0.14 | 0.18 | 0.806 |
| mECPP | Total | High_vs_Low | 0.07 | -0.11 | 0.25 | 0.432 |
| mECPP | Total | High_vs_Mid | 0.05 | -0.13 | 0.24 | 0.574 |
| mECPP | Boys | Mid_vs_Low | 0.07 | -0.16 | 0.30 | 0.559 |
| mECPP | Boys | High_vs_Low | 0.12 | -0.14 | 0.37 | 0.362 |
| mECPP | Boys | High_vs_Mid | 0.05 | -0.20 | 0.30 | 0.702 |
| mECPP | Girls | Mid_vs_Low | -0.04 | -0.26 | 0.18 | 0.742 |
| mECPP | Girls | High_vs_Low | 0.02 | -0.24 | 0.28 | 0.878 |
| mECPP | Girls | High_vs_Mid | 0.06 | -0.21 | 0.32 | 0.667 |
| mCMHP | Total | Mid_vs_Low | 0.04 | -0.13 | 0.21 | 0.622 |
| mCMHP | Total | High_vs_Low | -0.03 | -0.21 | 0.15 | 0.727 |
| mCMHP | Total | High_vs_Mid | -0.07 | -0.27 | 0.12 | 0.441 |
| mCMHP | Boys | Mid_vs_Low | 0.05 | -0.19 | 0.30 | 0.678 |
| mCMHP | Boys | High_vs_Low | 0.05 | -0.22 | 0.32 | 0.727 |
| mCMHP | Boys | High_vs_Mid | 0.00 | -0.28 | 0.28 | 0.980 |
| mCMHP | Girls | Mid_vs_Low | 0.08 | -0.17 | 0.33 | 0.531 |
| mCMHP | Girls | High_vs_Low | -0.10 | -0.34 | 0.14 | 0.417 |
| mCMHP | Girls | High_vs_Mid | -0.18 | -0.44 | 0.08 | 0.183 |
| mEHHP | Total | Mid_vs_Low | 0.05 | -0.10 | 0.20 | 0.487 |
| mEHHP | Total | High_vs_Low | 0.17 | -0.01 | 0.36 | 0.065 |
| mEHHP | Total | High_vs_Mid | 0.12 | -0.06 | 0.30 | 0.197 |
| mEHHP | Boys | Mid_vs_Low | -0.04 | -0.25 | 0.17 | 0.717 |
| mEHHP | Boys | High_vs_Low | 0.20 | -0.04 | 0.44 | 0.099 |
| mEHHP | Boys | High_vs_Mid | 0.24 | 0.01 | 0.46 | **0.038** |
| mEHHP | Girls | Mid_vs_Low | 0.18 | -0.03 | 0.38 | 0.097 |
| mEHHP | Girls | High_vs_Low | 0.16 | -0.10 | 0.42 | 0.223 |
| mEHHP | Girls | High_vs_Mid | -0.01 | -0.29 | 0.26 | 0.925 |
| mBzBP | Total | Mid_vs_Low | -0.03 | -0.18 | 0.11 | 0.631 |
| mBzBP | Total | High_vs_Low | 0.14 | -0.05 | 0.33 | 0.160 |
| mBzBP | Total | High_vs_Mid | 0.17 | -0.01 | 0.35 | 0.064 |
| mBzBP | Boys | Mid_vs_Low | -0.04 | -0.26 | 0.17 | 0.697 |
| mBzBP | Boys | High_vs_Low | 0.14 | -0.14 | 0.42 | 0.333 |
| mBzBP | Boys | High_vs_Mid | 0.18 | -0.08 | 0.45 | 0.174 |
| mBzBP | Girls | Mid_vs_Low | -0.01 | -0.22 | 0.19 | 0.894 |
| mBzBP | Girls | High_vs_Low | 0.17 | -0.10 | 0.45 | 0.213 |
| mBzBP | Girls | High_vs_Mid | 0.19 | -0.08 | 0.46 | 0.173 |
| mEOHP | Total | Mid_vs_Low | 0.10 | -0.06 | 0.26 | 0.223 |
| mEOHP | Total | High_vs_Low | 0.16 | -0.01 | 0.34 | 0.066 |
| mEOHP | Total | High_vs_Mid | 0.06 | -0.11 | 0.24 | 0.477 |
| mEOHP | Boys | Mid_vs_Low | 0.06 | -0.17 | 0.29 | 0.612 |
| mEOHP | Boys | High_vs_Low | 0.12 | -0.12 | 0.35 | 0.334 |
| mEOHP | Boys | High_vs_Mid | 0.06 | -0.18 | 0.29 | 0.644 |
| mEOHP | Girls | Mid_vs_Low | 0.16 | -0.05 | 0.38 | 0.137 |
| mEOHP | Girls | High_vs_Low | 0.27 | 0.02 | 0.52 | **0.038** |
| mEOHP | Girls | High_vs_Mid | 0.10 | -0.16 | 0.37 | 0.441 |

**Table S6. Associations of categorical prenatal BPA and phthalate exposure with self-reported internalizing problem score through age 14 years**

| **Exposure** | **Sample** | **Contrast** | **Beta** | **CI lower limit** | **CI upper limit** | **P value** |
| --- | --- | --- | --- | --- | --- | --- |
| BPA | Total | Mid_vs_Low | -0.16 | -0.38 | 0.05 | 0.139 |
| BPA | Total | High_vs_Low | 0.03 | -0.21 | 0.27 | 0.794 |
| BPA | Total | High_vs_Mid | 0.19 | -0.03 | 0.42 | 0.096 |
| BPA | Boys | Mid_vs_Low | -0.04 | -0.28 | 0.21 | 0.775 |
| BPA | Boys | High_vs_Low | 0.04 | -0.24 | 0.31 | 0.789 |
| BPA | Boys | High_vs_Mid | 0.07 | -0.19 | 0.34 | 0.590 |
| BPA | Girls | Mid_vs_Low | -0.36 | -0.70 | -0.03 | 0.031 |
| BPA | Girls | High_vs_Low | -0.09 | -0.50 | 0.31 | 0.652 |
| BPA | Girls | High_vs_Mid | 0.27 | -0.11 | 0.65 | 0.165 |
| PA | Total | Mid_vs_Low | 0.04 | -0.19 | 0.27 | 0.736 |
| PA | Total | High_vs_Low | -0.21 | -0.42 | -0.01 | 0.043 |
| PA | Total | High_vs_Mid | -0.25 | -0.46 | -0.05 | 0.016 |
| PA | Boys | Mid_vs_Low | 0.17 | -0.07 | 0.40 | 0.163 |
| PA | Boys | High_vs_Low | -0.05 | -0.28 | 0.17 | 0.645 |
| PA | Boys | High_vs_Mid | -0.22 | -0.46 | 0.02 | 0.074 |
| PA | Girls | Mid_vs_Low | -0.23 | -0.58 | 0.12 | 0.203 |
| PA | Girls | High_vs_Low | -0.28 | -0.63 | 0.07 | 0.119 |
| PA | Girls | High_vs_Mid | -0.05 | -0.37 | 0.27 | 0.754 |
| mMP | Total | Mid_vs_Low | 0.14 | -0.09 | 0.36 | 0.241 |
| mMP | Total | High_vs_Low | 0.17 | -0.07 | 0.41 | 0.161 |
| mMP | Total | High_vs_Mid | 0.04 | -0.20 | 0.27 | 0.760 |
| mMP | Boys | Mid_vs_Low | -0.05 | -0.34 | 0.24 | 0.736 |
| mMP | Boys | High_vs_Low | -0.02 | -0.30 | 0.26 | 0.897 |
| mMP | Boys | High_vs_Mid | 0.03 | -0.18 | 0.24 | 0.771 |
| mMP | Girls | Mid_vs_Low | 0.30 | -0.02 | 0.62 | 0.065 |
| mMP | Girls | High_vs_Low | 0.33 | -0.06 | 0.72 | 0.101 |
| mMP | Girls | High_vs_Mid | 0.03 | -0.40 | 0.45 | 0.906 |
| mEP | Total | Mid_vs_Low | 0.13 | -0.09 | 0.35 | 0.249 |
| mEP | Total | High_vs_Low | -0.03 | -0.26 | 0.19 | 0.778 |
| mEP | Total | High_vs_Mid | -0.16 | -0.39 | 0.07 | 0.174 |
| mEP | Boys | Mid_vs_Low | 0.06 | -0.19 | 0.30 | 0.649 |
| mEP | Boys | High_vs_Low | 0.04 | -0.21 | 0.30 | 0.740 |
| mEP | Boys | High_vs_Mid | -0.01 | -0.26 | 0.23 | 0.913 |
| mEP | Girls | Mid_vs_Low | 0.28 | -0.06 | 0.61 | 0.108 |
| mEP | Girls | High_vs_Low | -0.14 | -0.51 | 0.23 | 0.459 |
| mEP | Girls | High_vs_Mid | -0.41 | -0.79 | -0.04 | 0.030 |
| mCPP | Total | Mid_vs_Low | 0.01 | -0.22 | 0.23 | 0.961 |
| mCPP | Total | High_vs_Low | 0.06 | -0.18 | 0.30 | 0.619 |
| mCPP | Total | High_vs_Mid | 0.05 | -0.20 | 0.30 | 0.669 |
| mCPP | Boys | Mid_vs_Low | -0.20 | -0.48 | 0.08 | 0.161 |
| mCPP | Boys | High_vs_Low | -0.01 | -0.27 | 0.26 | 0.962 |
| mCPP | Boys | High_vs_Mid | 0.19 | -0.05 | 0.44 | 0.117 |
| mCPP | Girls | Mid_vs_Low | 0.08 | -0.28 | 0.43 | 0.673 |
| mCPP | Girls | High_vs_Low | 0.13 | -0.24 | 0.49 | 0.487 |
| mCPP | Girls | High_vs_Mid | 0.05 | -0.36 | 0.47 | 0.805 |
| mIBP | Total | Mid_vs_Low | 0.02 | -0.17 | 0.21 | 0.801 |
| mIBP | Total | High_vs_Low | 0.20 | -0.04 | 0.45 | 0.106 |
| mIBP | Total | High_vs_Mid | 0.18 | -0.07 | 0.42 | 0.153 |
| mIBP | Boys | Mid_vs_Low | 0.01 | -0.23 | 0.25 | 0.939 |
| mIBP | Boys | High_vs_Low | 0.07 | -0.18 | 0.33 | 0.567 |
| mIBP | Boys | High_vs_Mid | 0.06 | -0.19 | 0.32 | 0.626 |
| mIBP | Girls | Mid_vs_Low | 0.05 | -0.26 | 0.36 | 0.742 |
| mIBP | Girls | High_vs_Low | 0.34 | -0.04 | 0.71 | 0.076 |
| mIBP | Girls | High_vs_Mid | 0.29 | -0.14 | 0.72 | 0.191 |
| mBP | Total | Mid_vs_Low | 0.25 | 0.04 | 0.47 | 0.019 |
| mBP | Total | High_vs_Low | 0.17 | -0.05 | 0.39 | 0.137 |
| mBP | Total | High_vs_Mid | -0.08 | -0.31 | 0.14 | 0.468 |
| mBP | Boys | Mid_vs_Low | 0.07 | -0.20 | 0.33 | 0.615 |
| mBP | Boys | High_vs_Low | -0.06 | -0.33 | 0.20 | 0.641 |
| mBP | Boys | High_vs_Mid | -0.13 | -0.38 | 0.11 | 0.294 |
| mBP | Girls | Mid_vs_Low | 0.38 | 0.06 | 0.71 | 0.021 |
| mBP | Girls | High_vs_Low | 0.30 | -0.02 | 0.62 | 0.066 |
| mBP | Girls | High_vs_Mid | -0.08 | -0.46 | 0.29 | 0.668 |
| mECPP | Total | Mid_vs_Low | 0.00 | -0.23 | 0.24 | 0.968 |
| mECPP | Total | High_vs_Low | -0.02 | -0.24 | 0.20 | 0.851 |
| mECPP | Total | High_vs_Mid | -0.03 | -0.24 | 0.19 | 0.811 |
| mECPP | Boys | Mid_vs_Low | 0.00 | -0.24 | 0.25 | 0.972 |
| mECPP | Boys | High_vs_Low | -0.22 | -0.47 | 0.03 | 0.090 |
| mECPP | Boys | High_vs_Mid | -0.22 | -0.47 | 0.03 | 0.079 |
| mECPP | Girls | Mid_vs_Low | 0.03 | -0.33 | 0.38 | 0.887 |
| mECPP | Girls | High_vs_Low | 0.26 | -0.07 | 0.60 | 0.120 |
| mECPP | Girls | High_vs_Mid | 0.24 | -0.09 | 0.57 | 0.157 |
| mCMHP | Total | Mid_vs_Low | 0.00 | -0.24 | 0.24 | 0.998 |
| mCMHP | Total | High_vs_Low | -0.09 | -0.32 | 0.13 | 0.421 |
| mCMHP | Total | High_vs_Mid | -0.09 | -0.32 | 0.13 | 0.423 |
| mCMHP | Boys | Mid_vs_Low | -0.15 | -0.41 | 0.11 | 0.255 |
| mCMHP | Boys | High_vs_Low | -0.12 | -0.38 | 0.15 | 0.388 |
| mCMHP | Boys | High_vs_Mid | 0.03 | -0.20 | 0.27 | 0.781 |
| mCMHP | Girls | Mid_vs_Low | 0.07 | -0.34 | 0.47 | 0.750 |
| mCMHP | Girls | High_vs_Low | -0.10 | -0.45 | 0.26 | 0.589 |
| mCMHP | Girls | High_vs_Mid | -0.16 | -0.56 | 0.23 | 0.414 |
| mEHHP | Total | Mid_vs_Low | -0.05 | -0.28 | 0.18 | 0.668 |
| mEHHP | Total | High_vs_Low | 0.07 | -0.15 | 0.28 | 0.553 |
| mEHHP | Total | High_vs_Mid | 0.12 | -0.10 | 0.33 | 0.285 |
| mEHHP | Boys | Mid_vs_Low | -0.38 | -0.60 | -0.16 | 0.001 |
| mEHHP | Boys | High_vs_Low | -0.12 | -0.36 | 0.13 | 0.360 |
| mEHHP | Boys | High_vs_Mid | 0.27 | 0.06 | 0.48 | 0.012 |
| mEHHP | Girls | Mid_vs_Low | 0.18 | -0.16 | 0.51 | 0.307 |
| mEHHP | Girls | High_vs_Low | 0.23 | -0.09 | 0.55 | 0.161 |
| mEHHP | Girls | High_vs_Mid | 0.05 | -0.31 | 0.42 | 0.768 |
| mBzBP | Total | Mid_vs_Low | -0.05 | -0.26 | 0.16 | 0.642 |
| mBzBP | Total | High_vs_Low | 0.11 | -0.15 | 0.37 | 0.406 |
| mBzBP | Total | High_vs_Mid | 0.16 | -0.09 | 0.41 | 0.201 |
| mBzBP | Boys | Mid_vs_Low | 0.07 | -0.17 | 0.31 | 0.573 |
| mBzBP | Boys | High_vs_Low | 0.04 | -0.21 | 0.29 | 0.739 |
| mBzBP | Boys | High_vs_Mid | -0.03 | -0.28 | 0.23 | 0.837 |
| mBzBP | Girls | Mid_vs_Low | -0.28 | -0.61 | 0.06 | 0.104 |
| mBzBP | Girls | High_vs_Low | 0.09 | -0.36 | 0.55 | 0.684 |
| mBzBP | Girls | High_vs_Mid | 0.37 | -0.03 | 0.78 | 0.070 |
| mEOHP | Total | Mid_vs_Low | 0.06 | -0.16 | 0.27 | 0.619 |
| mEOHP | Total | High_vs_Low | 0.10 | -0.13 | 0.33 | 0.388 |
| mEOHP | Total | High_vs_Mid | 0.05 | -0.19 | 0.28 | 0.709 |
| mEOHP | Boys | Mid_vs_Low | -0.26 | -0.51 | -0.01 | 0.039 |
| mEOHP | Boys | High_vs_Low | -0.17 | -0.43 | 0.09 | 0.202 |
| mEOHP | Boys | High_vs_Mid | 0.09 | -0.13 | 0.30 | 0.415 |
| mEOHP | Girls | Mid_vs_Low | 0.26 | -0.06 | 0.57 | 0.108 |
| mEOHP | Girls | High_vs_Low | 0.31 | -0.05 | 0.67 | 0.089 |
| mEOHP | Girls | High_vs_Mid | 0.05 | -0.37 | 0.48 | 0.807 |

**Table S7. Associations of categorical prenatal BPA and phthalate exposure with parent-reported externalizing problem score through age 14 years**

| **Exposure** | **Sample** | **Contrast** | **Beta** | **CI lower limit** | **CI upper limit** | **P value** |
| --- | --- | --- | --- | --- | --- | --- |
| BPA | Total | Mid_vs_Low | -0.09 | -0.29 | 0.12 | 0.400 |
| BPA | Total | High_vs_Low | -0.13 | -0.33 | 0.08 | 0.215 |
| BPA | Total | High_vs_Mid | -0.04 | -0.21 | 0.13 | 0.624 |
| BPA | Boys | Mid_vs_Low | -0.02 | -0.31 | 0.27 | 0.881 |
| BPA | Boys | High_vs_Low | -0.16 | -0.44 | 0.11 | 0.242 |
| BPA | Boys | High_vs_Mid | -0.14 | -0.40 | 0.11 | 0.275 |
| BPA | Girls | Mid_vs_Low | -0.21 | -0.49 | 0.07 | 0.142 |
| BPA | Girls | High_vs_Low | -0.05 | -0.34 | 0.24 | 0.753 |
| BPA | Girls | High_vs_Mid | 0.16 | -0.06 | 0.39 | 0.154 |
| PA | Total | Mid_vs_Low | -0.01 | -0.21 | 0.18 | 0.898 |
| PA | Total | High_vs_Low | -0.18 | -0.39 | 0.03 | 0.087 |
| PA | Total | High_vs_Mid | -0.17 | -0.35 | 0.01 | 0.066 |
| PA | Boys | Mid_vs_Low | 0.14 | -0.16 | 0.43 | 0.372 |
| PA | Boys | High_vs_Low | -0.23 | -0.54 | 0.07 | 0.135 |
| PA | Boys | High_vs_Mid | -0.37 | -0.65 | -0.09 | **0.010** |
| PA | Girls | Mid_vs_Low | -0.11 | -0.39 | 0.17 | 0.426 |
| PA | Girls | High_vs_Low | -0.08 | -0.33 | 0.17 | 0.543 |
| PA | Girls | High_vs_Mid | 0.04 | -0.18 | 0.26 | 0.753 |
| mMP | Total | Mid_vs_Low | 0.15 | -0.04 | 0.33 | 0.128 |
| mMP | Total | High_vs_Low | 0.01 | -0.19 | 0.21 | 0.918 |
| mMP | Total | High_vs_Mid | -0.14 | -0.33 | 0.06 | 0.173 |
| mMP | Boys | Mid_vs_Low | 0.12 | -0.18 | 0.42 | 0.427 |
| mMP | Boys | High_vs_Low | -0.09 | -0.37 | 0.20 | 0.544 |
| mMP | Boys | High_vs_Mid | -0.21 | -0.48 | 0.07 | 0.141 |
| mMP | Girls | Mid_vs_Low | 0.25 | 0.03 | 0.48 | **0.029** |
| mMP | Girls | High_vs_Low | 0.17 | -0.09 | 0.44 | 0.203 |
| mMP | Girls | High_vs_Mid | -0.08 | -0.34 | 0.18 | 0.546 |
| mEP | Total | Mid_vs_Low | -0.05 | -0.24 | 0.14 | 0.631 |
| mEP | Total | High_vs_Low | -0.13 | -0.32 | 0.07 | 0.207 |
| mEP | Total | High_vs_Mid | -0.08 | -0.26 | 0.10 | 0.378 |
| mEP | Boys | Mid_vs_Low | 0.02 | -0.26 | 0.29 | 0.911 |
| mEP | Boys | High_vs_Low | -0.01 | -0.30 | 0.28 | 0.932 |
| mEP | Boys | High_vs_Mid | -0.03 | -0.31 | 0.25 | 0.841 |
| mEP | Girls | Mid_vs_Low | -0.07 | -0.32 | 0.17 | 0.567 |
| mEP | Girls | High_vs_Low | -0.23 | -0.49 | 0.03 | 0.089 |
| mEP | Girls | High_vs_Mid | -0.16 | -0.36 | 0.05 | 0.139 |
| mCPP | Total | Mid_vs_Low | 0.02 | -0.17 | 0.21 | 0.811 |
| mCPP | Total | High_vs_Low | -0.13 | -0.33 | 0.06 | 0.190 |
| mCPP | Total | High_vs_Mid | -0.15 | -0.35 | 0.05 | 0.131 |
| mCPP | Boys | Mid_vs_Low | 0.00 | -0.29 | 0.29 | 0.993 |
| mCPP | Boys | High_vs_Low | -0.11 | -0.40 | 0.18 | 0.447 |
| mCPP | Boys | High_vs_Mid | -0.11 | -0.41 | 0.18 | 0.451 |
| mCPP | Girls | Mid_vs_Low | -0.01 | -0.25 | 0.23 | 0.923 |
| mCPP | Girls | High_vs_Low | -0.20 | -0.46 | 0.06 | 0.124 |
| mCPP | Girls | High_vs_Mid | -0.19 | -0.44 | 0.06 | 0.141 |
| mIBP | Total | Mid_vs_Low | 0.02 | -0.17 | 0.22 | 0.804 |
| mIBP | Total | High_vs_Low | 0.12 | -0.07 | 0.32 | 0.217 |
| mIBP | Total | High_vs_Mid | 0.10 | -0.10 | 0.30 | 0.336 |
| mIBP | Boys | Mid_vs_Low | 0.15 | -0.15 | 0.44 | 0.328 |
| mIBP | Boys | High_vs_Low | 0.14 | -0.12 | 0.40 | 0.300 |
| mIBP | Boys | High_vs_Mid | -0.01 | -0.30 | 0.28 | 0.963 |
| mIBP | Girls | Mid_vs_Low | -0.11 | -0.35 | 0.13 | 0.364 |
| mIBP | Girls | High_vs_Low | 0.12 | -0.14 | 0.38 | 0.374 |
| mIBP | Girls | High_vs_Mid | 0.23 | -0.04 | 0.50 | 0.092 |
| mBP | Total | Mid_vs_Low | -0.01 | -0.19 | 0.18 | 0.941 |
| mBP | Total | High_vs_Low | 0.05 | -0.13 | 0.24 | 0.582 |
| mBP | Total | High_vs_Mid | 0.06 | -0.13 | 0.25 | 0.540 |
| mBP | Boys | Mid_vs_Low | -0.19 | -0.49 | 0.12 | 0.230 |
| mBP | Boys | High_vs_Low | 0.08 | -0.21 | 0.36 | 0.588 |
| mBP | Boys | High_vs_Mid | 0.26 | -0.03 | 0.56 | 0.075 |
| mBP | Girls | Mid_vs_Low | 0.26 | 0.02 | 0.49 | **0.031** |
| mBP | Girls | High_vs_Low | 0.06 | -0.18 | 0.30 | 0.653 |
| mBP | Girls | High_vs_Mid | -0.20 | -0.46 | 0.06 | 0.126 |
| mECPP | Total | Mid_vs_Low | -0.02 | -0.21 | 0.18 | 0.857 |
| mECPP | Total | High_vs_Low | 0.07 | -0.13 | 0.28 | 0.468 |
| mECPP | Total | High_vs_Mid | 0.09 | -0.10 | 0.28 | 0.344 |
| mECPP | Boys | Mid_vs_Low | 0.05 | -0.23 | 0.33 | 0.743 |
| mECPP | Boys | High_vs_Low | 0.24 | -0.08 | 0.56 | 0.141 |
| mECPP | Boys | High_vs_Mid | 0.19 | -0.11 | 0.50 | 0.216 |
| mECPP | Girls | Mid_vs_Low | -0.11 | -0.37 | 0.16 | 0.433 |
| mECPP | Girls | High_vs_Low | -0.09 | -0.35 | 0.16 | 0.479 |
| mECPP | Girls | High_vs_Mid | 0.01 | -0.22 | 0.25 | 0.911 |
| mCMHP | Total | Mid_vs_Low | 0.10 | -0.08 | 0.29 | 0.280 |
| mCMHP | Total | High_vs_Low | 0.12 | -0.09 | 0.33 | 0.271 |
| mCMHP | Total | High_vs_Mid | 0.01 | -0.21 | 0.24 | 0.895 |
| mCMHP | Boys | Mid_vs_Low | 0.11 | -0.14 | 0.36 | 0.391 |
| mCMHP | Boys | High_vs_Low | 0.28 | -0.07 | 0.64 | 0.119 |
| mCMHP | Boys | High_vs_Mid | 0.17 | -0.18 | 0.53 | 0.339 |
| mCMHP | Girls | Mid_vs_Low | 0.10 | -0.17 | 0.38 | 0.457 |
| mCMHP | Girls | High_vs_Low | 0.03 | -0.20 | 0.27 | 0.769 |
| mCMHP | Girls | High_vs_Mid | -0.07 | -0.36 | 0.22 | 0.635 |
| mEHHP | Total | Mid_vs_Low | 0.05 | -0.13 | 0.24 | 0.583 |
| mEHHP | Total | High_vs_Low | 0.15 | -0.05 | 0.34 | 0.138 |
| mEHHP | Total | High_vs_Mid | 0.10 | -0.12 | 0.31 | 0.374 |
| mEHHP | Boys | Mid_vs_Low | 0.01 | -0.27 | 0.28 | 0.952 |
| mEHHP | Boys | High_vs_Low | 0.20 | -0.09 | 0.50 | 0.177 |
| mEHHP | Boys | High_vs_Mid | 0.20 | -0.10 | 0.49 | 0.195 |
| mEHHP | Girls | Mid_vs_Low | 0.15 | -0.09 | 0.39 | 0.215 |
| mEHHP | Girls | High_vs_Low | 0.15 | -0.10 | 0.40 | 0.253 |
| mEHHP | Girls | High_vs_Mid | -0.01 | -0.29 | 0.28 | 0.962 |
| mBzBP | Total | Mid_vs_Low | -0.11 | -0.28 | 0.06 | 0.221 |
| mBzBP | Total | High_vs_Low | 0.07 | -0.14 | 0.28 | 0.500 |
| mBzBP | Total | High_vs_Mid | 0.18 | -0.03 | 0.38 | 0.092 |
| mBzBP | Boys | Mid_vs_Low | -0.11 | -0.40 | 0.17 | 0.427 |
| mBzBP | Boys | High_vs_Low | 0.05 | -0.27 | 0.36 | 0.771 |
| mBzBP | Boys | High_vs_Mid | 0.16 | -0.15 | 0.47 | 0.304 |
| mBzBP | Girls | Mid_vs_Low | -0.03 | -0.25 | 0.18 | 0.758 |
| mBzBP | Girls | High_vs_Low | 0.15 | -0.13 | 0.43 | 0.308 |
| mBzBP | Girls | High_vs_Mid | 0.18 | -0.10 | 0.46 | 0.211 |
| mEOHP | Total | Mid_vs_Low | 0.15 | -0.03 | 0.33 | 0.107 |
| mEOHP | Total | High_vs_Low | 0.10 | -0.10 | 0.31 | 0.325 |
| mEOHP | Total | High_vs_Mid | -0.05 | -0.25 | 0.16 | 0.659 |
| mEOHP | Boys | Mid_vs_Low | 0.23 | -0.05 | 0.51 | 0.101 |
| mEOHP | Boys | High_vs_Low | 0.07 | -0.22 | 0.37 | 0.627 |
| mEOHP | Boys | High_vs_Mid | -0.16 | -0.44 | 0.13 | 0.275 |
| mEOHP | Girls | Mid_vs_Low | 0.09 | -0.12 | 0.31 | 0.398 |
| mEOHP | Girls | High_vs_Low | 0.22 | -0.05 | 0.49 | 0.113 |
| mEOHP | Girls | High_vs_Mid | 0.12 | -0.17 | 0.42 | 0.406 |

**Table S8. Associations of categorical prenatal BPA and phthalate exposure with self-reported externalizing problem score through age 14 years**

| **Exposure** | **Sample** | **Contrast** | **Beta** | **CI lower limit** | **CI upper limit** | **P value** |
| --- | --- | --- | --- | --- | --- | --- |
| BPA | Total | Mid_vs_Low | 0.05 | -0.17 | 0.26 | 0.672 |
| BPA | Total | High_vs_Low | 0.10 | -0.12 | 0.33 | 0.356 |
| BPA | Total | High_vs_Mid | 0.06 | -0.14 | 0.26 | 0.574 |
| BPA | Boys | Mid_vs_Low | 0.24 | -0.09 | 0.58 | 0.155 |
| BPA | Boys | High_vs_Low | 0.11 | -0.21 | 0.42 | 0.498 |
| BPA | Boys | High_vs_Mid | -0.13 | -0.43 | 0.16 | 0.374 |
| BPA | Girls | Mid_vs_Low | -0.15 | -0.44 | 0.13 | 0.287 |
| BPA | Girls | High_vs_Low | 0.05 | -0.27 | 0.38 | 0.755 |
| BPA | Girls | High_vs_Mid | 0.21 | -0.08 | 0.50 | 0.164 |
| PA | Total | Mid_vs_Low | 0.06 | -0.17 | 0.29 | 0.613 |
| PA | Total | High_vs_Low | -0.18 | -0.39 | 0.02 | 0.084 |
| PA | Total | High_vs_Mid | -0.24 | -0.44 | -0.04 | **0.019** |
| PA | Boys | Mid_vs_Low | 0.14 | -0.18 | 0.47 | 0.382 |
| PA | Boys | High_vs_Low | -0.25 | -0.55 | 0.06 | 0.112 |
| PA | Boys | High_vs_Mid | -0.39 | -0.66 | -0.12 | **0.004** |
| PA | Girls | Mid_vs_Low | -0.23 | -0.54 | 0.07 | 0.135 |
| PA | Girls | High_vs_Low | -0.02 | -0.29 | 0.25 | 0.880 |
| PA | Girls | High_vs_Mid | 0.21 | -0.04 | 0.47 | 0.103 |
| mMP | Total | Mid_vs_Low | 0.22 | 0.00 | 0.44 | 0.051 |
| mMP | Total | High_vs_Low | 0.13 | -0.10 | 0.35 | 0.262 |
| mMP | Total | High_vs_Mid | -0.09 | -0.29 | 0.11 | 0.375 |
| mMP | Boys | Mid_vs_Low | 0.32 | -0.01 | 0.65 | 0.054 |
| mMP | Boys | High_vs_Low | 0.11 | -0.15 | 0.37 | 0.410 |
| mMP | Boys | High_vs_Mid | -0.21 | -0.45 | 0.03 | 0.086 |
| mMP | Girls | Mid_vs_Low | 0.25 | -0.03 | 0.54 | 0.085 |
| mMP | Girls | High_vs_Low | 0.29 | -0.04 | 0.62 | 0.088 |
| mMP | Girls | High_vs_Mid | 0.03 | -0.27 | 0.34 | 0.826 |
| mEP | Total | Mid_vs_Low | 0.11 | -0.09 | 0.32 | 0.270 |
| mEP | Total | High_vs_Low | -0.04 | -0.24 | 0.16 | 0.717 |
| mEP | Total | High_vs_Mid | -0.15 | -0.38 | 0.07 | 0.189 |
| mEP | Boys | Mid_vs_Low | 0.09 | -0.18 | 0.36 | 0.518 |
| mEP | Boys | High_vs_Low | 0.08 | -0.21 | 0.38 | 0.568 |
| mEP | Boys | High_vs_Mid | 0.00 | -0.29 | 0.29 | 0.973 |
| mEP | Girls | Mid_vs_Low | 0.15 | -0.15 | 0.44 | 0.330 |
| mEP | Girls | High_vs_Low | -0.15 | -0.44 | 0.13 | 0.291 |
| mEP | Girls | High_vs_Mid | -0.30 | -0.61 | 0.01 | 0.056 |
| mCPP | Total | Mid_vs_Low | 0.07 | -0.14 | 0.28 | 0.523 |
| mCPP | Total | High_vs_Low | -0.10 | -0.31 | 0.11 | 0.358 |
| mCPP | Total | High_vs_Mid | -0.17 | -0.38 | 0.04 | 0.121 |
| mCPP | Boys | Mid_vs_Low | -0.06 | -0.35 | 0.22 | 0.661 |
| mCPP | Boys | High_vs_Low | -0.11 | -0.45 | 0.23 | 0.529 |
| mCPP | Boys | High_vs_Mid | -0.04 | -0.33 | 0.24 | 0.760 |
| mCPP | Girls | Mid_vs_Low | 0.03 | -0.25 | 0.32 | 0.819 |
| mCPP | Girls | High_vs_Low | -0.11 | -0.39 | 0.17 | 0.436 |
| mCPP | Girls | High_vs_Mid | -0.15 | -0.46 | 0.17 | 0.361 |
| mIBP | Total | Mid_vs_Low | 0.01 | -0.19 | 0.22 | 0.905 |
| mIBP | Total | High_vs_Low | 0.21 | -0.02 | 0.44 | 0.073 |
| mIBP | Total | High_vs_Mid | 0.20 | -0.01 | 0.40 | 0.060 |
| mIBP | Boys | Mid_vs_Low | 0.22 | -0.08 | 0.53 | 0.154 |
| mIBP | Boys | High_vs_Low | 0.19 | -0.10 | 0.48 | 0.192 |
| mIBP | Boys | High_vs_Mid | -0.03 | -0.32 | 0.26 | 0.841 |
| mIBP | Girls | Mid_vs_Low | -0.15 | -0.42 | 0.13 | 0.291 |
| mIBP | Girls | High_vs_Low | 0.21 | -0.13 | 0.54 | 0.225 |
| mIBP | Girls | High_vs_Mid | 0.35 | 0.04 | 0.67 | **0.028** |
| mBP | Total | Mid_vs_Low | 0.24 | 0.02 | 0.46 | **0.032** |
| mBP | Total | High_vs_Low | 0.17 | -0.03 | 0.37 | 0.104 |
| mBP | Total | High_vs_Mid | -0.07 | -0.30 | 0.15 | 0.526 |
| mBP | Boys | Mid_vs_Low | 0.08 | -0.23 | 0.39 | 0.614 |
| mBP | Boys | High_vs_Low | 0.21 | -0.11 | 0.52 | 0.198 |
| mBP | Boys | High_vs_Mid | 0.13 | -0.19 | 0.45 | 0.432 |
| mBP | Girls | Mid_vs_Low | 0.39 | 0.07 | 0.72 | **0.017** |
| mBP | Girls | High_vs_Low | 0.05 | -0.23 | 0.32 | 0.741 |
| mBP | Girls | High_vs_Mid | -0.35 | -0.67 | -0.03 | **0.033** |
| mECPP | Total | Mid_vs_Low | 0.07 | -0.15 | 0.29 | 0.537 |
| mECPP | Total | High_vs_Low | 0.08 | -0.12 | 0.28 | 0.415 |
| mECPP | Total | High_vs_Mid | 0.01 | -0.20 | 0.23 | 0.907 |
| mECPP | Boys | Mid_vs_Low | 0.15 | -0.15 | 0.45 | 0.339 |
| mECPP | Boys | High_vs_Low | 0.29 | 0.01 | 0.56 | **0.041** |
| mECPP | Boys | High_vs_Mid | 0.14 | -0.21 | 0.48 | 0.430 |
| mECPP | Girls | Mid_vs_Low | 0.05 | -0.28 | 0.37 | 0.769 |
| mECPP | Girls | High_vs_Low | -0.03 | -0.31 | 0.26 | 0.853 |
| mECPP | Girls | High_vs_Mid | -0.08 | -0.35 | 0.20 | 0.589 |
| mCMHP | Total | Mid_vs_Low | 0.15 | -0.07 | 0.37 | 0.189 |
| mCMHP | Total | High_vs_Low | 0.05 | -0.14 | 0.25 | 0.592 |
| mCMHP | Total | High_vs_Mid | -0.10 | -0.32 | 0.13 | 0.412 |
| mCMHP | Boys | Mid_vs_Low | 0.10 | -0.18 | 0.38 | 0.471 |
| mCMHP | Boys | High_vs_Low | 0.32 | 0.02 | 0.62 | **0.034** |
| mCMHP | Boys | High_vs_Mid | 0.22 | -0.09 | 0.53 | 0.171 |
| mCMHP | Girls | Mid_vs_Low | 0.20 | -0.17 | 0.57 | 0.296 |
| mCMHP | Girls | High_vs_Low | -0.05 | -0.31 | 0.21 | 0.707 |
| mCMHP | Girls | High_vs_Mid | -0.25 | -0.58 | 0.08 | 0.141 |
| mEHHP | Total | Mid_vs_Low | -0.02 | -0.24 | 0.20 | 0.870 |
| mEHHP | Total | High_vs_Low | -0.06 | -0.26 | 0.14 | 0.573 |
| mEHHP | Total | High_vs_Mid | -0.04 | -0.26 | 0.18 | 0.726 |
| mEHHP | Boys | Mid_vs_Low | -0.03 | -0.36 | 0.30 | 0.869 |
| mEHHP | Boys | High_vs_Low | 0.04 | -0.25 | 0.32 | 0.797 |
| mEHHP | Boys | High_vs_Mid | 0.07 | -0.24 | 0.37 | 0.675 |
| mEHHP | Girls | Mid_vs_Low | 0.09 | -0.23 | 0.40 | 0.591 |
| mEHHP | Girls | High_vs_Low | -0.08 | -0.35 | 0.20 | 0.582 |
| mEHHP | Girls | High_vs_Mid | -0.16 | -0.46 | 0.13 | 0.274 |
| mBzBP | Total | Mid_vs_Low | 0.02 | -0.2 | 0.24 | 0.870 |
| mBzBP | Total | High_vs_Low | -0.05 | -0.26 | 0.16 | 0.648 |
| mBzBP | Total | High_vs_Mid | -0.07 | -0.29 | 0.15 | 0.551 |
| mBzBP | Boys | Mid_vs_Low | 0.19 | -0.11 | 0.50 | 0.211 |
| mBzBP | Boys | High_vs_Low | 0.14 | -0.11 | 0.38 | 0.268 |
| mBzBP | Boys | High_vs_Mid | -0.06 | -0.34 | 0.23 | 0.701 |
| mBzBP | Girls | Mid_vs_Low | -0.11 | -0.40 | 0.17 | 0.439 |
| mBzBP | Girls | High_vs_Low | -0.23 | -0.55 | 0.09 | 0.164 |
| mBzBP | Girls | High_vs_Mid | -0.12 | -0.45 | 0.22 | 0.499 |
| mEOHP | Total | Mid_vs_Low | 0.10 | -0.12 | 0.32 | 0.365 |
| mEOHP | Total | High_vs_Low | 0.06 | -0.15 | 0.27 | 0.574 |
| mEOHP | Total | High_vs_Mid | -0.04 | -0.26 | 0.18 | 0.704 |
| mEOHP | Boys | Mid_vs_Low | 0.03 | -0.31 | 0.36 | 0.877 |
| mEOHP | Boys | High_vs_Low | 0.06 | -0.24 | 0.35 | 0.709 |
| mEOHP | Boys | High_vs_Mid | 0.03 | -0.28 | 0.34 | 0.853 |
| mEOHP | Girls | Mid_vs_Low | 0.22 | -0.06 | 0.49 | 0.121 |
| mEOHP | Girls | High_vs_Low | 0.10 | -0.21 | 0.40 | 0.529 |
| mEOHP | Girls | High_vs_Mid | -0.12 | -0.45 | 0.21 | 0.477 |

**Table S9. Associations of categorical childhood BPA and phthalate exposure with parent-reported internalizing problem score through age 14 years**

| **Exposure** | **Sample** | **Contrast** | **Beta** | **CI lower limit** | **CI upper limit** | **P value** |
| --- | --- | --- | --- | --- | --- | --- |
| BPA | Total | Mid_vs_Low | -0.04 | -0.28 | 0.19 | 0.710 |
| BPA | Total | High_vs_Low | -0.22 | -0.46 | 0.01 | 0.065 |
| BPA | Total | High_vs_Mid | -0.18 | -0.40 | 0.05 | 0.121 |
| BPA | Boys | Mid_vs_Low | -0.31 | -0.58 | -0.03 | 0.029 |
| BPA | Boys | High_vs_Low | -0.47 | -0.78 | -0.16 | **0.003** |
| BPA | Boys | High_vs_Mid | -0.16 | -0.45 | 0.13 | 0.277 |
| BPA | Girls | Mid_vs_Low | 0.15 | -0.25 | 0.54 | 0.464 |
| BPA | Girls | High_vs_Low | -0.10 | -0.46 | 0.25 | 0.566 |
| BPA | Girls | High_vs_Mid | -0.25 | -0.63 | 0.13 | 0.193 |
| PA | Total | Mid_vs_Low | 0.00 | -0.19 | 0.19 | 0.998 |
| PA | Total | High_vs_Low | 0.08 | -0.14 | 0.29 | 0.490 |
| PA | Total | High_vs_Mid | 0.08 | -0.13 | 0.28 | 0.470 |
| PA | Boys | Mid_vs_Low | -0.16 | -0.42 | 0.10 | 0.221 |
| PA | Boys | High_vs_Low | -0.02 | -0.31 | 0.28 | 0.914 |
| PA | Boys | High_vs_Mid | 0.15 | -0.15 | 0.44 | 0.330 |
| PA | Girls | Mid_vs_Low | 0.16 | -0.11 | 0.42 | 0.254 |
| PA | Girls | High_vs_Low | 0.07 | -0.23 | 0.38 | 0.633 |
| PA | Girls | High_vs_Mid | -0.08 | -0.38 | 0.21 | 0.581 |
| mMP | Total | Mid_vs_Low | -0.02 | -0.26 | 0.22 | 0.891 |
| mMP | Total | High_vs_Low | -0.08 | -0.34 | 0.18 | 0.531 |
| mMP | Total | High_vs_Mid | -0.07 | -0.30 | 0.16 | 0.573 |
| mMP | Boys | Mid_vs_Low | -0.12 | -0.42 | 0.18 | 0.425 |
| mMP | Boys | High_vs_Low | -0.10 | -0.47 | 0.26 | 0.575 |
| mMP | Boys | High_vs_Mid | 0.02 | -0.32 | 0.35 | 0.925 |
| mMP | Girls | Mid_vs_Low | -0.02 | -0.41 | 0.38 | 0.931 |
| mMP | Girls | High_vs_Low | -0.07 | -0.45 | 0.31 | 0.707 |
| mMP | Girls | High_vs_Mid | -0.06 | -0.39 | 0.28 | 0.749 |
| mEP | Total | Mid_vs_Low | 0.03 | -0.19 | 0.24 | 0.796 |
| mEP | Total | High_vs_Low | -0.14 | -0.34 | 0.05 | 0.155 |
| mEP | Total | High_vs_Mid | -0.17 | -0.39 | 0.05 | 0.127 |
| mEP | Boys | Mid_vs_Low | 0.15 | -0.13 | 0.43 | 0.299 |
| mEP | Boys | High_vs_Low | -0.39 | -0.67 | -0.11 | **0.006** |
| mEP | Boys | High_vs_Mid | -0.54 | -0.85 | -0.24 | **0.000** |
| mEP | Girls | Mid_vs_Low | -0.14 | -0.45 | 0.18 | 0.405 |
| mEP | Girls | High_vs_Low | 0.07 | -0.18 | 0.33 | 0.582 |
| mEP | Girls | High_vs_Mid | 0.21 | -0.09 | 0.51 | 0.178 |
| mCPP | Total | Mid_vs_Low | 0.00 | -0.21 | 0.20 | 0.966 |
| mCPP | Total | High_vs_Low | -0.06 | -0.27 | 0.15 | 0.558 |
| mCPP | Total | High_vs_Mid | -0.06 | -0.27 | 0.15 | 0.585 |
| mCPP | Boys | Mid_vs_Low | -0.20 | -0.48 | 0.08 | 0.159 |
| mCPP | Boys | High_vs_Low | -0.18 | -0.48 | 0.13 | 0.258 |
| mCPP | Boys | High_vs_Mid | 0.02 | -0.25 | 0.30 | 0.868 |
| mCPP | Girls | Mid_vs_Low | 0.30 | 0.01 | 0.59 | **0.043** |
| mCPP | Girls | High_vs_Low | 0.07 | -0.21 | 0.36 | 0.614 |
| mCPP | Girls | High_vs_Mid | -0.23 | -0.53 | 0.07 | 0.134 |
| mIBP | Total | Mid_vs_Low | 0.07 | -0.14 | 0.29 | 0.492 |
| mIBP | Total | High_vs_Low | -0.10 | -0.28 | 0.07 | 0.252 |
| mIBP | Total | High_vs_Mid | -0.18 | -0.38 | 0.02 | 0.076 |
| mIBP | Boys | Mid_vs_Low | 0.12 | -0.15 | 0.40 | 0.371 |
| mIBP | Boys | High_vs_Low | -0.15 | -0.40 | 0.11 | 0.252 |
| mIBP | Boys | High_vs_Mid | -0.27 | -0.56 | 0.02 | 0.064 |
| mIBP | Girls | Mid_vs_Low | 0.14 | -0.17 | 0.45 | 0.364 |
| mIBP | Girls | High_vs_Low | -0.06 | -0.33 | 0.21 | 0.669 |
| mIBP | Girls | High_vs_Mid | -0.20 | -0.50 | 0.09 | 0.176 |
| mBP | Total | Mid_vs_Low | 0.03 | -0.18 | 0.24 | 0.771 |
| mBP | Total | High_vs_Low | -0.05 | -0.24 | 0.14 | 0.621 |
| mBP | Total | High_vs_Mid | -0.08 | -0.28 | 0.12 | 0.435 |
| mBP | Boys | Mid_vs_Low | -0.13 | -0.39 | 0.14 | 0.344 |
| mBP | Boys | High_vs_Low | 0.02 | -0.26 | 0.29 | 0.913 |
| mBP | Boys | High_vs_Mid | 0.14 | -0.11 | 0.39 | 0.259 |
| mBP | Girls | Mid_vs_Low | 0.22 | -0.10 | 0.53 | 0.184 |
| mBP | Girls | High_vs_Low | -0.05 | -0.34 | 0.24 | 0.726 |
| mBP | Girls | High_vs_Mid | -0.27 | -0.55 | 0.01 | 0.060 |
| mECPP | Total | Mid_vs_Low | 0.08 | -0.13 | 0.30 | 0.448 |
| mECPP | Total | High_vs_Low | -0.04 | -0.24 | 0.15 | 0.650 |
| mECPP | Total | High_vs_Mid | -0.13 | -0.33 | 0.08 | 0.220 |
| mECPP | Boys | Mid_vs_Low | 0.20 | -0.08 | 0.47 | 0.155 |
| mECPP | Boys | High_vs_Low | -0.02 | -0.26 | 0.22 | 0.891 |
| mECPP | Boys | High_vs_Mid | -0.21 | -0.49 | 0.06 | 0.127 |
| mECPP | Girls | Mid_vs_Low | -0.04 | -0.36 | 0.28 | 0.797 |
| mECPP | Girls | High_vs_Low | -0.03 | -0.31 | 0.25 | 0.842 |
| mECPP | Girls | High_vs_Mid | 0.01 | -0.29 | 0.32 | 0.929 |
| mCMHP | Total | Mid_vs_Low | 0.04 | -0.17 | 0.25 | 0.684 |
| mCMHP | Total | High_vs_Low | -0.08 | -0.28 | 0.13 | 0.458 |
| mCMHP | Total | High_vs_Mid | -0.12 | -0.35 | 0.10 | 0.290 |
| mCMHP | Boys | Mid_vs_Low | 0.11 | -0.16 | 0.38 | 0.432 |
| mCMHP | Boys | High_vs_Low | 0.06 | -0.25 | 0.37 | 0.691 |
| mCMHP | Boys | High_vs_Mid | -0.05 | -0.38 | 0.29 | 0.784 |
| mCMHP | Girls | Mid_vs_Low | -0.02 | -0.33 | 0.29 | 0.894 |
| mCMHP | Girls | High_vs_Low | -0.17 | -0.45 | 0.10 | 0.220 |
| mCMHP | Girls | High_vs_Mid | -0.15 | -0.43 | 0.13 | 0.286 |
| mEHHP | Total | Mid_vs_Low | 0.06 | -0.16 | 0.27 | 0.609 |
| mEHHP | Total | High_vs_Low | -0.14 | -0.34 | 0.06 | 0.177 |
| mEHHP | Total | High_vs_Mid | -0.19 | -0.39 | 0.00 | 0.055 |
| mEHHP | Boys | Mid_vs_Low | -0.05 | -0.34 | 0.25 | 0.757 |
| mEHHP | Boys | High_vs_Low | -0.12 | -0.41 | 0.17 | 0.430 |
| mEHHP | Boys | High_vs_Mid | -0.07 | -0.35 | 0.21 | 0.622 |
| mEHHP | Girls | Mid_vs_Low | 0.16 | -0.17 | 0.50 | 0.335 |
| mEHHP | Girls | High_vs_Low | -0.14 | -0.43 | 0.15 | 0.340 |
| mEHHP | Girls | High_vs_Mid | -0.30 | -0.59 | -0.02 | **0.035** |
| mBzBP | Total | Mid_vs_Low | -0.01 | -0.30 | 0.28 | 0.962 |
| mBzBP | Total | High_vs_Low | -0.04 | -0.29 | 0.21 | 0.743 |
| mBzBP | Total | High_vs_Mid | -0.03 | -0.29 | 0.22 | 0.785 |
| mBzBP | Boys | Mid_vs_Low | -0.08 | -0.37 | 0.21 | 0.593 |
| mBzBP | Boys | High_vs_Low | 0.02 | -0.27 | 0.30 | 0.904 |
| mBzBP | Boys | High_vs_Mid | 0.10 | -0.20 | 0.40 | 0.523 |
| mBzBP | Girls | Mid_vs_Low | 0.12 | -0.34 | 0.59 | 0.600 |
| mBzBP | Girls | High_vs_Low | -0.05 | -0.42 | 0.32 | 0.787 |
| mBzBP | Girls | High_vs_Mid | -0.17 | -0.57 | 0.23 | 0.392 |
| mEOHP | Total | Mid_vs_Low | 0.06 | -0.17 | 0.29 | 0.626 |
| mEOHP | Total | High_vs_Low | -0.15 | -0.36 | 0.07 | 0.175 |
| mEOHP | Total | High_vs_Mid | -0.21 | -0.40 | -0.01 | **0.041** |
| mEOHP | Boys | Mid_vs_Low | -0.04 | -0.33 | 0.25 | 0.799 |
| mEOHP | Boys | High_vs_Low | -0.10 | -0.39 | 0.20 | 0.520 |
| mEOHP | Boys | High_vs_Mid | -0.06 | -0.33 | 0.22 | 0.685 |
| mEOHP | Girls | Mid_vs_Low | 0.19 | -0.13 | 0.50 | 0.242 |
| mEOHP | Girls | High_vs_Low | -0.18 | -0.45 | 0.10 | 0.202 |
| mEOHP | Girls | High_vs_Mid | -0.37 | -0.63 | -0.10 | **0.007** |

**Table S10. Associations of categorical childhood BPA and phthalate exposure with self-reported internalizing problem score through age 14 years**

| **Exposure** | **Sample** | **Contrast** | **Beta** | **CI lower limit** | **CI upper limit** | **P value** |
| --- | --- | --- | --- | --- | --- | --- |
| BPA | Total | Mid_vs_Low | 0.12 | -0.09 | 0.33 | 0.279 |
| BPA | Total | High_vs_Low | -0.16 | -0.36 | 0.04 | 0.119 |
| BPA | Total | High_vs_Mid | -0.28 | -0.5 | -0.05 | **0.016** |
| BPA | Boys | Mid_vs_Low | -0.05 | -0.29 | 0.18 | 0.666 |
| BPA | Boys | High_vs_Low | -0.30 | -0.53 | -0.06 | **0.016** |
| BPA | Boys | High_vs_Mid | -0.24 | -0.51 | 0.03 | 0.080 |
| BPA | Girls | Mid_vs_Low | 0.14 | -0.23 | 0.51 | 0.467 |
| BPA | Girls | High_vs_Low | -0.18 | -0.53 | 0.17 | 0.311 |
| BPA | Girls | High_vs_Mid | -0.32 | -0.67 | 0.04 | 0.078 |
| PA | Total | Mid_vs_Low | -0.03 | -0.21 | 0.15 | 0.744 |
| PA | Total | High_vs_Low | 0.12 | -0.09 | 0.33 | 0.253 |
| PA | Total | High_vs_Mid | 0.15 | -0.04 | 0.34 | 0.115 |
| PA | Boys | Mid_vs_Low | 0.08 | -0.15 | 0.31 | 0.498 |
| PA | Boys | High_vs_Low | 0.15 | -0.10 | 0.40 | 0.250 |
| PA | Boys | High_vs_Mid | 0.07 | -0.16 | 0.29 | 0.553 |
| PA | Girls | Mid_vs_Low | -0.18 | -0.45 | 0.09 | 0.200 |
| PA | Girls | High_vs_Low | -0.02 | -0.37 | 0.33 | 0.902 |
| PA | Girls | High_vs_Mid | 0.16 | -0.16 | 0.47 | 0.334 |
| mMP | Total | Mid_vs_Low | -0.02 | -0.23 | 0.19 | 0.860 |
| mMP | Total | High_vs_Low | -0.19 | -0.40 | 0.02 | 0.075 |
| mMP | Total | High_vs_Mid | -0.17 | -0.37 | 0.03 | 0.100 |
| mMP | Boys | Mid_vs_Low | 0.06 | -0.22 | 0.33 | 0.696 |
| mMP | Boys | High_vs_Low | -0.18 | -0.45 | 0.08 | 0.179 |
| mMP | Boys | High_vs_Mid | -0.24 | -0.48 | 0.00 | 0.053 |
| mMP | Girls | Mid_vs_Low | -0.18 | -0.47 | 0.12 | 0.237 |
| mMP | Girls | High_vs_Low | -0.25 | -0.59 | 0.08 | 0.139 |
| mMP | Girls | High_vs_Mid | -0.08 | -0.40 | 0.25 | 0.642 |
| mEP | Total | Mid_vs_Low | 0.10 | -0.08 | 0.28 | 0.276 |
| mEP | Total | High_vs_Low | 0.11 | -0.09 | 0.31 | 0.272 |
| mEP | Total | High_vs_Mid | 0.01 | -0.19 | 0.21 | 0.905 |
| mEP | Boys | Mid_vs_Low | 0.23 | 0.00 | 0.45 | **0.045** |
| mEP | Boys | High_vs_Low | 0.04 | -0.23 | 0.32 | 0.746 |
| mEP | Boys | High_vs_Mid | -0.18 | -0.43 | 0.06 | 0.137 |
| mEP | Girls | Mid_vs_Low | -0.03 | -0.31 | 0.25 | 0.832 |
| mEP | Girls | High_vs_Low | 0.19 | -0.10 | 0.48 | 0.189 |
| mEP | Girls | High_vs_Mid | 0.22 | -0.10 | 0.55 | 0.179 |
| mCPP | Total | Mid_vs_Low | 0.14 | -0.06 | 0.34 | 0.173 |
| mCPP | Total | High_vs_Low | 0.04 | -0.15 | 0.23 | 0.683 |
| mCPP | Total | High_vs_Mid | -0.10 | -0.29 | 0.09 | 0.302 |
| mCPP | Boys | Mid_vs_Low | -0.13 | -0.37 | 0.11 | 0.285 |
| mCPP | Boys | High_vs_Low | -0.04 | -0.31 | 0.23 | 0.770 |
| mCPP | Boys | High_vs_Mid | 0.09 | -0.14 | 0.32 | 0.436 |
| mCPP | Girls | Mid_vs_Low | 0.42 | 0.12 | 0.72 | **0.006** |
| mCPP | Girls | High_vs_Low | 0.17 | -0.09 | 0.43 | 0.204 |
| mCPP | Girls | High_vs_Mid | -0.25 | -0.53 | 0.03 | 0.076 |
| mIBP | Total | Mid_vs_Low | 0.05 | -0.15 | 0.25 | 0.644 |
| mIBP | Total | High_vs_Low | 0.04 | -0.15 | 0.23 | 0.682 |
| mIBP | Total | High_vs_Mid | -0.01 | -0.19 | 0.18 | 0.939 |
| mIBP | Boys | Mid_vs_Low | 0.20 | -0.05 | 0.45 | 0.116 |
| mIBP | Boys | High_vs_Low | 0.10 | -0.15 | 0.35 | 0.445 |
| mIBP | Boys | High_vs_Mid | -0.10 | -0.33 | 0.13 | 0.393 |
| mIBP | Girls | Mid_vs_Low | -0.07 | -0.38 | 0.25 | 0.679 |
| mIBP | Girls | High_vs_Low | -0.01 | -0.30 | 0.27 | 0.923 |
| mIBP | Girls | High_vs_Mid | 0.05 | -0.25 | 0.35 | 0.736 |
| mBP | Total | Mid_vs_Low | 0.07 | -0.13 | 0.26 | 0.511 |
| mBP | Total | High_vs_Low | 0.14 | -0.06 | 0.34 | 0.173 |
| mBP | Total | High_vs_Mid | 0.07 | -0.12 | 0.26 | 0.455 |
| mBP | Boys | Mid_vs_Low | -0.08 | -0.32 | 0.16 | 0.508 |
| mBP | Boys | High_vs_Low | 0.17 | -0.09 | 0.43 | 0.204 |
| mBP | Boys | High_vs_Mid | 0.25 | 0.01 | 0.49 | **0.041** |
| mBP | Girls | Mid_vs_Low | 0.15 | -0.17 | 0.46 | 0.369 |
| mBP | Girls | High_vs_Low | 0.12 | -0.18 | 0.42 | 0.431 |
| mBP | Girls | High_vs_Mid | -0.03 | -0.31 | 0.26 | 0.859 |
| mECPP | Total | Mid_vs_Low | 0.17 | -0.02 | 0.36 | 0.077 |
| mECPP | Total | High_vs_Low | 0.15 | -0.03 | 0.33 | 0.111 |
| mECPP | Total | High_vs_Mid | -0.03 | -0.22 | 0.17 | 0.802 |
| mECPP | Boys | Mid_vs_Low | 0.17 | -0.06 | 0.39 | 0.155 |
| mECPP | Boys | High_vs_Low | -0.01 | -0.25 | 0.22 | 0.920 |
| mECPP | Boys | High_vs_Mid | -0.18 | -0.39 | 0.04 | 0.105 |
| mECPP | Girls | Mid_vs_Low | 0.22 | -0.08 | 0.51 | 0.150 |
| mECPP | Girls | High_vs_Low | 0.32 | 0.07 | 0.58 | **0.013** |
| mECPP | Girls | High_vs_Mid | 0.11 | -0.21 | 0.42 | 0.509 |
| mCMHP | Total | Mid_vs_Low | 0.16 | -0.03 | 0.36 | 0.101 |
| mCMHP | Total | High_vs_Low | 0.15 | -0.04 | 0.33 | 0.114 |
| mCMHP | Total | High_vs_Mid | -0.02 | -0.21 | 0.18 | 0.867 |
| mCMHP | Boys | Mid_vs_Low | -0.05 | -0.28 | 0.18 | 0.654 |
| mCMHP | Boys | High_vs_Low | 0.14 | -0.10 | 0.39 | 0.252 |
| mCMHP | Boys | High_vs_Mid | 0.20 | -0.03 | 0.42 | 0.092 |
| mCMHP | Girls | Mid_vs_Low | 0.23 | -0.06 | 0.52 | 0.114 |
| mCMHP | Girls | High_vs_Low | 0.10 | -0.17 | 0.36 | 0.481 |
| mCMHP | Girls | High_vs_Mid | -0.14 | -0.45 | 0.17 | 0.388 |
| mEHHP | Total | Mid_vs_Low | 0.12 | -0.07 | 0.32 | 0.208 |
| mEHHP | Total | High_vs_Low | 0.11 | -0.08 | 0.30 | 0.264 |
| mEHHP | Total | High_vs_Mid | -0.02 | -0.20 | 0.17 | 0.863 |
| mEHHP | Boys | Mid_vs_Low | 0.04 | -0.19 | 0.28 | 0.717 |
| mEHHP | Boys | High_vs_Low | 0.09 | -0.15 | 0.33 | 0.465 |
| mEHHP | Boys | High_vs_Mid | 0.05 | -0.18 | 0.27 | 0.682 |
| mEHHP | Girls | Mid_vs_Low | 0.23 | -0.08 | 0.55 | 0.152 |
| mEHHP | Girls | High_vs_Low | 0.12 | -0.18 | 0.41 | 0.438 |
| mEHHP | Girls | High_vs_Mid | -0.11 | -0.42 | 0.20 | 0.472 |
| mBzBP | Total | Mid_vs_Low | -0.22 | -0.47 | 0.03 | 0.090 |
| mBzBP | Total | High_vs_Low | -0.05 | -0.30 | 0.19 | 0.667 |
| mBzBP | Total | High_vs_Mid | 0.16 | -0.06 | 0.39 | 0.151 |
| mBzBP | Boys | Mid_vs_Low | -0.35 | -0.62 | -0.07 | **0.015** |
| mBzBP | Boys | High_vs_Low | -0.12 | -0.37 | 0.14 | 0.377 |
| mBzBP | Boys | High_vs_Mid | 0.23 | -0.03 | 0.49 | 0.084 |
| mBzBP | Girls | Mid_vs_Low | -0.02 | -0.42 | 0.37 | 0.910 |
| mBzBP | Girls | High_vs_Low | 0.12 | -0.29 | 0.54 | 0.556 |
| mBzBP | Girls | High_vs_Mid | 0.15 | -0.19 | 0.48 | 0.390 |
| mEOHP | Total | Mid_vs_Low | 0.05 | -0.14 | 0.23 | 0.616 |
| mEOHP | Total | High_vs_Low | 0.07 | -0.12 | 0.27 | 0.458 |
| mEOHP | Total | High_vs_Mid | 0.03 | -0.16 | 0.21 | 0.781 |
| mEOHP | Boys | Mid_vs_Low | 0.01 | -0.22 | 0.25 | 0.907 |
| mEOHP | Boys | High_vs_Low | 0.06 | -0.18 | 0.30 | 0.638 |
| mEOHP | Boys | High_vs_Mid | 0.04 | -0.18 | 0.27 | 0.703 |
| mEOHP | Girls | Mid_vs_Low | 0.13 | -0.16 | 0.42 | 0.385 |
| mEOHP | Girls | High_vs_Low | 0.08 | -0.22 | 0.39 | 0.582 |
| mEOHP | Girls | High_vs_Mid | -0.05 | -0.36 | 0.27 | 0.774 |

**Table S11. Associations of categorical childhood BPA and phthalate exposure with parent-reported externalizing problem score through age 14 years**

| **Exposure** | **Sample** | **Contrast** | **Beta** | **CI lower limit** | **CI upper limit** | **P value** |
| --- | --- | --- | --- | --- | --- | --- |
| BPA | Total | Mid_vs_Low | -0.18 | -0.43 | 0.07 | 0.152 |
| BPA | Total | High_vs_Low | -0.24 | -0.47 | -0.01 | **0.039** |
| BPA | Total | High_vs_Mid | -0.05 | -0.26 | 0.15 | 0.609 |
| BPA | Boys | Mid_vs_Low | -0.37 | -0.73 | -0.01 | **0.043** |
| BPA | Boys | High_vs_Low | -0.44 | -0.79 | -0.10 | **0.012** |
| BPA | Boys | High_vs_Mid | -0.07 | -0.40 | 0.26 | 0.675 |
| BPA | Girls | Mid_vs_Low | -0.13 | -0.53 | 0.28 | 0.533 |
| BPA | Girls | High_vs_Low | -0.24 | -0.55 | 0.06 | 0.119 |
| BPA | Girls | High_vs_Mid | -0.12 | -0.40 | 0.17 | 0.422 |
| PA | Total | Mid_vs_Low | 0.09 | -0.12 | 0.29 | 0.406 |
| PA | Total | High_vs_Low | 0.06 | -0.14 | 0.26 | 0.553 |
| PA | Total | High_vs_Mid | -0.03 | -0.23 | 0.18 | 0.803 |
| PA | Boys | Mid_vs_Low | 0.06 | -0.23 | 0.34 | 0.696 |
| PA | Boys | High_vs_Low | -0.06 | -0.32 | 0.21 | 0.680 |
| PA | Boys | High_vs_Mid | -0.11 | -0.39 | 0.17 | 0.427 |
| PA | Girls | Mid_vs_Low | 0.12 | -0.16 | 0.40 | 0.395 |
| PA | Girls | High_vs_Low | 0.11 | -0.17 | 0.39 | 0.435 |
| PA | Girls | High_vs_Mid | -0.01 | -0.27 | 0.25 | 0.927 |
| mMP | Total | Mid_vs_Low | -0.06 | -0.30 | 0.18 | 0.626 |
| mMP | Total | High_vs_Low | -0.21 | -0.45 | 0.04 | 0.100 |
| mMP | Total | High_vs_Mid | -0.15 | -0.38 | 0.09 | 0.229 |
| mMP | Boys | Mid_vs_Low | -0.14 | -0.49 | 0.22 | 0.454 |
| mMP | Boys | High_vs_Low | -0.29 | -0.64 | 0.06 | 0.104 |
| mMP | Boys | High_vs_Mid | -0.15 | -0.48 | 0.17 | 0.357 |
| mMP | Girls | Mid_vs_Low | -0.12 | -0.44 | 0.19 | 0.445 |
| mMP | Girls | High_vs_Low | -0.18 | -0.49 | 0.14 | 0.278 |
| mMP | Girls | High_vs_Mid | -0.05 | -0.37 | 0.26 | 0.741 |
| mEP | Total | Mid_vs_Low | 0.05 | -0.15 | 0.25 | 0.649 |
| mEP | Total | High_vs_Low | -0.08 | -0.29 | 0.13 | 0.446 |
| mEP | Total | High_vs_Mid | -0.13 | -0.33 | 0.08 | 0.231 |
| mEP | Boys | Mid_vs_Low | 0.05 | -0.23 | 0.33 | 0.711 |
| mEP | Boys | High_vs_Low | -0.29 | -0.56 | -0.02 | **0.035** |
| mEP | Boys | High_vs_Mid | -0.35 | -0.62 | -0.07 | **0.014** |
| mEP | Girls | Mid_vs_Low | 0.00 | -0.28 | 0.29 | 0.993 |
| mEP | Girls | High_vs_Low | 0.15 | -0.14 | 0.43 | 0.316 |
| mEP | Girls | High_vs_Mid | 0.15 | -0.17 | 0.46 | 0.363 |
| mCPP | Total | Mid_vs_Low | 0.08 | -0.13 | 0.29 | 0.457 |
| mCPP | Total | High_vs_Low | -0.01 | -0.21 | 0.18 | 0.902 |
| mCPP | Total | High_vs_Mid | -0.09 | -0.30 | 0.12 | 0.389 |
| mCPP | Boys | Mid_vs_Low | -0.06 | -0.33 | 0.22 | 0.693 |
| mCPP | Boys | High_vs_Low | -0.08 | -0.38 | 0.22 | 0.622 |
| mCPP | Boys | High_vs_Mid | -0.02 | -0.31 | 0.27 | 0.894 |
| mCPP | Girls | Mid_vs_Low | 0.32 | 0.06 | 0.59 | **0.017** |
| mCPP | Girls | High_vs_Low | 0.08 | -0.15 | 0.30 | 0.500 |
| mCPP | Girls | High_vs_Mid | -0.25 | -0.50 | 0.01 | 0.056 |
| mIBP | Total | Mid_vs_Low | -0.03 | -0.24 | 0.18 | 0.788 |
| mIBP | Total | High_vs_Low | -0.02 | -0.23 | 0.19 | 0.842 |
| mIBP | Total | High_vs_Mid | 0.01 | -0.19 | 0.21 | 0.937 |
| mIBP | Boys | Mid_vs_Low | -0.03 | -0.31 | 0.25 | 0.840 |
| mIBP | Boys | High_vs_Low | -0.11 | -0.41 | 0.19 | 0.480 |
| mIBP | Boys | High_vs_Mid | -0.08 | -0.36 | 0.20 | 0.577 |
| mIBP | Girls | Mid_vs_Low | 0.07 | -0.22 | 0.36 | 0.643 |
| mIBP | Girls | High_vs_Low | 0.12 | -0.15 | 0.39 | 0.393 |
| mIBP | Girls | High_vs_Mid | 0.05 | -0.22 | 0.31 | 0.725 |
| mBP | Total | Mid_vs_Low | -0.03 | -0.26 | 0.20 | 0.783 |
| mBP | Total | High_vs_Low | -0.09 | -0.30 | 0.12 | 0.406 |
| mBP | Total | High_vs_Mid | -0.06 | -0.25 | 0.13 | 0.557 |
| mBP | Boys | Mid_vs_Low | -0.12 | -0.45 | 0.21 | 0.463 |
| mBP | Boys | High_vs_Low | -0.15 | -0.44 | 0.14 | 0.312 |
| mBP | Boys | High_vs_Mid | -0.03 | -0.31 | 0.26 | 0.857 |
| mBP | Girls | Mid_vs_Low | 0.04 | -0.25 | 0.33 | 0.797 |
| mBP | Girls | High_vs_Low | -0.05 | -0.34 | 0.25 | 0.757 |
| mBP | Girls | High_vs_Mid | -0.08 | -0.32 | 0.15 | 0.486 |
| mECPP | Total | Mid_vs_Low | 0.15 | -0.04 | 0.33 | 0.124 |
| mECPP | Total | High_vs_Low | 0.08 | -0.13 | 0.29 | 0.474 |
| mECPP | Total | High_vs_Mid | -0.07 | -0.29 | 0.15 | 0.537 |
| mECPP | Boys | Mid_vs_Low | 0.18 | -0.09 | 0.44 | 0.192 |
| mECPP | Boys | High_vs_Low | 0.11 | -0.21 | 0.42 | 0.506 |
| mECPP | Boys | High_vs_Mid | -0.07 | -0.37 | 0.23 | 0.642 |
| mECPP | Girls | Mid_vs_Low | 0.20 | -0.04 | 0.44 | 0.101 |
| mECPP | Girls | High_vs_Low | 0.13 | -0.12 | 0.37 | 0.302 |
| mECPP | Girls | High_vs_Mid | -0.07 | -0.36 | 0.21 | 0.618 |
| mCMHP | Total | Mid_vs_Low | -0.09 | -0.30 | 0.12 | 0.407 |
| mCMHP | Total | High_vs_Low | -0.10 | -0.32 | 0.11 | 0.341 |
| mCMHP | Total | High_vs_Mid | -0.02 | -0.23 | 0.20 | 0.887 |
| mCMHP | Boys | Mid_vs_Low | -0.05 | -0.33 | 0.23 | 0.717 |
| mCMHP | Boys | High_vs_Low | -0.01 | -0.31 | 0.30 | 0.968 |
| mCMHP | Boys | High_vs_Mid | 0.05 | -0.24 | 0.33 | 0.756 |
| mCMHP | Girls | Mid_vs_Low | -0.14 | -0.43 | 0.14 | 0.327 |
| mCMHP | Girls | High_vs_Low | -0.13 | -0.39 | 0.13 | 0.340 |
| mCMHP | Girls | High_vs_Mid | 0.02 | -0.27 | 0.30 | 0.914 |
| mEHHP | Total | Mid_vs_Low | 0.04 | -0.17 | 0.25 | 0.698 |
| mEHHP | Total | High_vs_Low | -0.02 | -0.23 | 0.20 | 0.888 |
| mEHHP | Total | High_vs_Mid | -0.06 | -0.26 | 0.15 | 0.588 |
| mEHHP | Boys | Mid_vs_Low | 0.02 | -0.24 | 0.28 | 0.853 |
| mEHHP | Boys | High_vs_Low | 0.11 | -0.17 | 0.39 | 0.448 |
| mEHHP | Boys | High_vs_Mid | 0.09 | -0.22 | 0.39 | 0.582 |
| mEHHP | Girls | Mid_vs_Low | -0.01 | -0.35 | 0.33 | 0.964 |
| mEHHP | Girls | High_vs_Low | -0.13 | -0.42 | 0.15 | 0.359 |
| mEHHP | Girls | High_vs_Mid | -0.13 | -0.44 | 0.19 | 0.437 |
| mBzBP | Total | Mid_vs_Low | 0.05 | -0.22 | 0.32 | 0.719 |
| mBzBP | Total | High_vs_Low | 0.08 | -0.21 | 0.37 | 0.594 |
| mBzBP | Total | High_vs_Mid | 0.03 | -0.24 | 0.30 | 0.827 |
| mBzBP | Boys | Mid_vs_Low | -0.04 | -0.40 | 0.31 | 0.810 |
| mBzBP | Boys | High_vs_Low | 0.02 | -0.37 | 0.41 | 0.934 |
| mBzBP | Boys | High_vs_Mid | 0.06 | -0.26 | 0.38 | 0.710 |
| mBzBP | Girls | Mid_vs_Low | 0.17 | -0.21 | 0.55 | 0.388 |
| mBzBP | Girls | High_vs_Low | 0.19 | -0.30 | 0.69 | 0.447 |
| mBzBP | Girls | High_vs_Mid | 0.02 | -0.38 | 0.43 | 0.905 |
| mEOHP | Total | Mid_vs_Low | 0.09 | -0.11 | 0.29 | 0.394 |
| mEOHP | Total | High_vs_Low | -0.01 | -0.22 | 0.19 | 0.901 |
| mEOHP | Total | High_vs_Mid | -0.10 | -0.32 | 0.12 | 0.367 |
| mEOHP | Boys | Mid_vs_Low | -0.01 | -0.27 | 0.24 | 0.920 |
| mEOHP | Boys | High_vs_Low | 0.08 | -0.21 | 0.36 | 0.587 |
| mEOHP | Boys | High_vs_Mid | 0.09 | -0.21 | 0.40 | 0.554 |
| mEOHP | Girls | Mid_vs_Low | 0.18 | -0.14 | 0.50 | 0.274 |
| mEOHP | Girls | High_vs_Low | -0.10 | -0.35 | 0.15 | 0.444 |
| mEOHP | Girls | High_vs_Mid | -0.28 | -0.59 | 0.04 | 0.091 |

**Table S12. Associations of categorical childhood BPA and phthalate exposure with self-reported externalizing problem score through age 14 years**

| **Exposure** | **Sample** | **Contrast** | **Beta** | **CI lower limit** | **CI upper limit** | **P value** |
| --- | --- | --- | --- | --- | --- | --- |
| BPA | Total | Mid_vs_Low | 0.06 | -0.16 | 0.28 | 0.619 |
| BPA | Total | High_vs_Low | -0.21 | -0.41 | -0.01 | **0.042** |
| BPA | Total | High_vs_Mid | -0.27 | -0.48 | -0.05 | **0.014** |
| BPA | Boys | Mid_vs_Low | 0.15 | -0.17 | 0.48 | 0.345 |
| BPA | Boys | High_vs_Low | -0.32 | -0.64 | 0.00 | 0.050 |
| BPA | Boys | High_vs_Mid | -0.47 | -0.81 | -0.14 | **0.006** |
| BPA | Girls | Mid_vs_Low | -0.14 | -0.47 | 0.18 | 0.381 |
| BPA | Girls | High_vs_Low | -0.30 | -0.60 | 0.00 | **0.047** |
| BPA | Girls | High_vs_Mid | -0.16 | -0.45 | 0.13 | 0.273 |
| PA | Total | Mid_vs_Low | 0.06 | -0.13 | 0.25 | 0.537 |
| PA | Total | High_vs_Low | 0.00 | -0.20 | 0.20 | 0.996 |
| PA | Total | High_vs_Mid | -0.06 | -0.25 | 0.13 | 0.544 |
| PA | Boys | Mid_vs_Low | 0.22 | -0.05 | 0.48 | 0.113 |
| PA | Boys | High_vs_Low | -0.10 | -0.38 | 0.17 | 0.462 |
| PA | Boys | High_vs_Mid | -0.32 | -0.57 | -0.07 | **0.013** |
| PA | Girls | Mid_vs_Low | -0.05 | -0.30 | 0.20 | 0.683 |
| PA | Girls | High_vs_Low | 0.06 | -0.23 | 0.34 | 0.702 |
| PA | Girls | High_vs_Mid | 0.11 | -0.18 | 0.40 | 0.465 |
| mMP | Total | Mid_vs_Low | 0.09 | -0.14 | 0.32 | 0.443 |
| mMP | Total | High_vs_Low | -0.11 | -0.31 | 0.09 | 0.272 |
| mMP | Total | High_vs_Mid | -0.20 | -0.43 | 0.03 | 0.085 |
| mMP | Boys | Mid_vs_Low | 0.23 | -0.10 | 0.56 | 0.170 |
| mMP | Boys | High_vs_Low | -0.18 | -0.44 | 0.07 | 0.163 |
| mMP | Boys | High_vs_Mid | -0.42 | -0.71 | -0.12 | **0.005** |
| mMP | Girls | Mid_vs_Low | -0.14 | -0.42 | 0.14 | 0.315 |
| mMP | Girls | High_vs_Low | -0.12 | -0.45 | 0.21 | 0.487 |
| mMP | Girls | High_vs_Mid | 0.03 | -0.29 | 0.35 | 0.872 |
| mEP | Total | Mid_vs_Low | -0.01 | -0.22 | 0.19 | 0.897 |
| mEP | Total | High_vs_Low | -0.19 | -0.40 | 0.02 | 0.083 |
| mEP | Total | High_vs_Mid | -0.18 | -0.37 | 0.01 | 0.070 |
| mEP | Boys | Mid_vs_Low | 0.03 | -0.25 | 0.30 | 0.845 |
| mEP | Boys | High_vs_Low | -0.32 | -0.62 | -0.02 | **0.038** |
| mEP | Boys | High_vs_Mid | -0.35 | -0.60 | -0.09 | **0.007** |
| mEP | Girls | Mid_vs_Low | -0.08 | -0.38 | 0.22 | 0.596 |
| mEP | Girls | High_vs_Low | -0.04 | -0.34 | 0.26 | 0.804 |
| mEP | Girls | High_vs_Mid | 0.04 | -0.26 | 0.35 | 0.784 |
| mCPP | Total | Mid_vs_Low | 0.08 | -0.12 | 0.29 | 0.435 |
| mCPP | Total | High_vs_Low | -0.08 | -0.27 | 0.11 | 0.405 |
| mCPP | Total | High_vs_Mid | -0.16 | -0.36 | 0.03 | 0.098 |
| mCPP | Boys | Mid_vs_Low | 0.00 | -0.28 | 0.27 | 0.994 |
| mCPP | Boys | High_vs_Low | 0.07 | -0.23 | 0.36 | 0.652 |
| mCPP | Boys | High_vs_Mid | 0.07 | -0.21 | 0.35 | 0.628 |
| mCPP | Girls | Mid_vs_Low | 0.25 | -0.05 | 0.54 | 0.101 |
| mCPP | Girls | High_vs_Low | -0.11 | -0.35 | 0.14 | 0.390 |
| mCPP | Girls | High_vs_Mid | -0.36 | -0.62 | -0.09 | **0.008** |
| mIBP | Total | Mid_vs_Low | -0.05 | -0.25 | 0.14 | 0.594 |
| mIBP | Total | High_vs_Low | -0.06 | -0.26 | 0.14 | 0.569 |
| mIBP | Total | High_vs_Mid | -0.01 | -0.20 | 0.19 | 0.955 |
| mIBP | Boys | Mid_vs_Low | 0.16 | -0.12 | 0.44 | 0.256 |
| mIBP | Boys | High_vs_Low | -0.11 | -0.41 | 0.20 | 0.488 |
| mIBP | Boys | High_vs_Mid | -0.27 | -0.52 | -0.02 | **0.036** |
| mIBP | Girls | Mid_vs_Low | -0.21 | -0.48 | 0.06 | 0.125 |
| mIBP | Girls | High_vs_Low | 0.02 | -0.27 | 0.32 | 0.875 |
| mIBP | Girls | High_vs_Mid | 0.24 | -0.05 | 0.53 | 0.111 |
| mBP | Total | Mid_vs_Low | 0.04 | -0.15 | 0.24 | 0.686 |
| mBP | Total | High_vs_Low | 0.05 | -0.15 | 0.25 | 0.614 |
| mBP | Total | High_vs_Mid | 0.01 | -0.19 | 0.21 | 0.912 |
| mBP | Boys | Mid_vs_Low | 0.04 | -0.25 | 0.32 | 0.794 |
| mBP | Boys | High_vs_Low | -0.03 | -0.28 | 0.22 | 0.802 |
| mBP | Boys | High_vs_Mid | -0.07 | -0.36 | 0.22 | 0.639 |
| mBP | Girls | Mid_vs_Low | -0.02 | -0.29 | 0.25 | 0.882 |
| mBP | Girls | High_vs_Low | 0.13 | -0.17 | 0.42 | 0.403 |
| mBP | Girls | High_vs_Mid | 0.15 | -0.12 | 0.41 | 0.289 |
| mECPP | Total | Mid_vs_Low | 0.05 | -0.14 | 0.24 | 0.609 |
| mECPP | Total | High_vs_Low | -0.08 | -0.29 | 0.13 | 0.450 |
| mECPP | Total | High_vs_Mid | -0.13 | -0.34 | 0.08 | 0.225 |
| mECPP | Boys | Mid_vs_Low | -0.04 | -0.29 | 0.20 | 0.730 |
| mECPP | Boys | High_vs_Low | -0.17 | -0.46 | 0.12 | 0.251 |
| mECPP | Boys | High_vs_Mid | -0.13 | -0.40 | 0.14 | 0.356 |
| mECPP | Girls | Mid_vs_Low | 0.24 | -0.02 | 0.50 | 0.075 |
| mECPP | Girls | High_vs_Low | 0.05 | -0.23 | 0.33 | 0.710 |
| mECPP | Girls | High_vs_Mid | -0.19 | -0.49 | 0.12 | 0.230 |
| mCMHP | Total | Mid_vs_Low | 0.07 | -0.14 | 0.27 | 0.532 |
| mCMHP | Total | High_vs_Low | -0.10 | -0.30 | 0.10 | 0.341 |
| mCMHP | Total | High_vs_Mid | -0.16 | -0.36 | 0.03 | 0.097 |
| mCMHP | Boys | Mid_vs_Low | -0.02 | -0.31 | 0.26 | 0.872 |
| mCMHP | Boys | High_vs_Low | -0.17 | -0.47 | 0.13 | 0.275 |
| mCMHP | Boys | High_vs_Mid | -0.14 | -0.40 | 0.11 | 0.270 |
| mCMHP | Girls | Mid_vs_Low | 0.10 | -0.19 | 0.40 | 0.501 |
| mCMHP | Girls | High_vs_Low | -0.02 | -0.30 | 0.26 | 0.878 |
| mCMHP | Girls | High_vs_Mid | -0.12 | -0.43 | 0.18 | 0.433 |
| mEHHP | Total | Mid_vs_Low | -0.01 | -0.21 | 0.20 | 0.958 |
| mEHHP | Total | High_vs_Low | -0.12 | -0.32 | 0.08 | 0.242 |
| mEHHP | Total | High_vs_Mid | -0.11 | -0.31 | 0.09 | 0.266 |
| mEHHP | Boys | Mid_vs_Low | -0.02 | -0.30 | 0.25 | 0.867 |
| mEHHP | Boys | High_vs_Low | -0.16 | -0.42 | 0.10 | 0.229 |
| mEHHP | Boys | High_vs_Mid | -0.14 | -0.41 | 0.14 | 0.337 |
| mEHHP | Girls | Mid_vs_Low | 0.05 | -0.22 | 0.32 | 0.717 |
| mEHHP | Girls | High_vs_Low | -0.05 | -0.34 | 0.23 | 0.720 |
| mEHHP | Girls | High_vs_Mid | -0.10 | -0.39 | 0.19 | 0.492 |
| mBzBP | Total | Mid_vs_Low | 0.04 | -0.21 | 0.29 | 0.777 |
| mBzBP | Total | High_vs_Low | 0.07 | -0.20 | 0.35 | 0.596 |
| mBzBP | Total | High_vs_Mid | 0.04 | -0.25 | 0.33 | 0.800 |
| mBzBP | Boys | Mid_vs_Low | 0.01 | -0.35 | 0.36 | 0.977 |
| mBzBP | Boys | High_vs_Low | 0.42 | 0.06 | 0.79 | **0.022** |
| mBzBP | Boys | High_vs_Mid | 0.42 | 0.05 | 0.79 | **0.027** |
| mBzBP | Girls | Mid_vs_Low | 0.22 | -0.19 | 0.63 | 0.293 |
| mBzBP | Girls | High_vs_Low | -0.09 | -0.53 | 0.35 | 0.693 |
| mBzBP | Girls | High_vs_Mid | -0.31 | -0.71 | 0.09 | 0.131 |
| mEOHP | Total | Mid_vs_Low | -0.01 | -0.21 | 0.18 | 0.881 |
| mEOHP | Total | High_vs_Low | -0.12 | -0.32 | 0.08 | 0.239 |
| mEOHP | Total | High_vs_Mid | -0.11 | -0.31 | 0.10 | 0.306 |
| mEOHP | Boys | Mid_vs_Low | 0.02 | -0.24 | 0.28 | 0.879 |
| mEOHP | Boys | High_vs_Low | -0.16 | -0.42 | 0.10 | 0.229 |
| mEOHP | Boys | High_vs_Mid | -0.18 | -0.45 | 0.09 | 0.188 |
| mEOHP | Girls | Mid_vs_Low | -0.03 | -0.30 | 0.24 | 0.804 |
| mEOHP | Girls | High_vs_Low | -0.09 | -0.38 | 0.21 | 0.566 |
| mEOHP | Girls | High_vs_Mid | -0.05 | -0.34 | 0.24 | 0.730 |
