# Supplementary material for: Prenatal and childhood exposure to common plasticizers in relation to emotional and behavioral development through adolescence
Source: Sci Total Environ. Author manuscript; Available in PMC 2026 Jul 1. (PMC13320848; doi:10.1016/j.scitotenv.2026.181869)
Supplement: Supplementary Materials [file NIHMS2185941-supplement-Supplementary_Materials.zip › 1-s2.0-S0048969726005334-mmc1.docx]

Supplement for “Prenatal and childhood exposure to common plasticizers in relation to emotional and behavioral development through adolescence”

# Figures

Women with complete set of three urine samples (early, mid- and late pregnancy)

(n = 2089)

Children with neurobehavioral, biospecimen and sociodemographic data

(n = 1405)

Children with age 6 urine samples

(n = 1461)

**Exclusion:**

(n = 313)

**Exclusion:**

(n = 684/ n= 628)

Women included between February 2004 and January 2006, when spot urine sample were collected

(n = 4918)

Women with a urine sample in early pregnancy

(n = 2402)

**Exclusion:**

(n =2516)

Women with complete set of three urine samples (early, mid- and late pregnancy)

(n = 2089)

Children with neurobehavioral, biospecimen and sociodemographic data

(n = 1405)

Maternal pregnancy urine sample measured at three timepoints

(n = 1379)

Children with outcome data at least at one time point (3, 6, 9 and/or 13 years)

(n = 1361)

Children with age 6 years urine samples

(n = 1461)

Age 6 years urine samples measured

(n = 775)

Children with outcome data at least at one time point (9 and/or 13 years)

(n = 651)

**Exclusion:**

(n = 35 / n= 124)

**Exclusion:**

(n =9/686)

**Exclusion:**

(n = 313)

**Exclusion:**

(n = 684/ n= 628)

Women included between February 2004 and January 2006, when spot urine sample were collected

(n = 4918)

Women with a urine sample in early pregnancy

(n = 2402)

**Exclusion:**

(n =2516)

**Figure S1. Flowchart of study population**

*Figure note:*  for 25 children with childhood (6 years) bisphenol and phthalate metabolite concentration analysis no prenatal bisphenol and phthalate metabolite concentration analysis is available


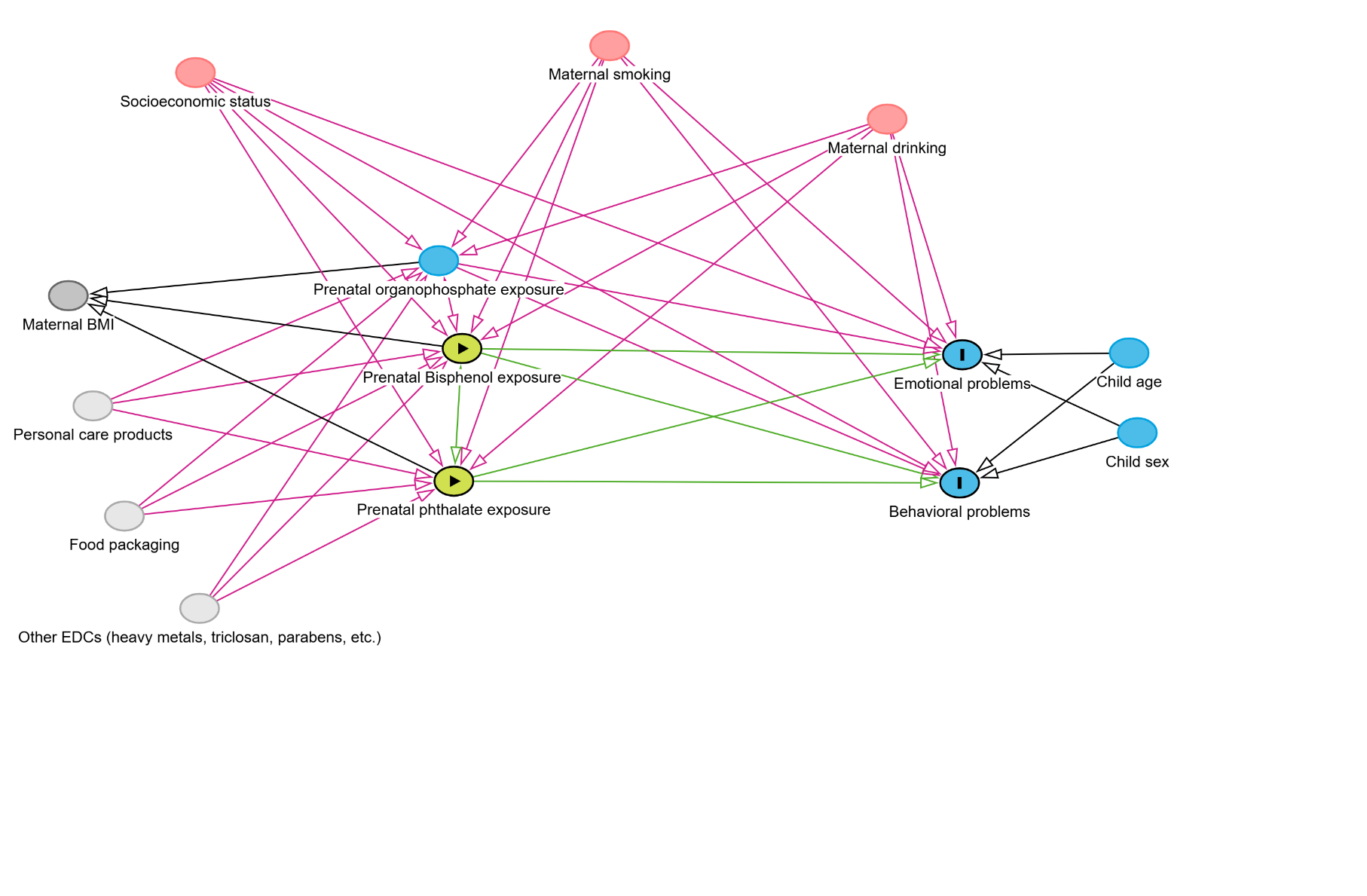


**A.** DAG for prenatal models


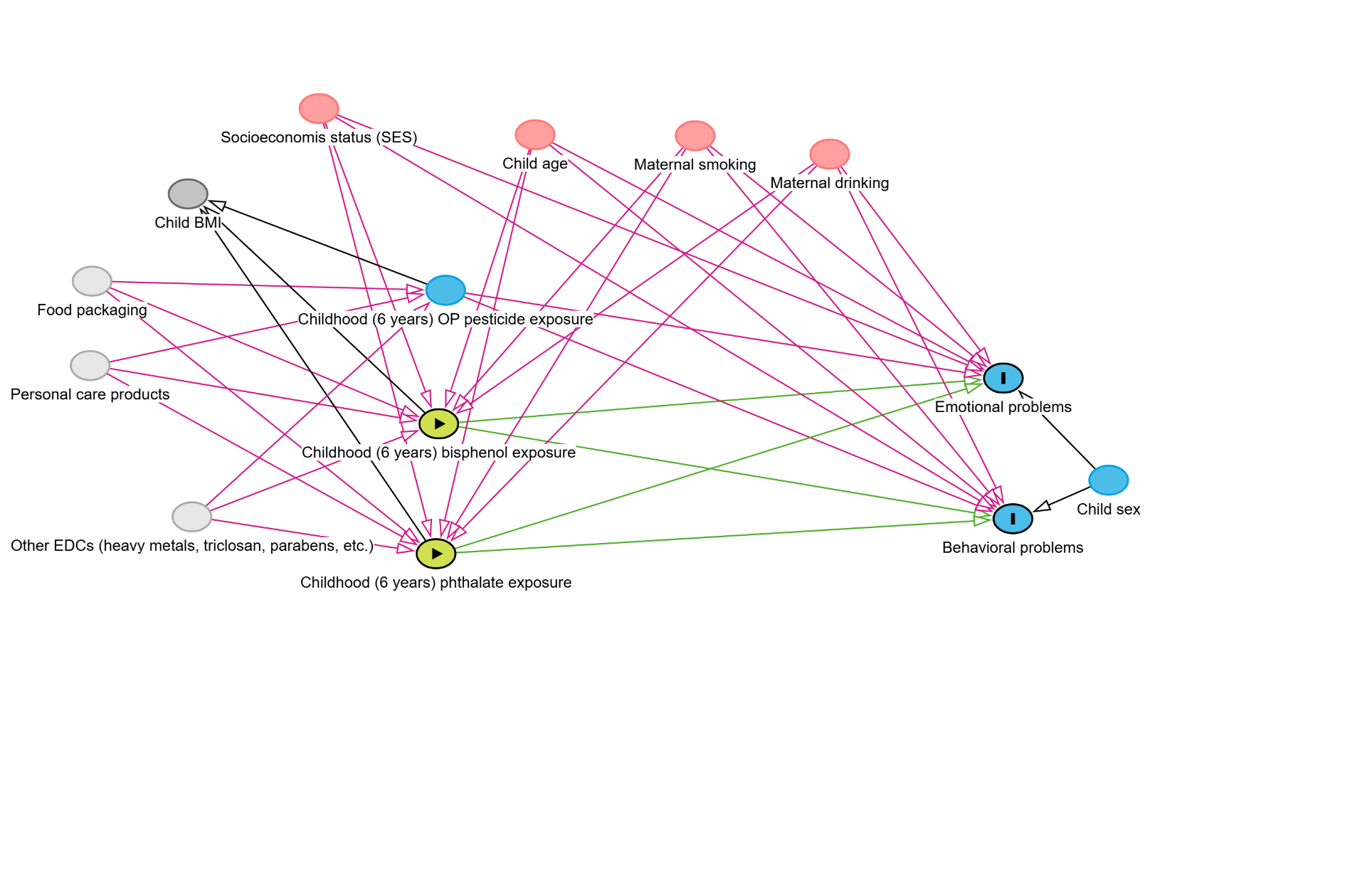


**B.** DAG for childhood models

**Figure S2. Directed Acyclic Graph for prenatal and childhood models.** Models for examining the relation of bisphenol and phthalate exposure with internalizing and externalizing problems from childhood to adolescence. Maternal and child characteristics that potentially affect the association between bisphenol and phthalate exposure in pregnancy and childhood and child internalizing problems and externalizing problems are shown.

**A**. Correlation plot of chemicals measured in early, mid and late pregnancy^*^

**
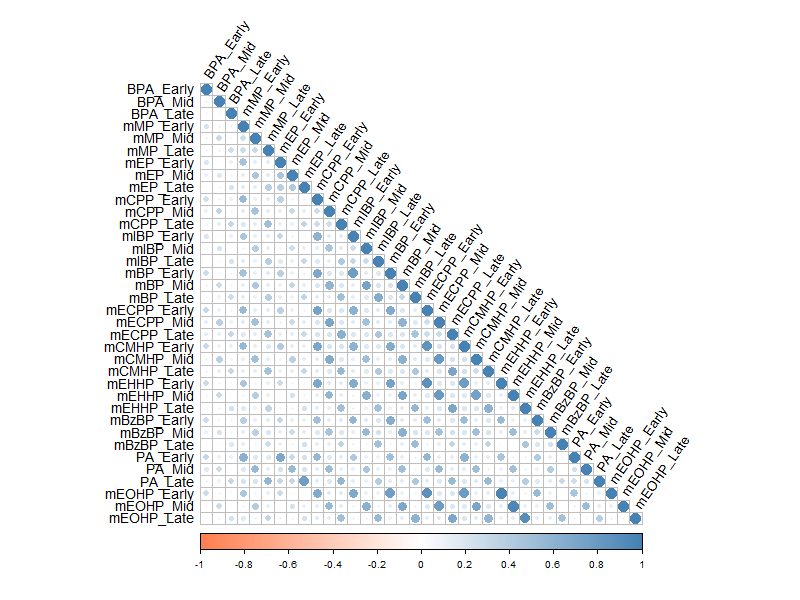
**

**B**. Correlation plot of chemicals measured in childhood

**
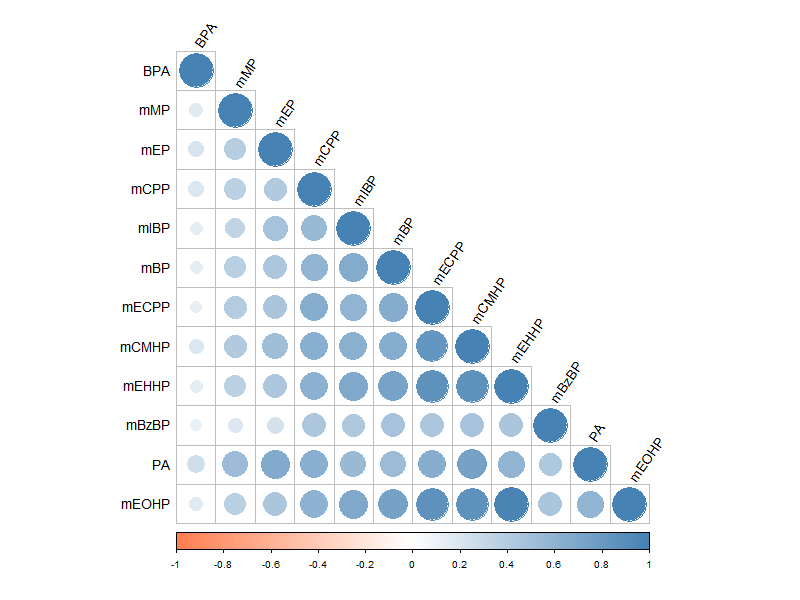
**

**
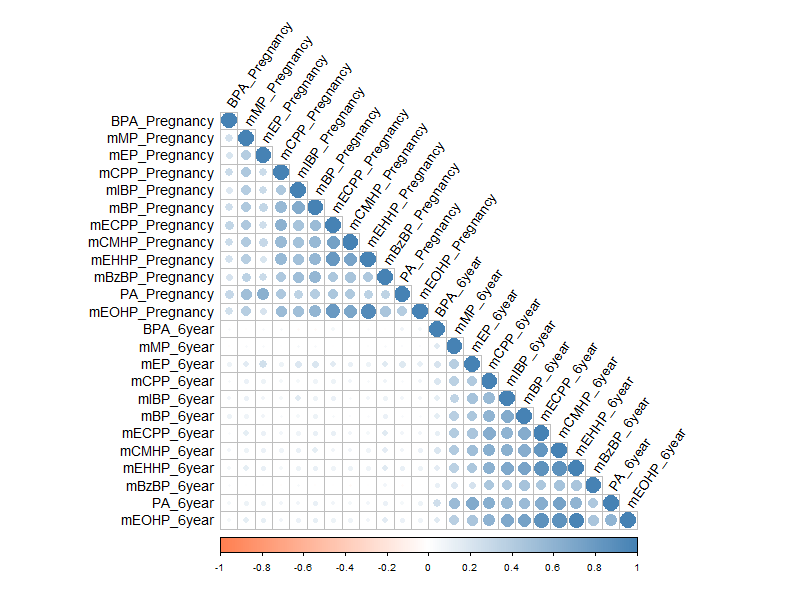
**

**C**. Correlation plot of chemicals measured in pregnancy (average) and childhood

**Figure S3. Spearman** **correlation plots of bisphenol and phthalate metabolite concentrations in spot urine samples.** The Y-axis shows the name of the chemicals and the X-axis shows the strength of the correlation between each pair of chemicals. BPA: Bisphenol A, mMP: mono-methyl phthalate, mEP: mono-ethyl phthalate, mCPP: mono(3-carboxypropyl) phthalate, mIBP: mono-isobutyl phthalate, mBP: mono-n-butyl phthalate, mECPP: mono-(2-ethyl-5-carboxypentyl) phthalate, mCMHP: mono-[(2-carboxymethyl)hexyl]phthalate, mEHHP: mono-(2-ethyl-5-hydroxyhexyl)phthalate, mBzP: monobenzyl phthalate, mINP: monoisononylphthalate, mCHP: mono-cyclohexyl, mOP: monooctylphthalate, PA: phthalic acid, mIDP: mono-hydroxy-isodecyl phthalate, mHxP: mono-hexylphthalate, mHpP: mono-2-heptylphthalate, mCHpP: mono(7-carboxyheptyl) and mEOHP: mono-(2-ethyl-5-oxohexyl) phthalate.

* Early: <18 weeks pregnancy; mid: 18 – 25 weeks pregnancy; late: > 25 weeks pregnancy

**
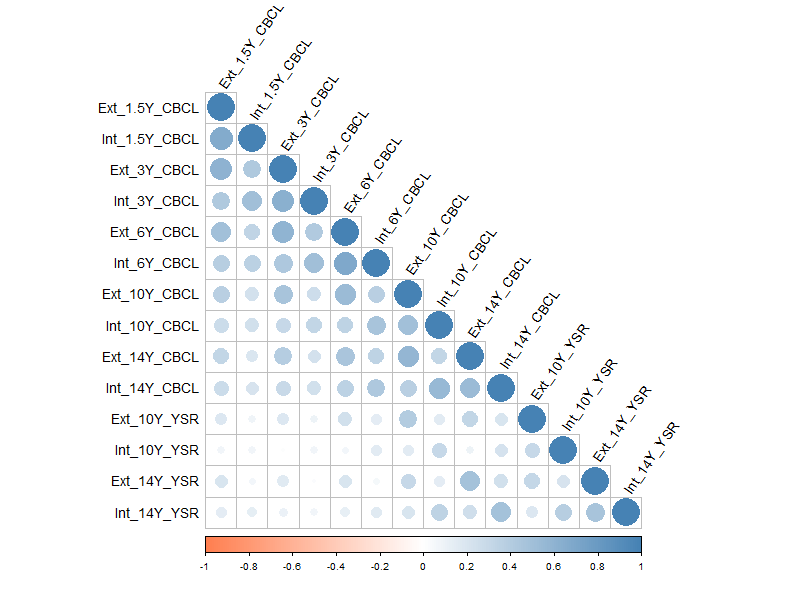
Figure S4. Spearman** **correlation plots of parent-reported and child self-reported internalizing and externalizing problem scores.** The Y-axis shows at which ages the internalizing and externalizing problem scores were reported and by which rater. The X-axis shows the strength of the correlation between each pair of outcome scores. Ext: Externalizing problem score, Int: Internalizing problem score, Y: Years, CBCL: Child Behavioral Checklist (parent-report), YSR: Youth-Self-Report (self-report).

A. Total sample


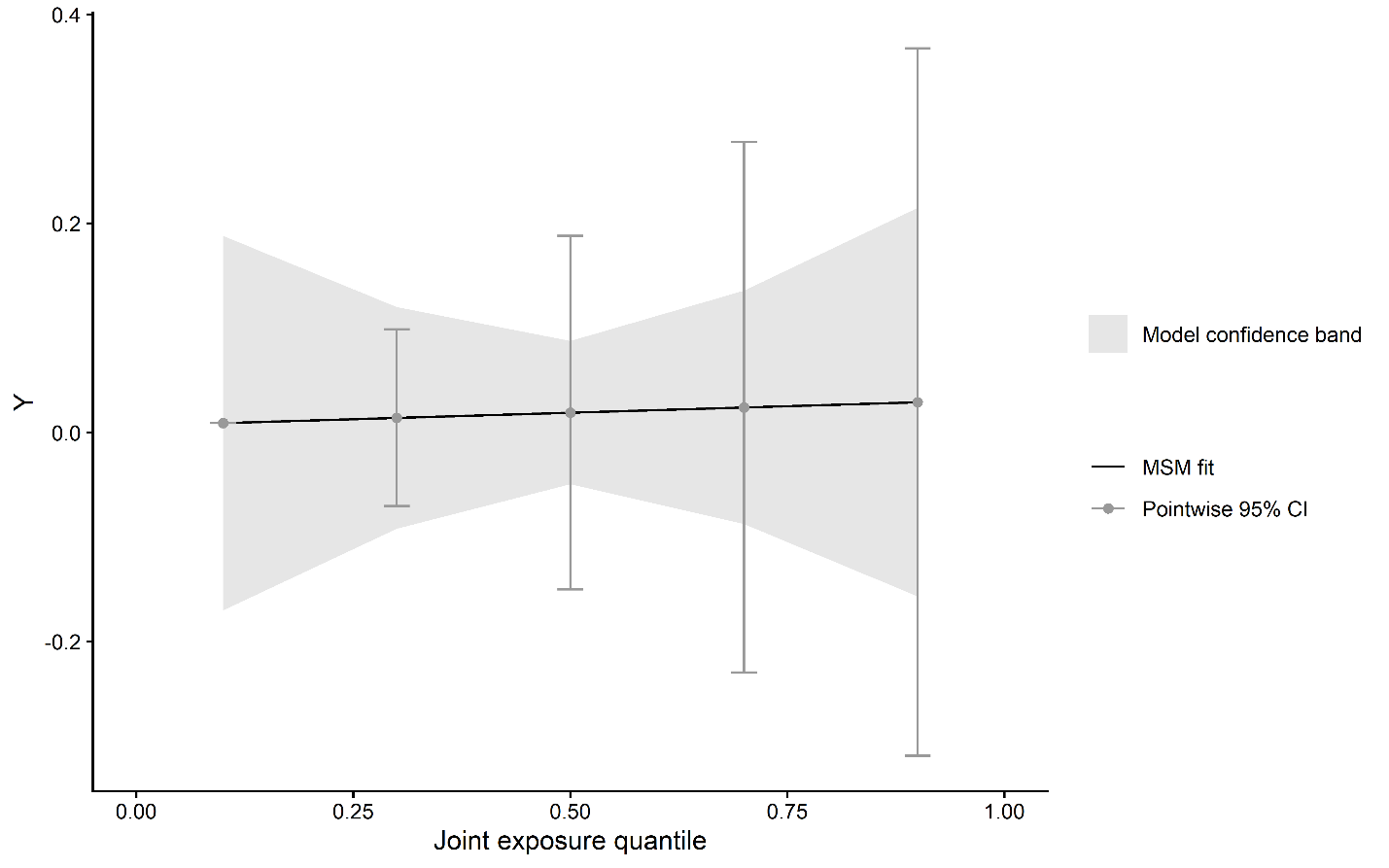


B. Boys


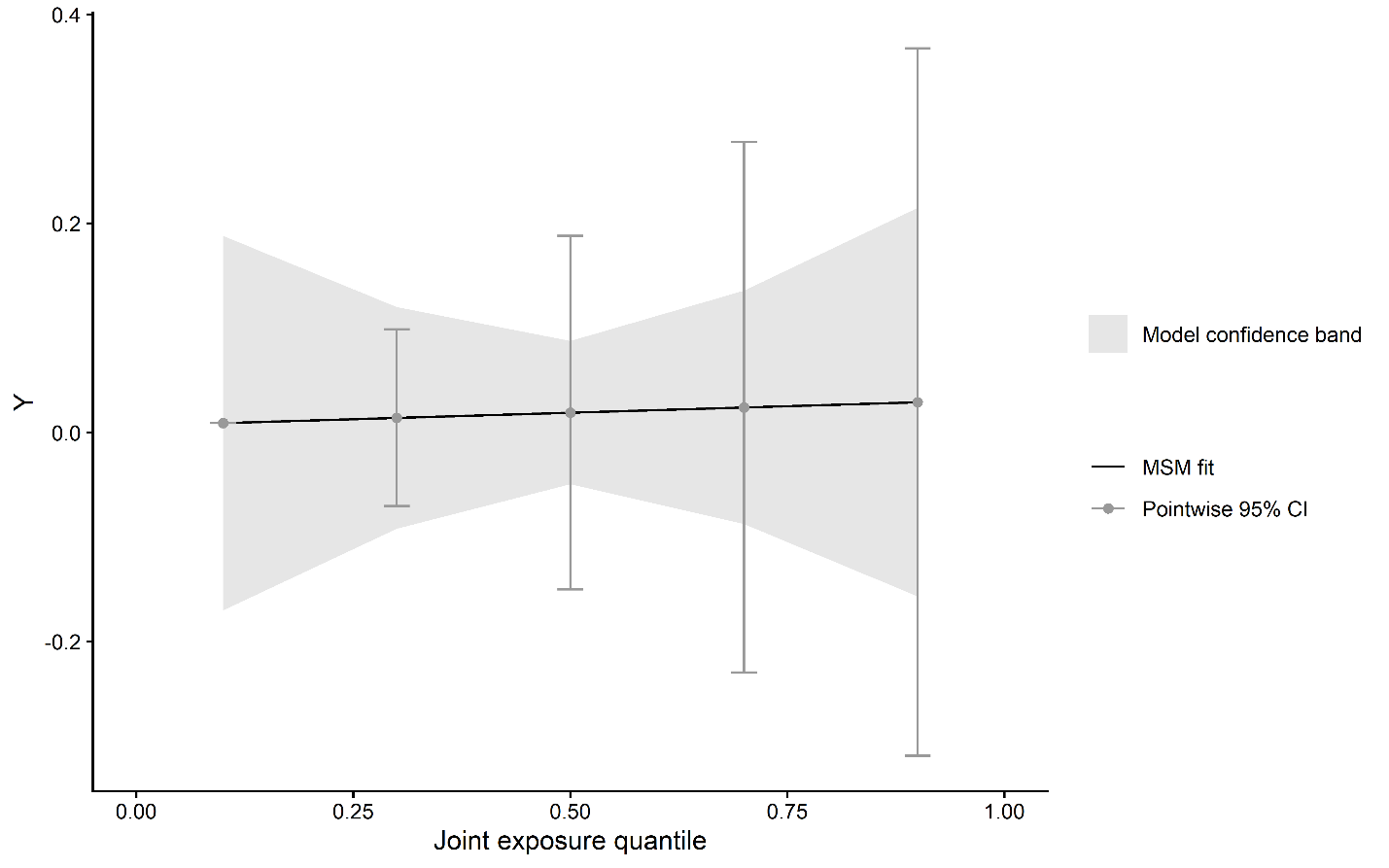


C. Girls


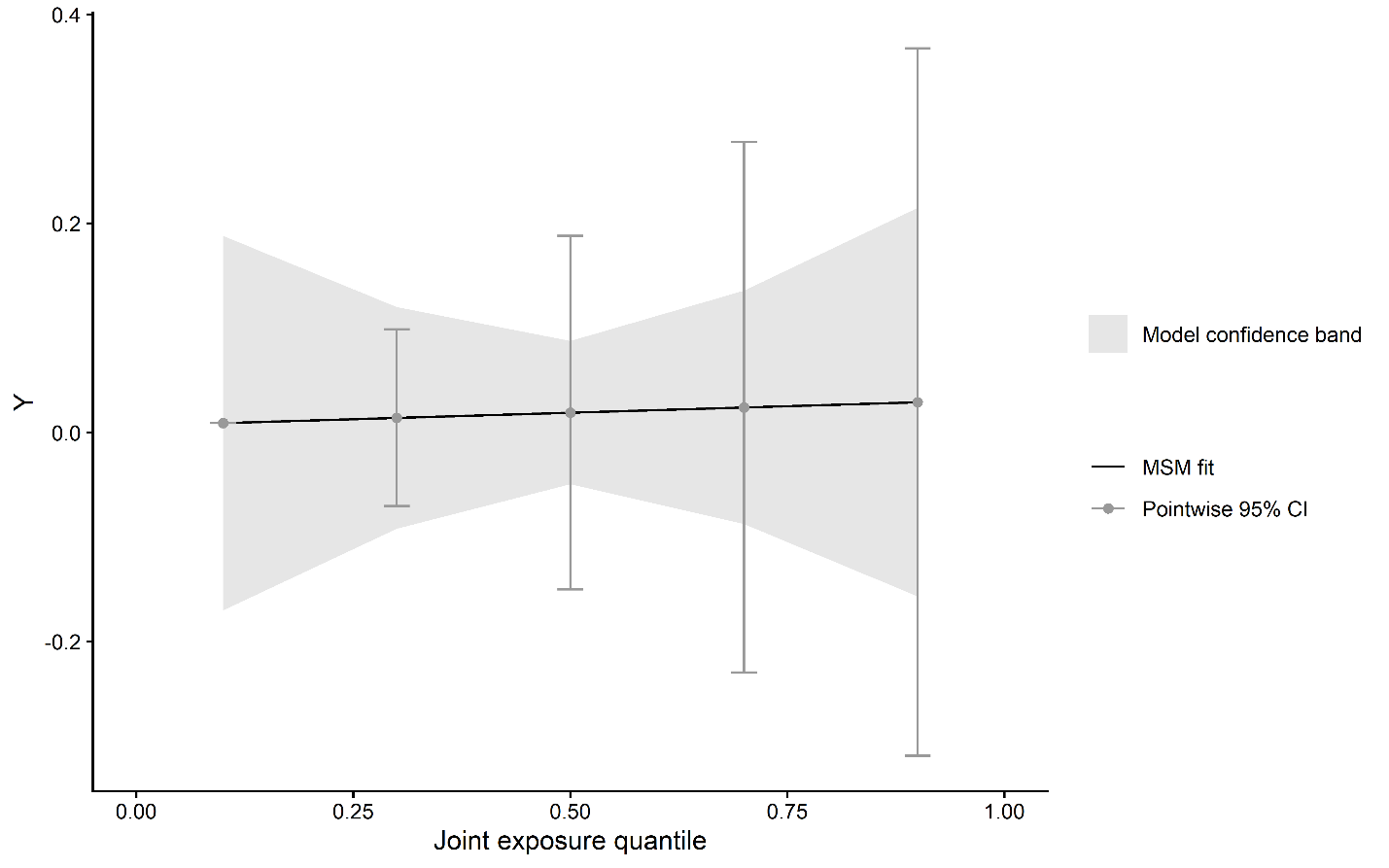


**Figure S5. Joint effect of the prenatal exposure mixture on internalizing problem scores at age 14 years.**

*Figure note:* The line depicts the estimated differences in internalizing problem scores for one quantile increase in all prenatal chemical exposure in the mixture simultaneously (psi = 0.001, p = 0.98), conditional on covariates. The estimates are plotted at the midpoint of each quantile (corresponding to the 10th, 30th, 50th, 70th and 90th percentile). Error bars represent the pointwise 95% confidence intervals around the estimate at each quantile compared to the lowest quantile. Pregnancy chemical concentrations were creatinine adjusted, log 10 transformed, and averaged, prior to including them in the models. Internalizing problem scores were standardized with calculating Z-scores.

Models were adjusted for maternal age, pre-pregnancy body mass index, parity, country of origin, maternal educational levels, marital status, maternal smoking and alcohol drinking habits, as well as gestational age at the time of chemical measurements, child sex (only in models with all children), child age at outcome measurement, and urinary concentrations of organophosphate pesticides during pregnancy.A. Total sample


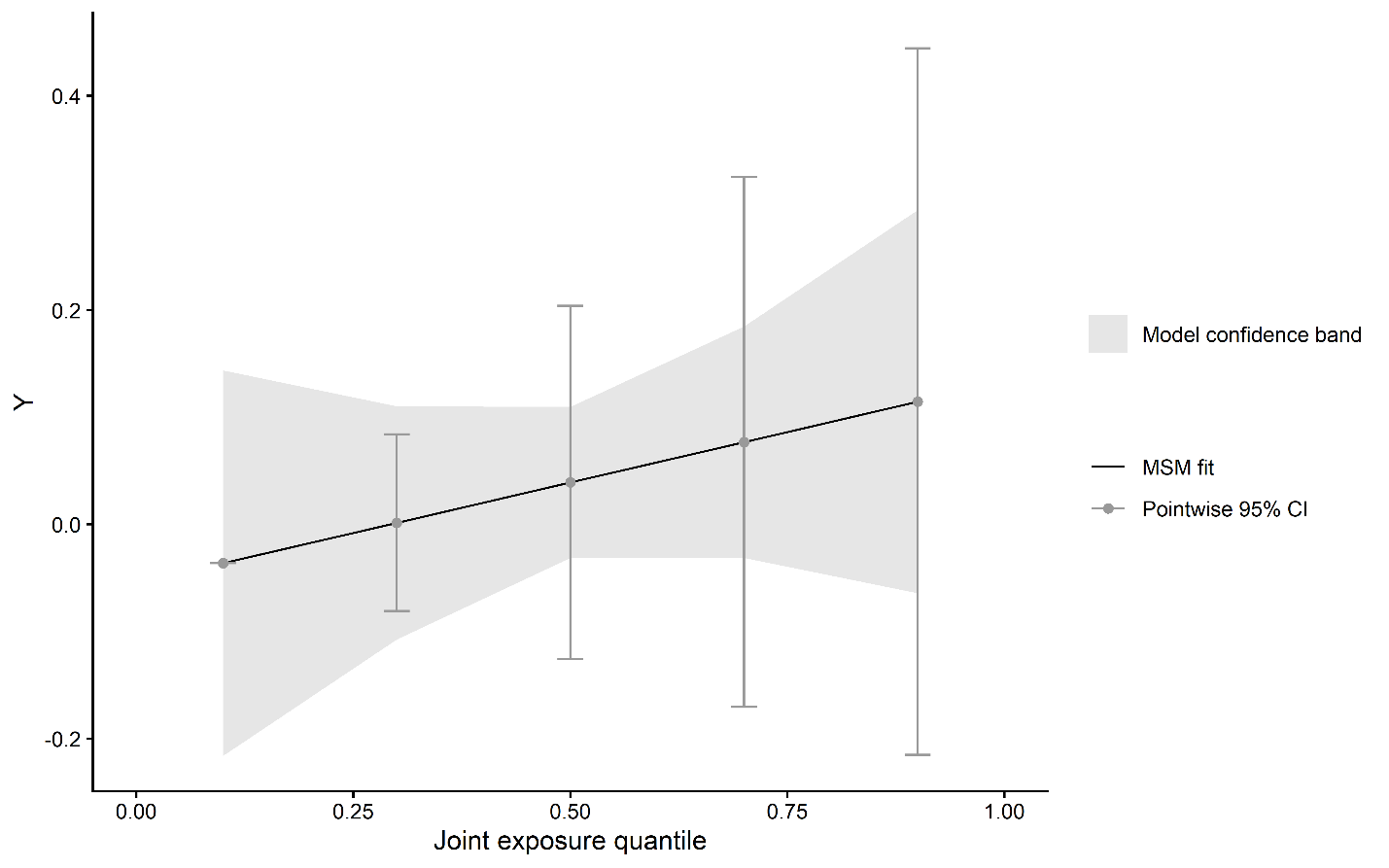


B. Boys


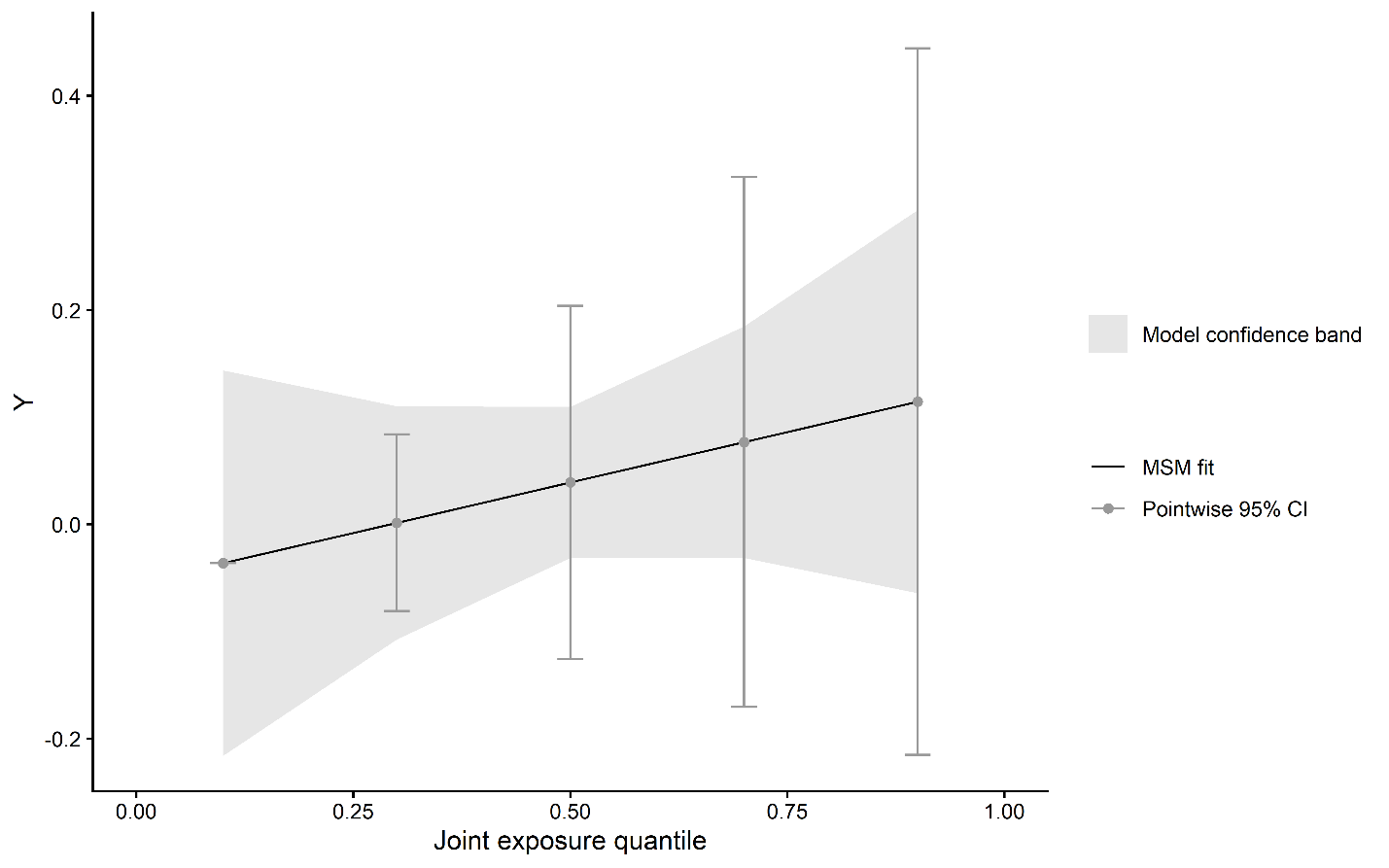


C. Girls


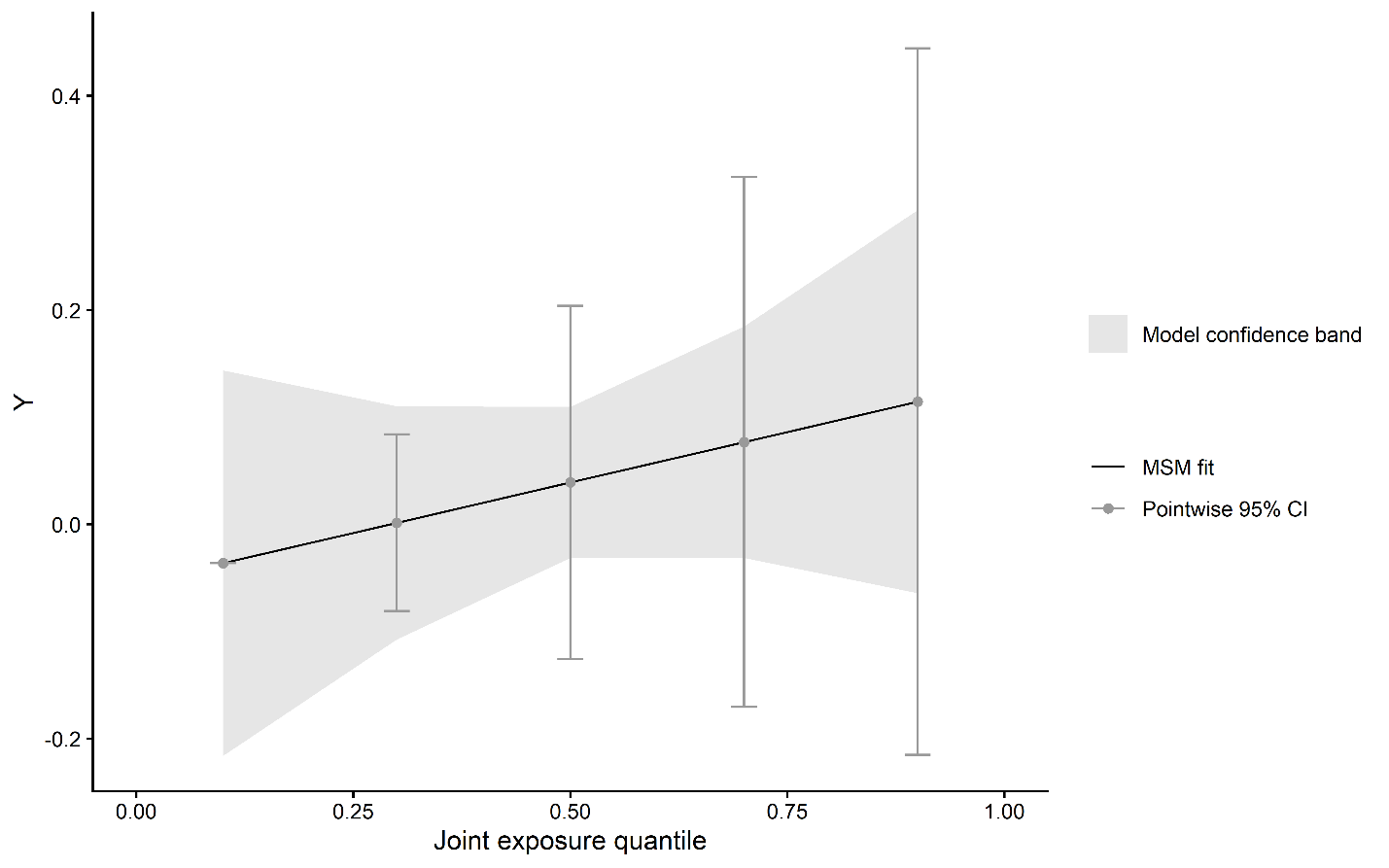


**Figure S6. Joint effect of the prenatal exposure mixture on externalizing problem scores at age 14 years.**

*Figure note:* The line depicts the estimated differences in externalizing problem scores for one quantile increase in all prenatal chemical exposure in the mixture simultaneously (psi = 0.045, p = 0.44), conditional on covariates. The estimates are plotted at the midpoint of each quantile (corresponding to the 10th, 30th, 50th, 70th and 90th percentile). Error bars represent the pointwise 95% confidence intervals around the estimate at each quantile compared to the lowest quantile. Pregnancy chemical concentrations were creatinine adjusted, log 10 transformed, and averaged, prior to including them in the models. Externalizing problem scores were standardized with calculating Z-scores.

Models were adjusted for maternal age, pre-pregnancy body mass index, parity, country of origin, maternal educational levels, marital status, maternal smoking and alcohol drinking habits, as well as gestational age at the time of chemical measurements, child sex (only in models with all children), child age at outcome measurement, and urinary concentrations of organophosphate pesticides during pregnancy.

A. Total sample


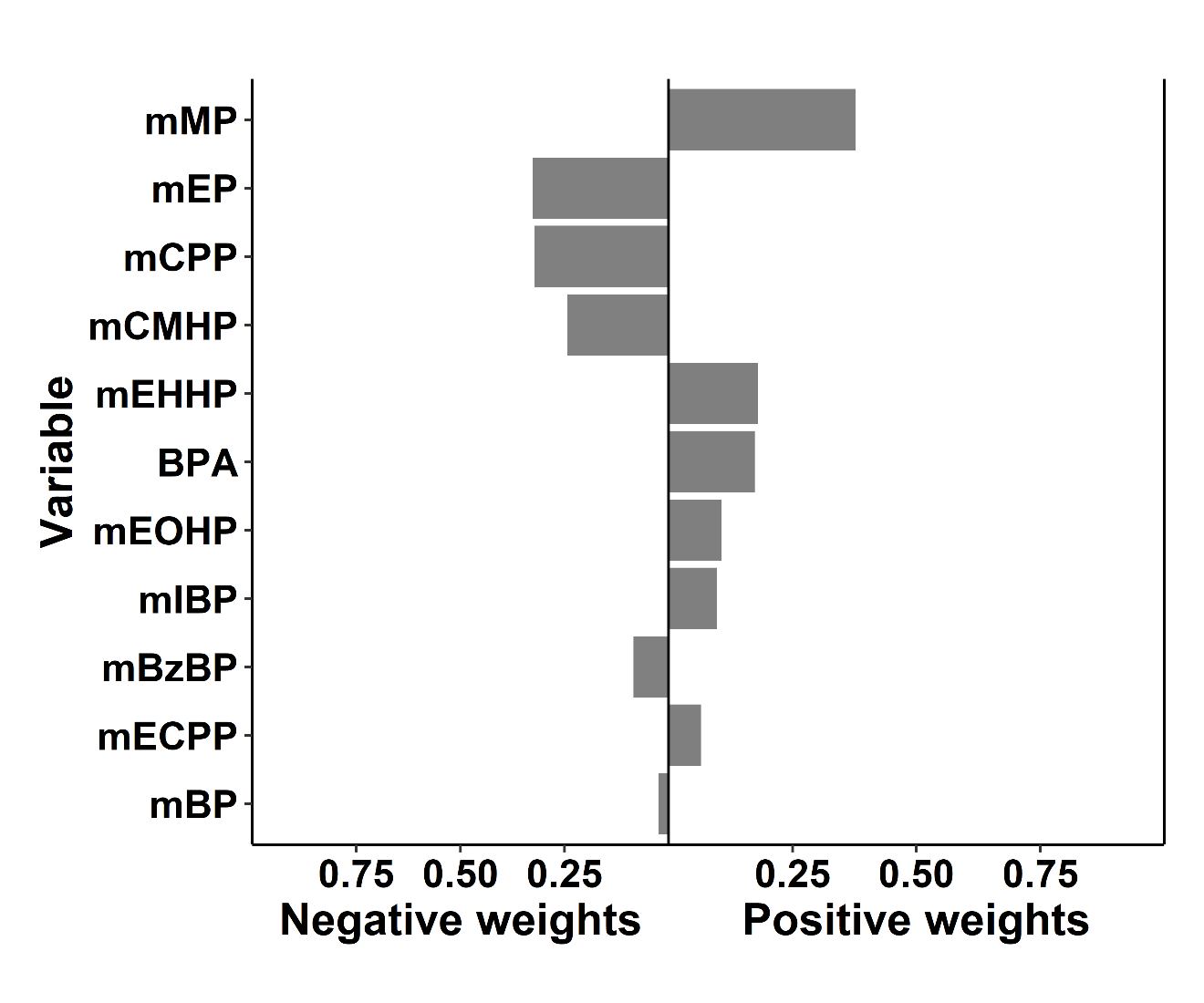


B. Boys


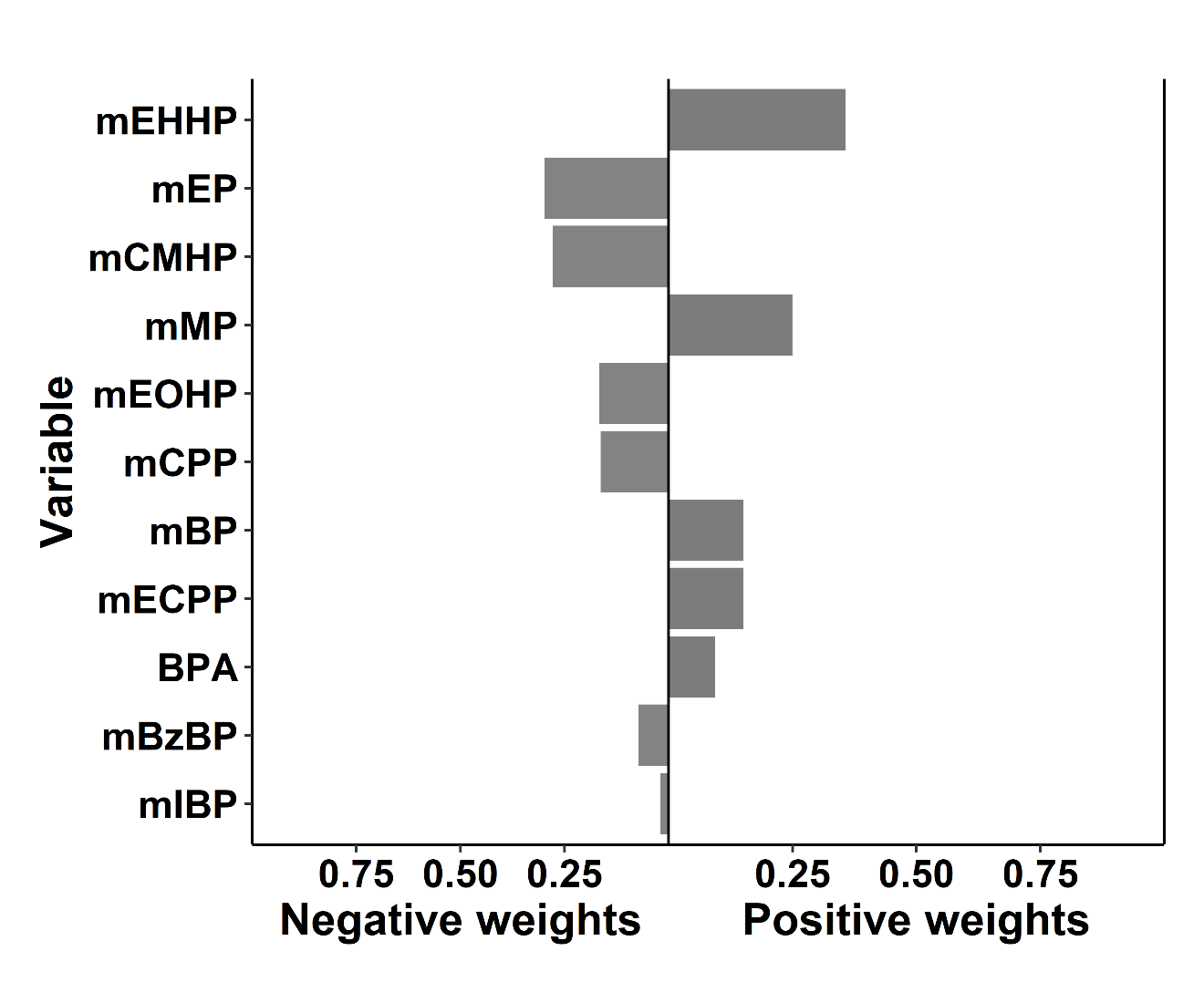


C. Girls


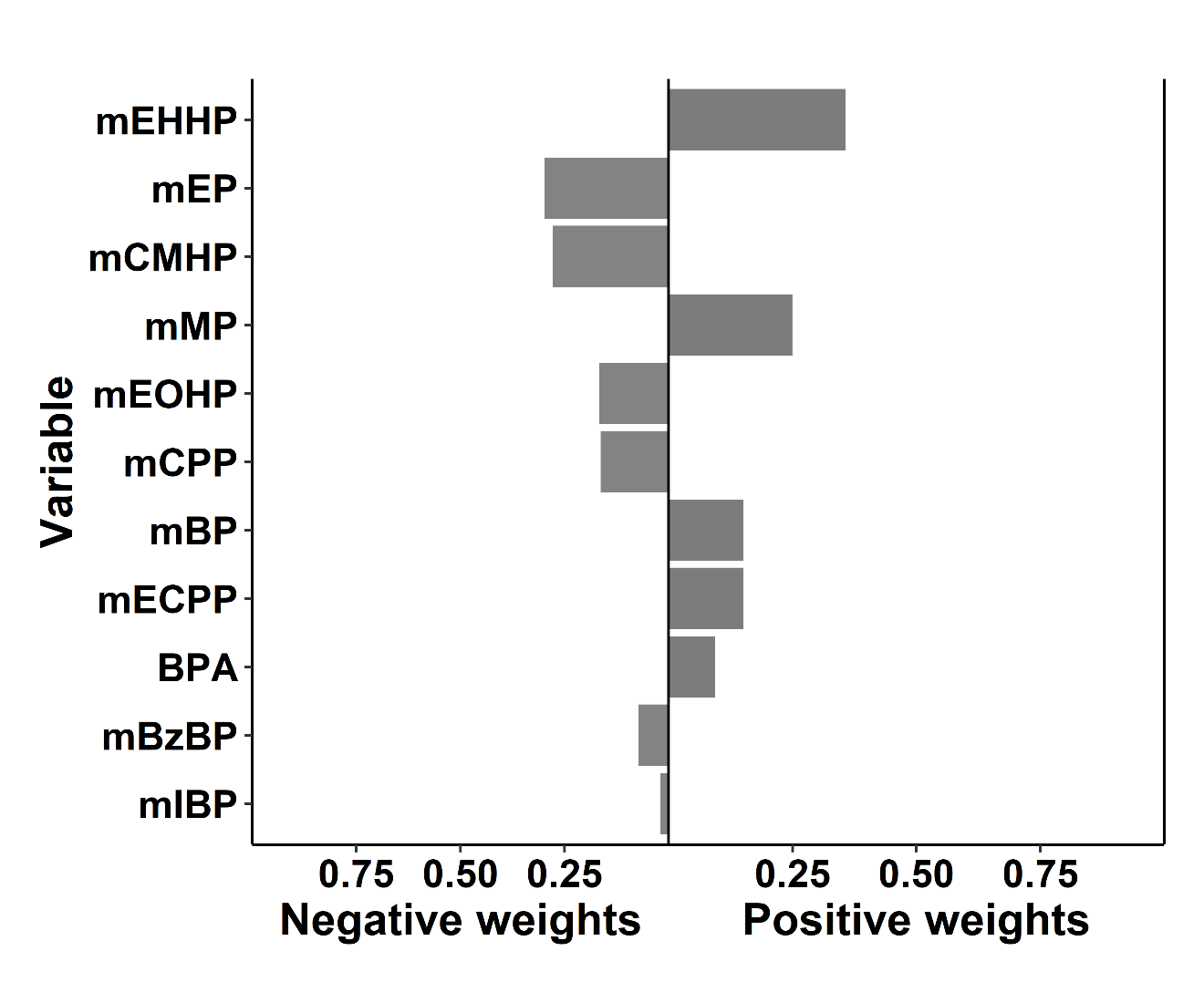


**Figure S7. Weight distribution for the effects of prenatal BPA and phthalate metabolite levels on internalizing problem scores at age 14 years.**

*Figure note:* The distribution of weights for each metabolite in the prenatal exposure mixture is depicted, reflecting the proportion of the overall positive or negative effect attributed to each individual metabolite on internalizing problem scores. Positive weights indicate metabolites contributing to the positive direction of the overall effect, while negative weights reflect metabolites contributing to the negative direction. Interpretation is limited to metabolites contributing to the same direction as the joint effect (positive or negative). Pregnancy chemical concentrations were creatinine adjusted, log 10 transformed, and averaged, prior to including them in the models. Internalizing problem scores were standardized with calculating Z-scores.

Models were adjusted for maternal age, pre-pregnancy body mass index, parity, country of origin, maternal educational levels, marital status, maternal smoking and alcohol drinking habits, as well as gestational age at the time of chemical measurements, child sex (only in models with all children), child age at outcome measurement, and urinary concentrations of organophosphate pesticides during pregnancy.

A. Total sample


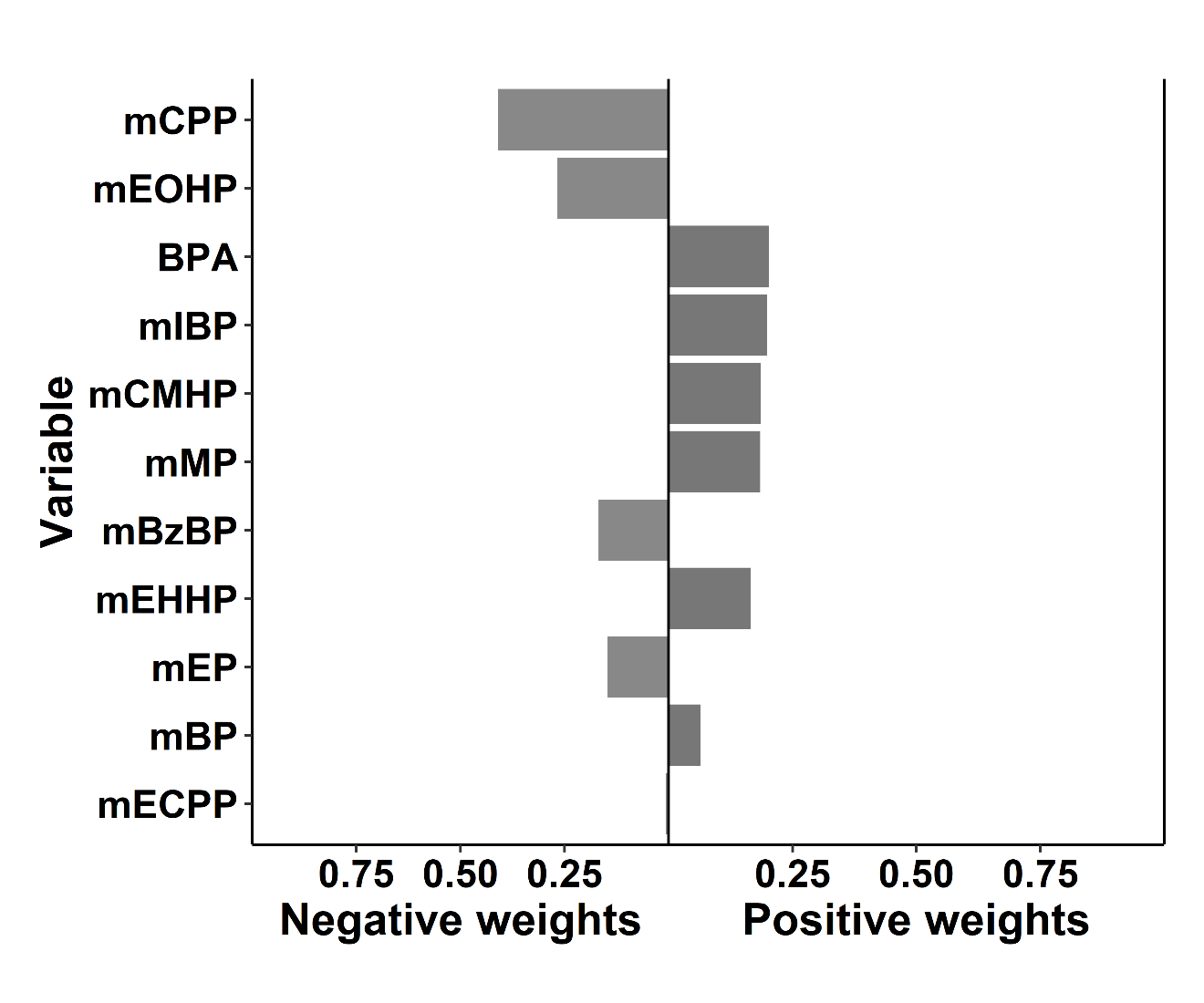


B. Boys


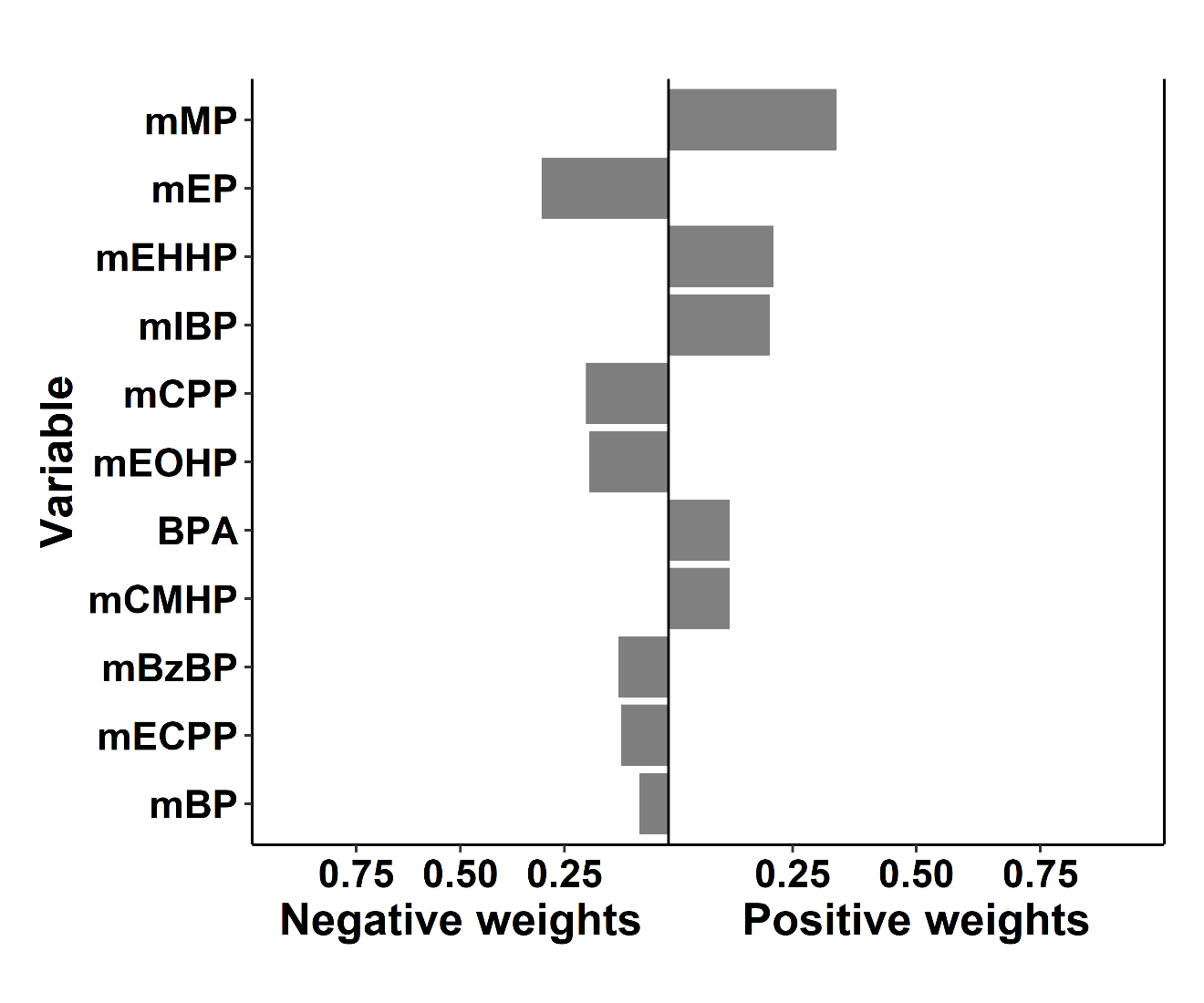


C. Girls


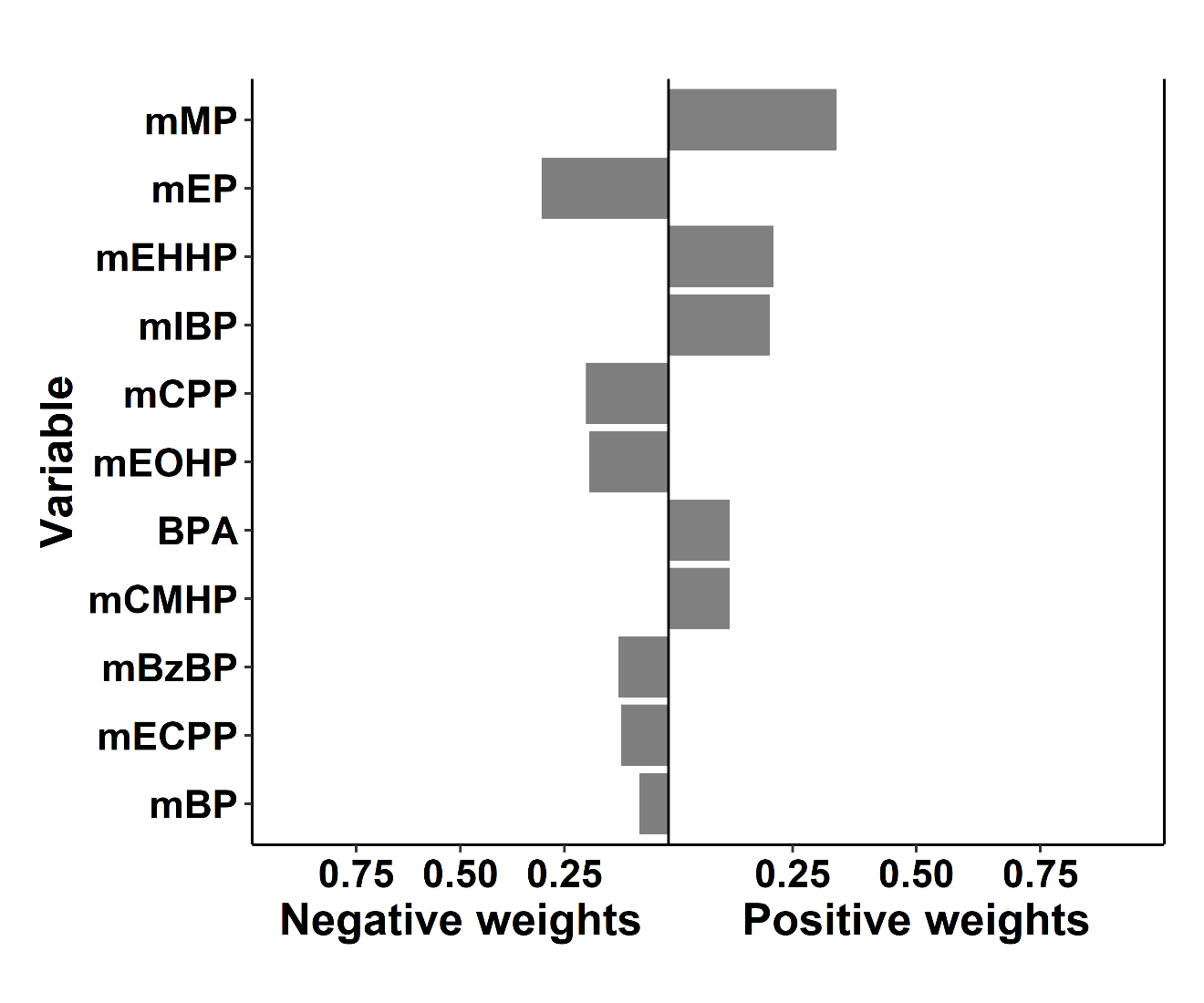


**Figure S8. Weight distribution for the effects of prenatal BPA and phthalate metabolite levels on externalizing problem scores at age 14 years.**

*Figure note:* The distribution of weights for each metabolite in the prenatal exposure mixture is depicted, reflecting the proportion of the overall positive or negative effect attributed to each individual metabolite on externalizing problem scores. Positive weights indicate metabolites contributing to the positive direction of the overall effect, while negative weights reflect metabolites contributing to the negative direction. Interpretation is limited to metabolites contributing to the same direction as the joint effect (positive or negative). Pregnancy chemical concentrations were creatinine adjusted, log 10 transformed, and averaged, prior to including them in the models. Externalizing problem scores were standardized with calculating Z-scores.

Models were adjusted for maternal age, pre-pregnancy body mass index, parity, country of origin, maternal educational levels, marital status, maternal smoking and alcohol drinking habits, as well as gestational age at the time of chemical measurements, child sex (only in models with all children), child age at outcome measurement, and urinary concentrations of organophosphate pesticides during pregnancy.


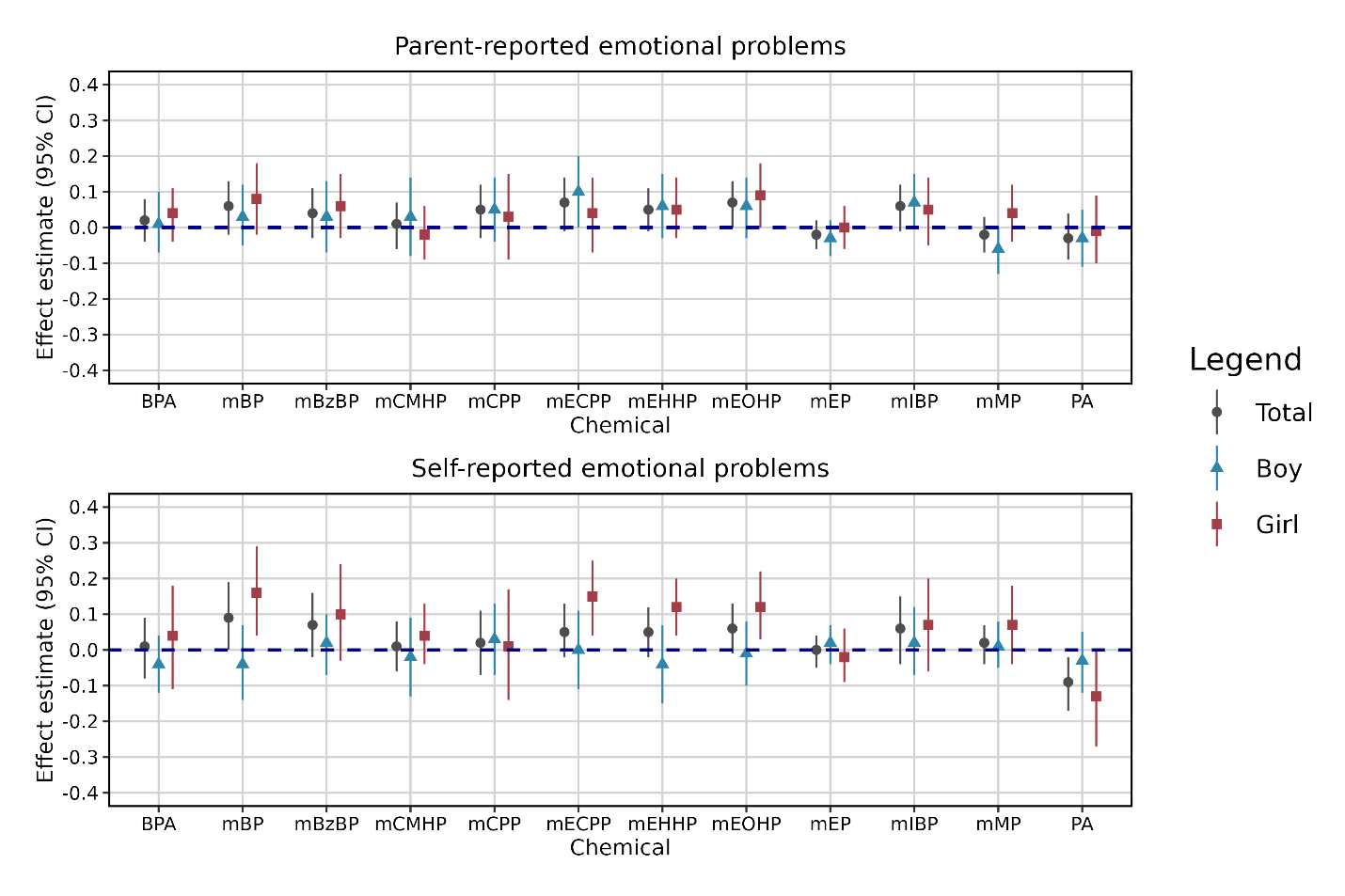


**Figure S9. Associations of prenatal BPA and phthalate exposure with internalizing problem score through age 14 years, without DAP molar sum adjustment**

*Figure note:* Models were adjusted for maternal age, pre-pregnancy body mass index, parity, country of origin, maternal educational levels, marital status, maternal smoking and alcohol drinking habits, as well as gestational age at the time of chemical measurements, child sex (only in models with all children) and child age at outcome measurement, but not for urinary concentrations of organophosphate pesticides during pregnancy.

Effect estimates are reported per log-2 unit increase in creatinine adjusted concentrations of BPA and phthalate metabolites averaged across three measurements in pregnancy. For parent-reports, internalizing problem scores were obtained at ages 3, 6, 10, and 14 years, and for child self-reports internalizing problem scores were obtained at ages 10 and 14 years. All internalizing problem scores were standardized. Vertical lines represent 95% Confidence Intervals.


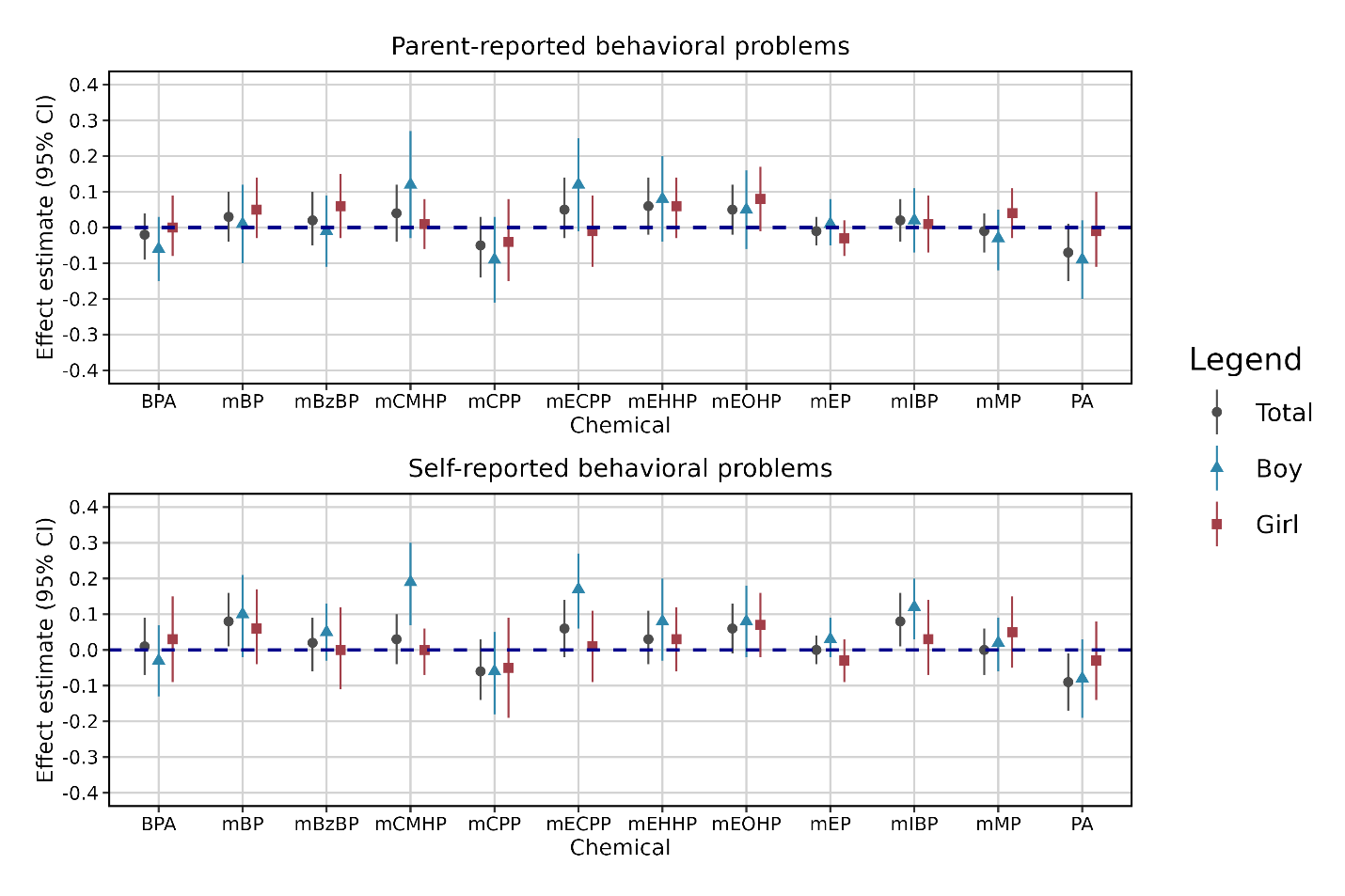


**Figure S10. Associations of prenatal BPA and phthalate exposure with externalizing problem score through age 14 years, without DAP molar sum adjustment**

*Figure note:* Models were adjusted for maternal age, pre-pregnancy body mass index, parity, country of origin, maternal educational levels, marital status, maternal smoking and alcohol drinking habits, as well as gestational age at the time of chemical measurements, child sex (only in models with all children) and child age at outcome measurement, but not for urinary concentrations of organophosphate pesticides during pregnancy.

Effect estimates are reported per log-2 unit increase in creatinine adjusted concentrations of BPA and phthalate metabolites averaged across three measurements in pregnancy. For parent-reports, externalizing problem scores were obtained at ages 3, 6, 10, and 14 years, and for child self-reports externalizing problem scores were obtained at ages 10 and 14 years. All externalizing problem scores were standardized. Vertical lines represent 95% Confidence Intervals.


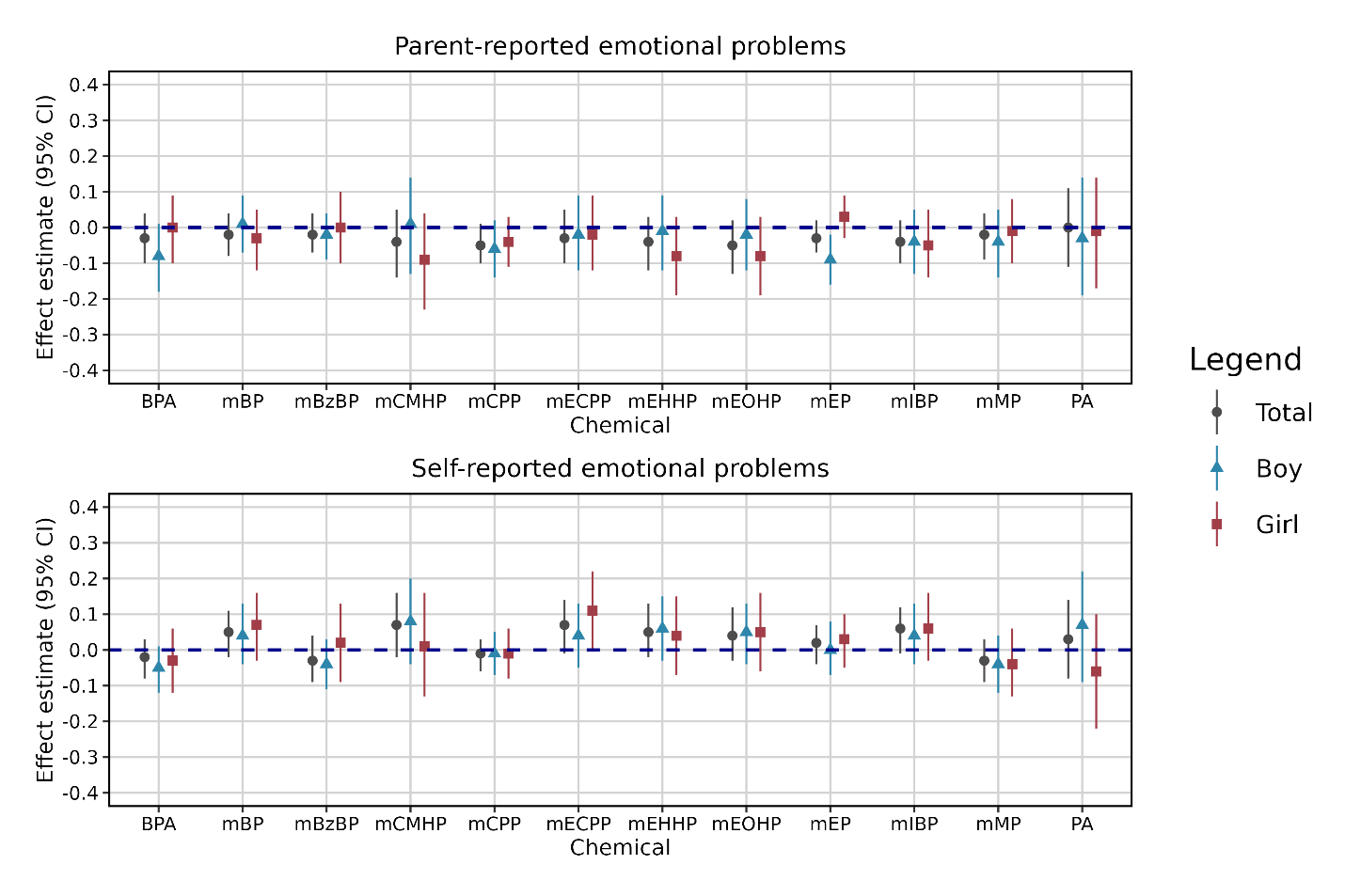


**Figure S11. Associations of childhood BPA and phthalate exposure with internalizing problem score through age 14 years, without DAP molar sum adjustment**

*Figure note:* Models were adjusted for maternal age, pre-pregnancy body mass index, parity, country of origin, maternal educational levels, marital status, maternal smoking and alcohol drinking habits, as well as gestational age at birth, birthweight, child sex (only in models with all children) and child age at outcome measurement, but not for urinary concentrations of childhood organophosphate pesticides.

Effect estimates are reported per log-2 unit increase in creatinine adjusted concentrations of childhood BPA and phthalate metabolites. Internalizing problem scores for both parent-reports and self-reports were obtained at ages 10, and 14 years, and standardized. Vertical lines represent 95% Confidence Intervals.


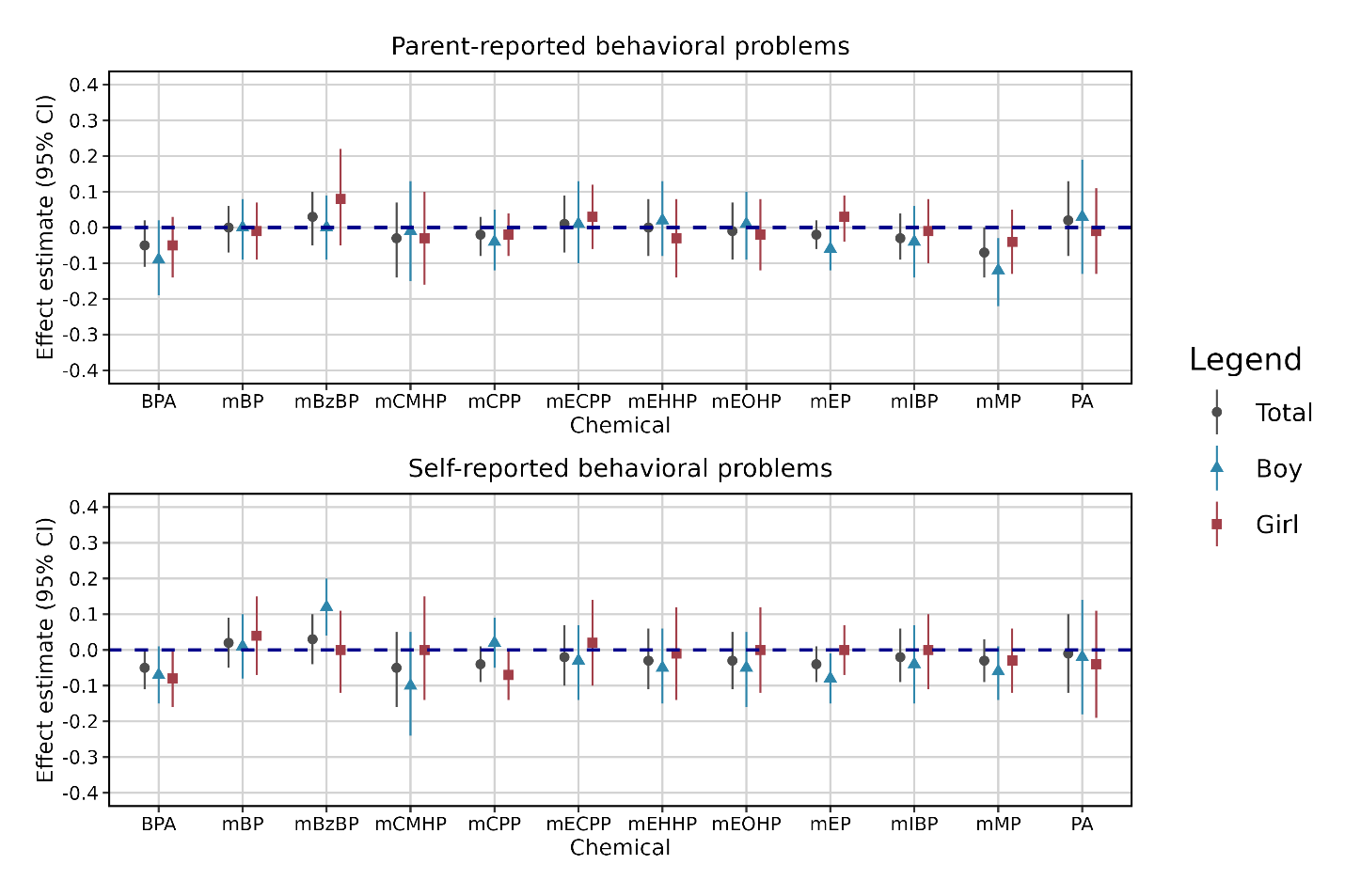


**Figure S12. Associations of childhood BPA and phthalate exposure with externalizing problem score through age 14 years, without DAP molar sum adjustment**

*Figure note:* Models were adjusted for maternal age, pre-pregnancy body mass index, parity, country of origin, maternal educational levels, marital status, maternal smoking and alcohol drinking habits, as well as gestational age at birth, birthweight, child sex (only in models with all children) and child age at outcome measurement, but not for urinary concentrations of childhood organophosphate pesticides.

Effect estimates are reported per log-2 unit increase in creatinine adjusted concentrations of childhood BPA and phthalate metabolites. Externalizing problem scores for both parent-reports and self-reports were obtained at ages 10, and 14 years, and standardized. Vertical lines represent 95% Confidence Intervals.


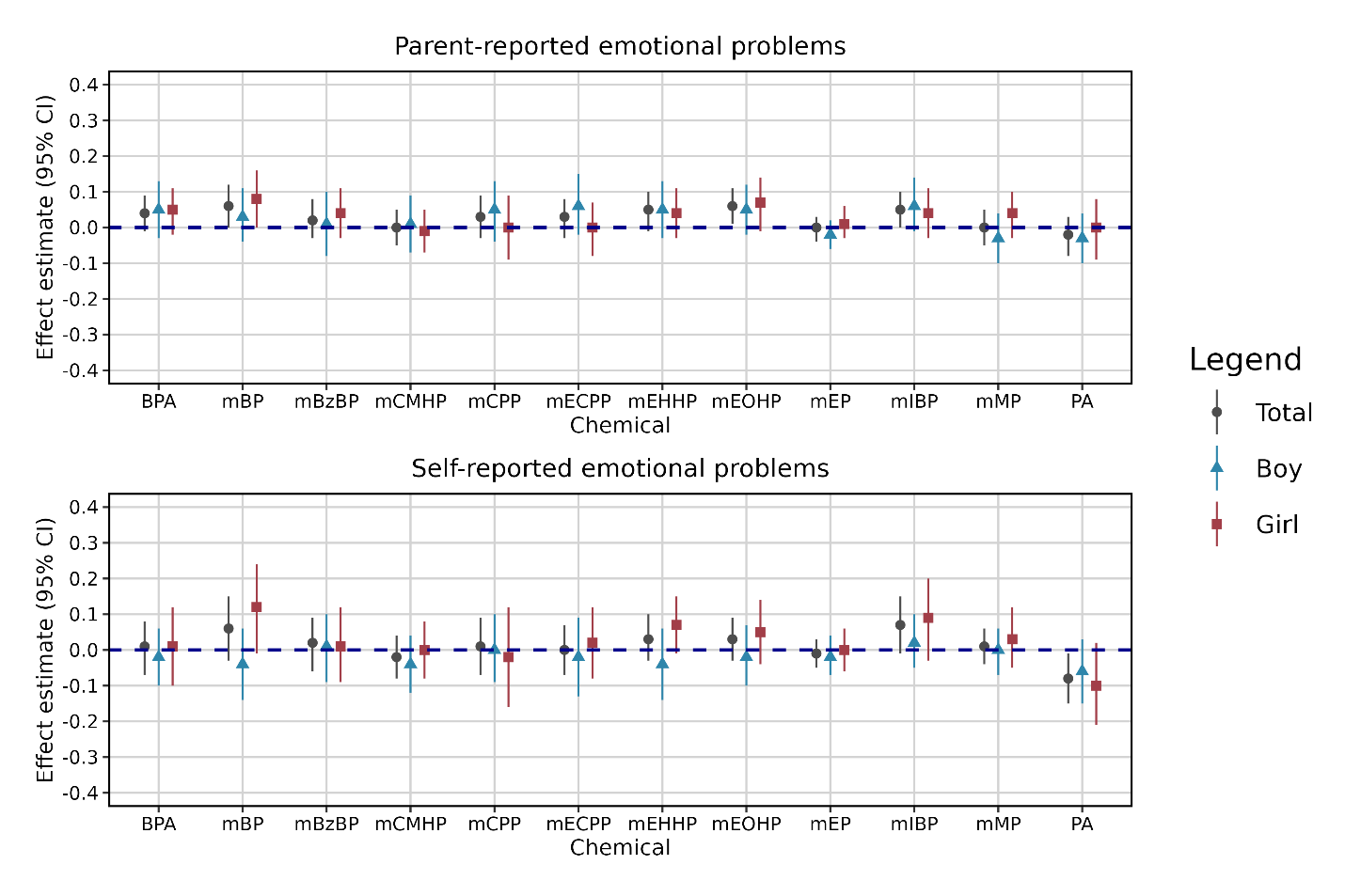


**Figure S13. Associations of prenatal BPA and phthalate exposure with internalizing problem score through age 14 years, excluding childhood BPA and PA adjustment**

*Figure note:* Models were adjusted for maternal age, pre-pregnancy body mass index, parity, country of origin, maternal educational levels, marital status, maternal smoking and alcohol drinking habits, as well as gestational age at the time of chemical measurements, child sex (only in models with all children) and child age at outcome measurement, but not for urinary concentrations of childhood BPA and PA levels.

Effect estimates are reported per log-2 unit increase in creatinine adjusted concentrations of BPA and phthalate metabolites averaged across three measurements in pregnancy. For parent-reports, internalizing problem scores were obtained at ages 3, 6, 10, and 14 years, and for child self-reports internalizing problem scores were obtained at ages 10 and 14 years. All internalizing problem scores were standardized. Vertical lines represent 95% Confidence Intervals.


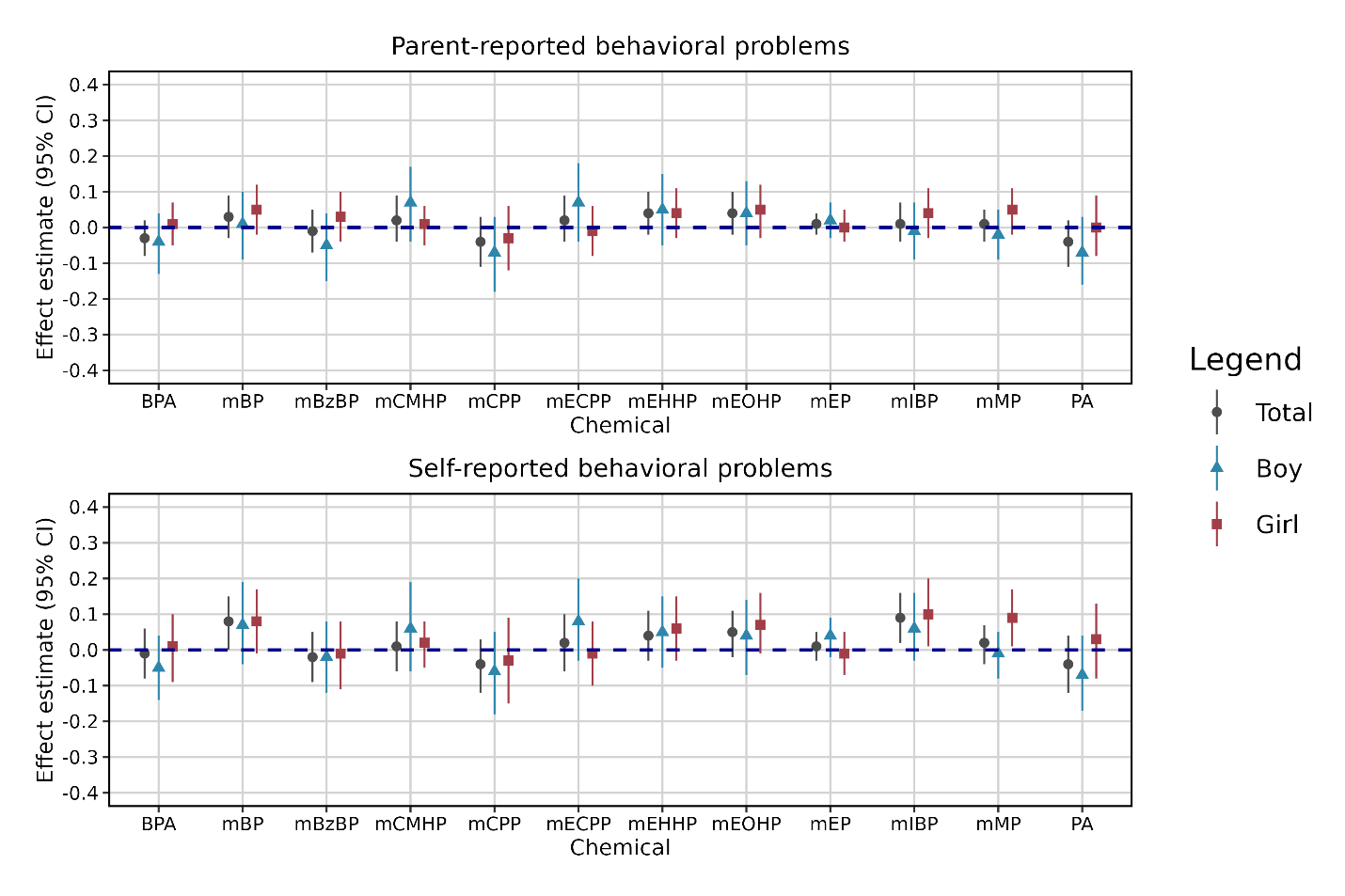


**Figure S14. Associations of prenatal BPA and phthalate exposure with externalizing problem score through age 14 years, excluding childhood BPA and PA adjustment**

*Figure note:* Models were adjusted for maternal age, pre-pregnancy body mass index, parity, country of origin, maternal educational levels, marital status, maternal smoking and alcohol drinking habits, as well as gestational age at the time of chemical measurements, child sex (only in models with all children) and child age at outcome measurement, but not for urinary concentrations of childhood BPA and PA levels.

Effect estimates are reported per log-2 unit increase in creatinine adjusted concentrations of BPA and phthalate metabolites averaged across three measurements in pregnancy. For parent-reports, externalizing problem scores were obtained at ages 3, 6, 10, and 14 years, and for child self-reports externalizing problem scores were obtained at ages 10 and 14 years. All externalizing problem scores were standardized. Vertical lines represent 95% Confidence Intervals.


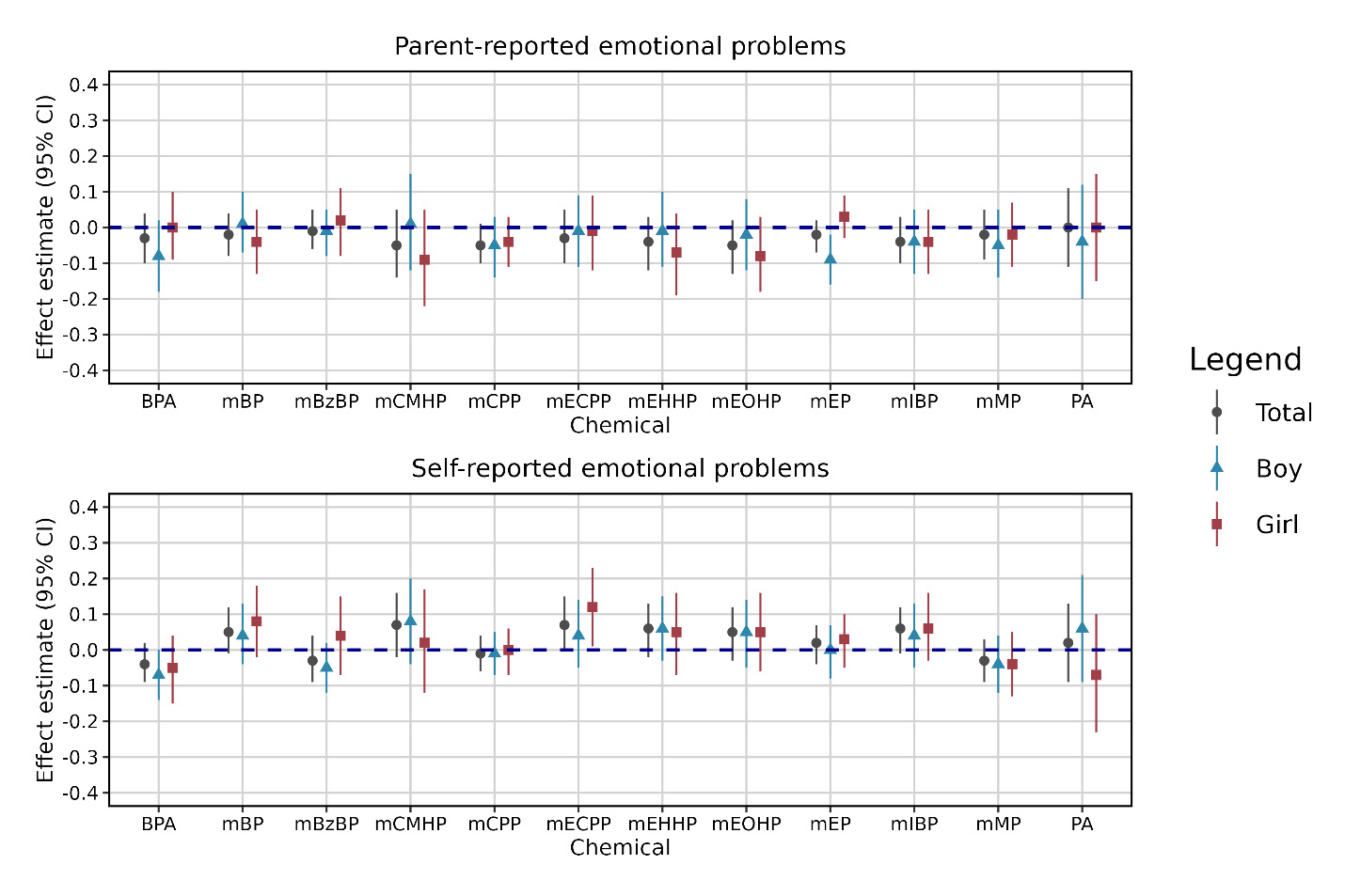


**Figure S15. Associations of childhood BPA and phthalate exposure with internalizing problem score through age 14 years, excluding prenatal BPA and PA adjustment**

*Figure note:* Models were adjusted for maternal age, pre-pregnancy body mass index, parity, country of origin, maternal educational levels, marital status, maternal smoking and alcohol drinking habits, as well as gestational age at birth, birthweight, child sex (only in models with all children) and child age at outcome measurement, but not for urinary concentrations of prenatal BPA and PA levels.

Effect estimates are reported per log-2 unit increase in creatinine adjusted concentrations of childhood BPA and phthalate metabolites. Internalizing problem scores for both parent-reports and self-reports were obtained at ages 10, and 14 years, and standardized. Vertical lines represent 95% Confidence Intervals.


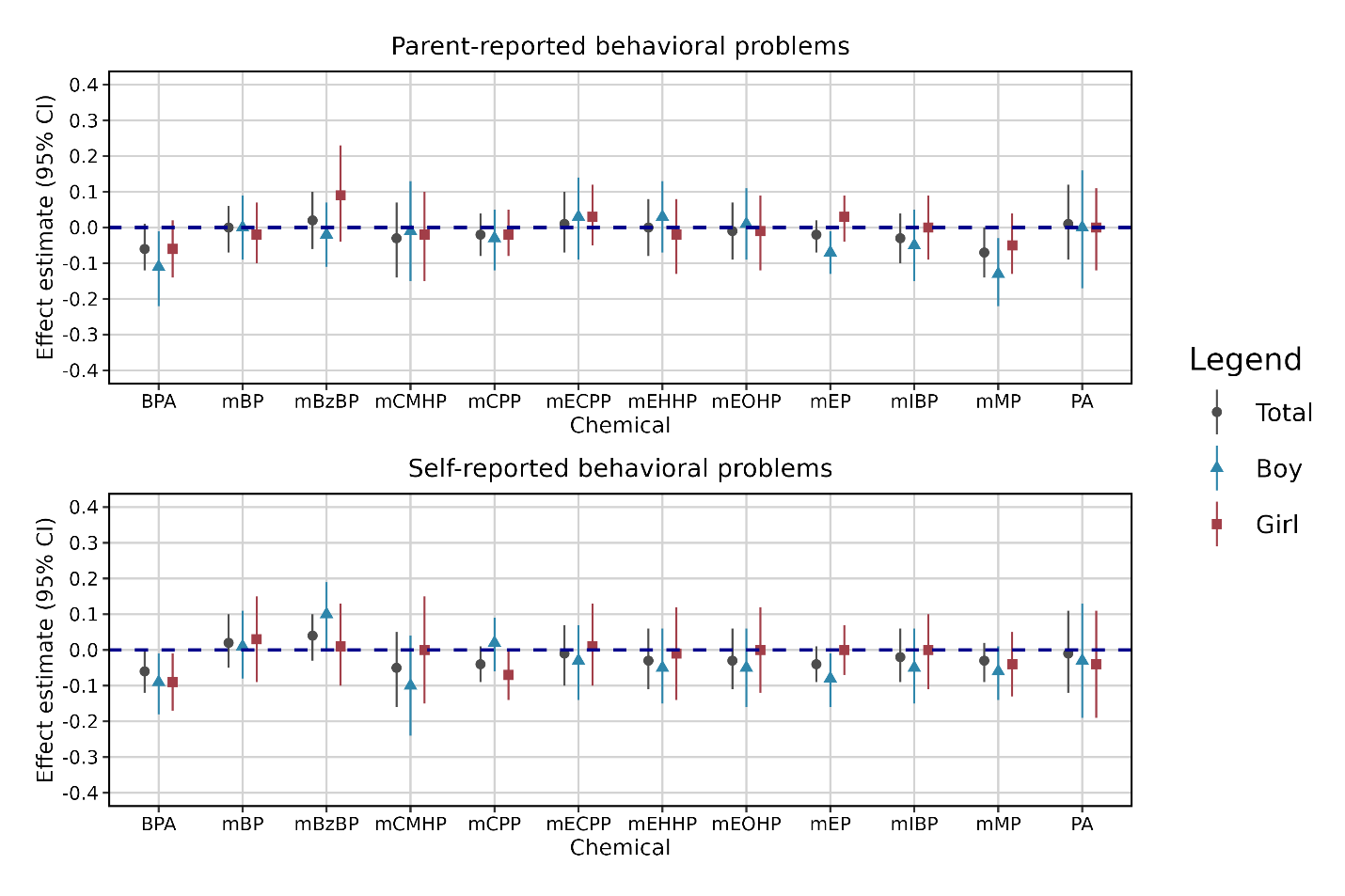


**Figure S16. Associations of childhood BPA and phthalate exposure with externalizing problem score through age 14 years excluding prenatal BPA and PA adjustment**

*Figure note:* Models were adjusted for maternal age, pre-pregnancy body mass index, parity, country of origin, maternal educational levels, marital status, maternal smoking and alcohol drinking habits, as well as gestational age at birth, birthweight, child sex (only in models with all children) and child age at outcome measurement, but not for urinary concentrations of prenatal BPA and PA levels.

Effect estimates are reported per log-2 unit increase in creatinine adjusted concentrations of childhood BPA and phthalate metabolites. Externalizing problem scores for both parent-reports and self-reports were obtained at ages 10, and 14 years, and standardized. Vertical lines represent 95% Confidence Intervals.


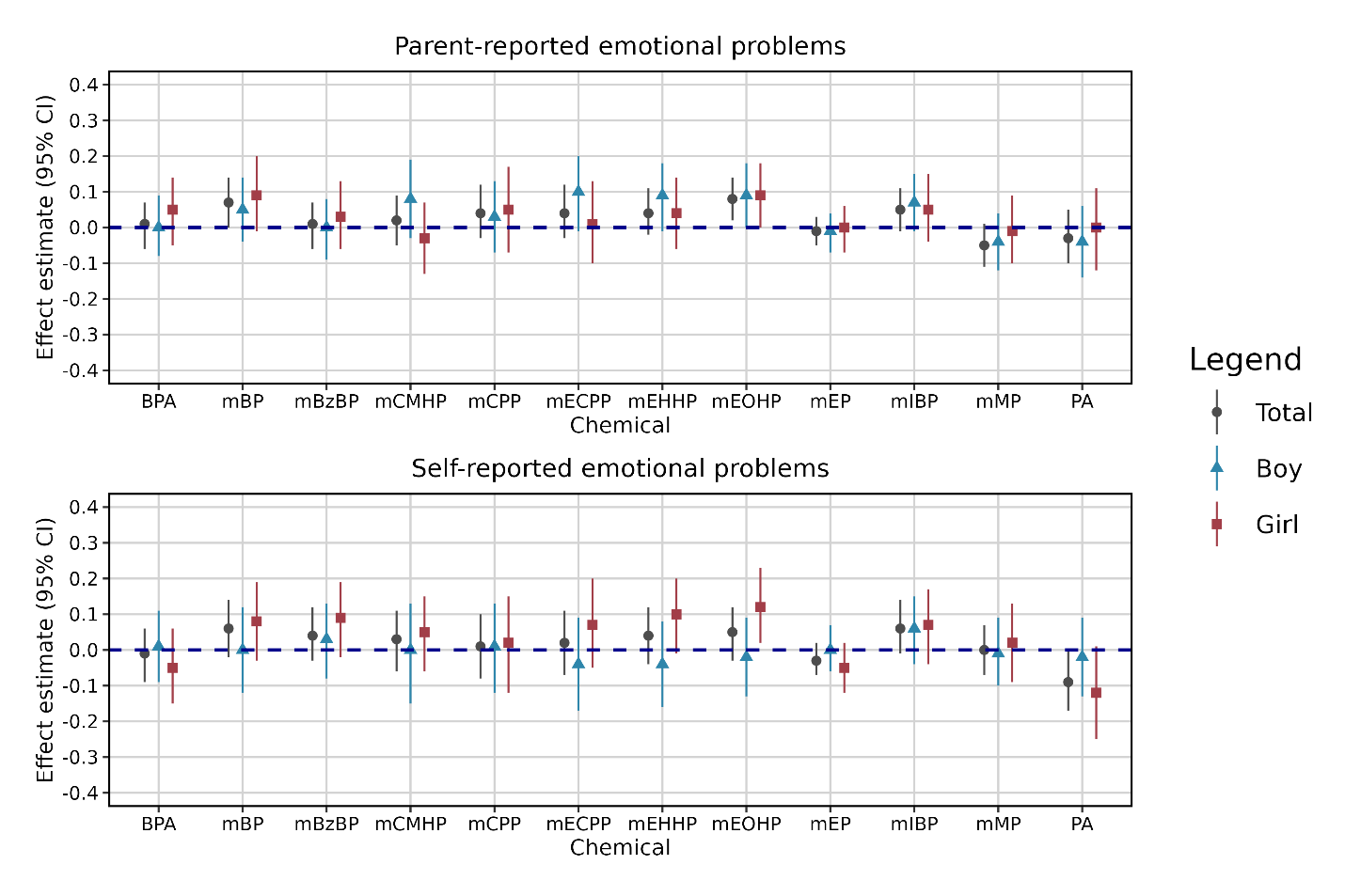


**Figure S17. Associations of prenatal BPA and phthalate exposure with internalizing problem score through age 14 years in complete case data**

*Figure note:* Models were adjusted for maternal age, pre-pregnancy body mass index, parity, country of origin, maternal educational levels, marital status, maternal smoking and alcohol drinking habits, as well as gestational age at the time of chemical measurements, child sex (only in models with all children) and child age at outcome measurement, as well as for urinary concentrations of organophosphate pesticides during pregnancy.

Effect estimates are reported per log-2 unit increase in creatinine adjusted concentrations of BPA and phthalate metabolites averaged across three measurements in pregnancy. For parent-reports, internalizing problem scores were obtained at ages 3, 6, 10, and 14 years, and for child self-reports internalizing problem scores were obtained at ages 10 and 14 years. All internalizing problem scores were standardized. Vertical lines represent 95% Confidence Intervals.

**
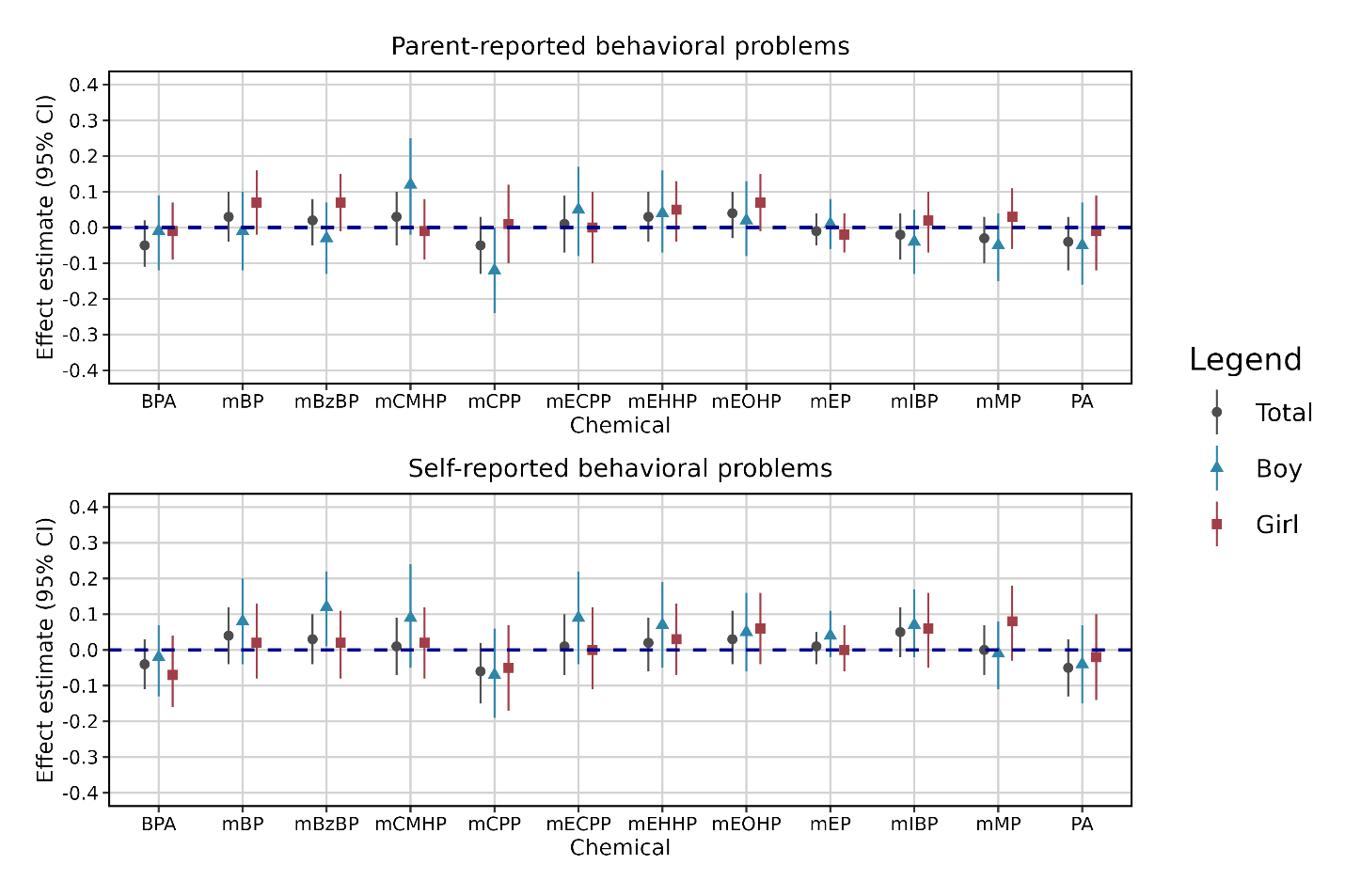
Figure S18. Associations of prenatal BPA and phthalate exposure with externalizing problem score through age 14 years in complete case data**

*Figure note:* Models were adjusted for maternal age, pre-pregnancy body mass index, parity, country of origin, maternal educational levels, marital status, maternal smoking and alcohol drinking habits, as well as gestational age at the time of chemical measurements, child sex (only in models with all children) and child age at outcome measurement, as well as urinary concentrations of organophosphate pesticides during pregnancy.

Effect estimates are reported per log-2 unit increase in creatinine adjusted concentrations of BPA and phthalate metabolites averaged across three measurements in pregnancy. For parent-reports, externalizing problem scores were obtained at ages 3, 6, 10, and 14 years, and for child self-reports externalizing problem scores were obtained at ages 10 and 14 years. All externalizing problem scores were standardized. Vertical lines represent 95% Confidence Intervals.

**
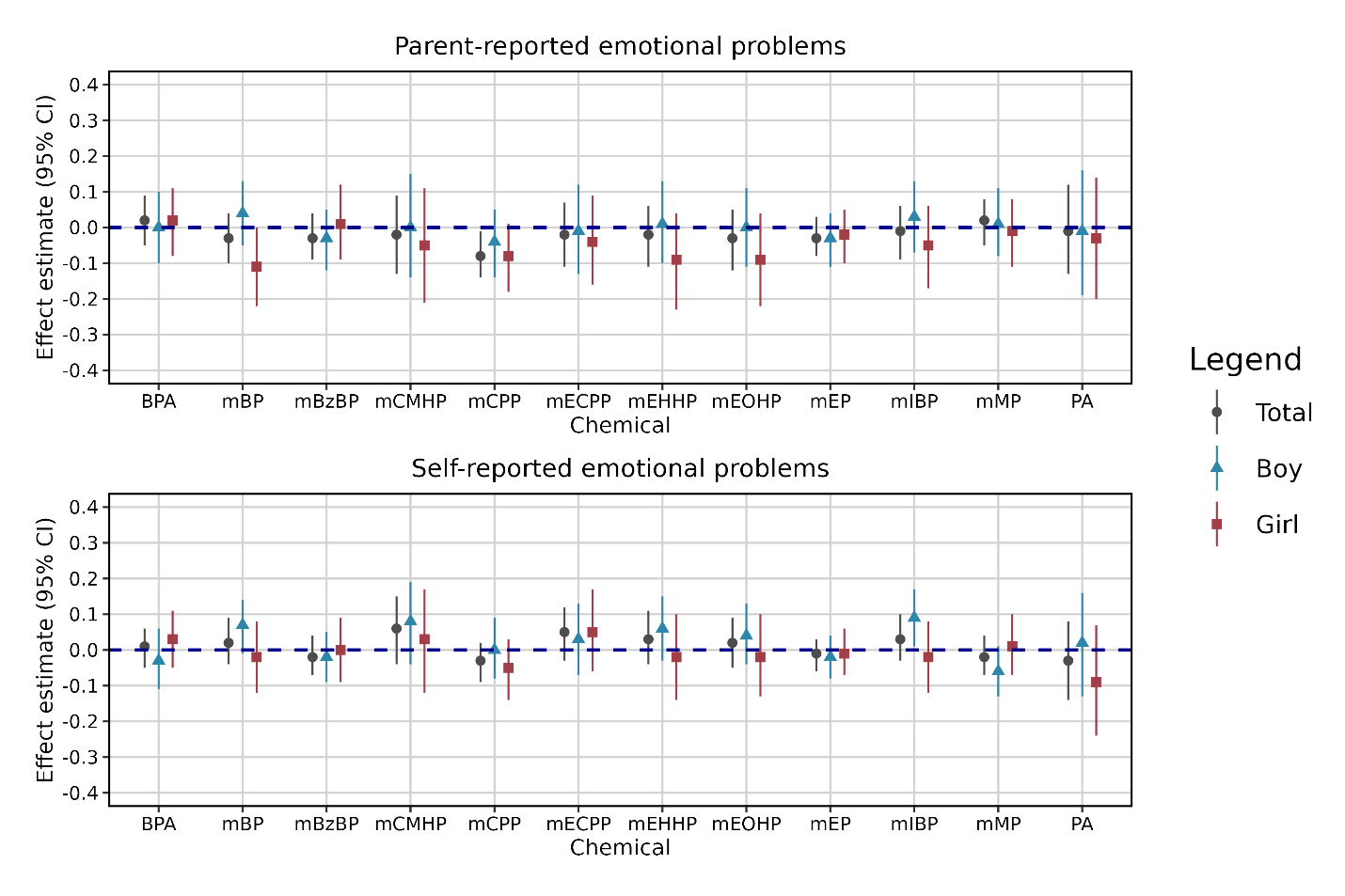
Figure S19. Associations of childhood BPA and phthalate exposure with internalizing problem score through age 14 years in complete case data**

*Figure note:* Models were adjusted for maternal age, pre-pregnancy body mass index, parity, country of origin, maternal educational levels, marital status, maternal smoking and alcohol drinking habits, as well as gestational age at birth, birthweight, child sex (only in models with all children) and child age at outcome measurement, as well as for urinary concentrations of childhood organophosphate pesticides.

Effect estimates are reported per log-2 unit increase in creatinine adjusted concentrations of childhood BPA and phthalate metabolites. Internalizing problem scores for both parent-reports and self-reports were obtained at ages 10, and 14 years, and standardized. Vertical lines represent 95% Confidence Intervals.

**
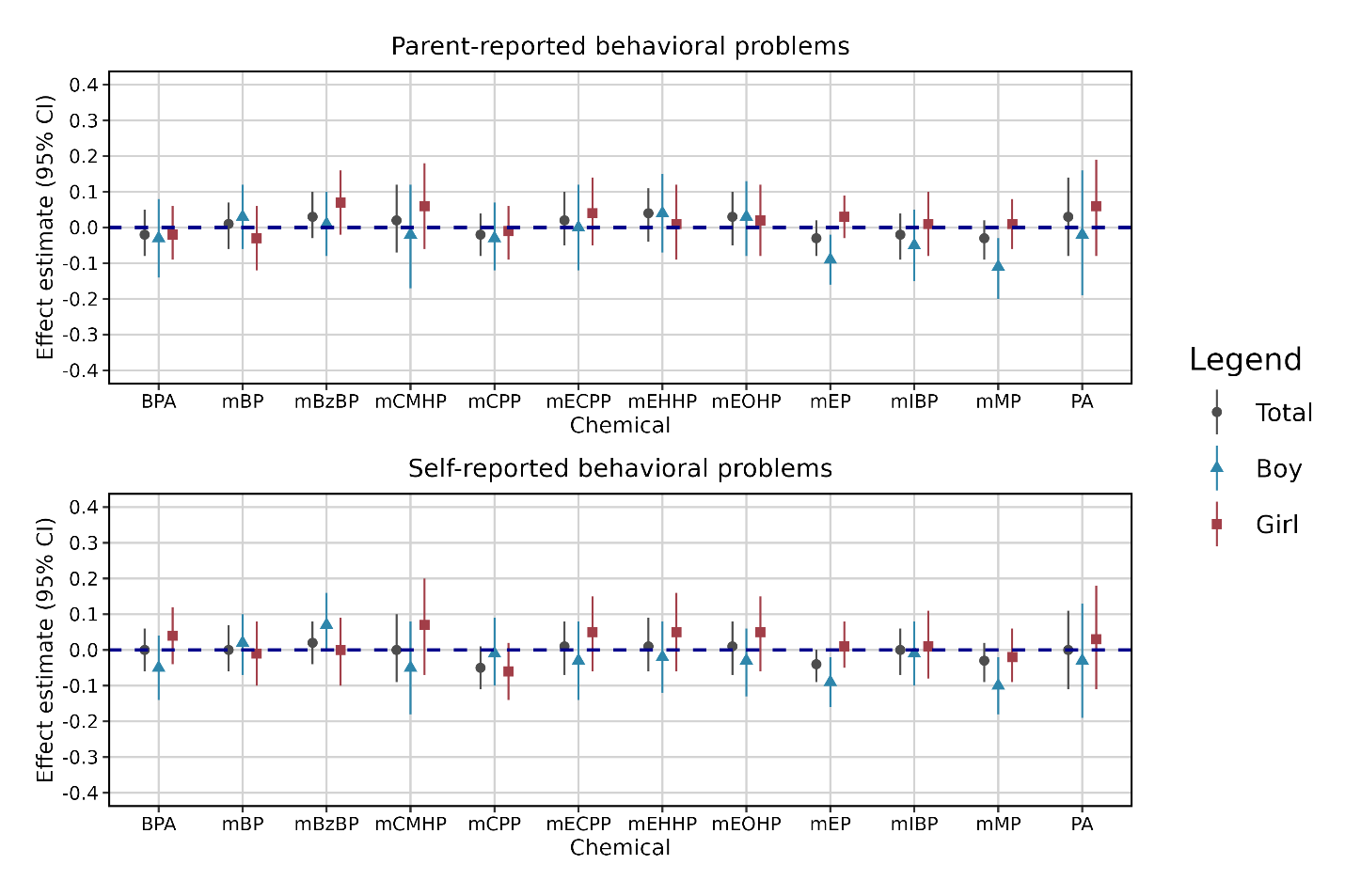
Figure S20. Associations of childhood BPA and phthalate exposure with externalizing problem score through age 14 years in complete case data**

*Figure note:* Models were adjusted for maternal age, pre-pregnancy body mass index, parity, country of origin, maternal educational levels, marital status, maternal smoking and alcohol drinking habits, as well as gestational age at birth, birthweight, child sex (only in models with all children) and child age at outcome measurement, as well as for urinary concentrations of childhood organophosphate pesticides.

Effect estimates are reported per log-2 unit increase in creatinine adjusted concentrations of childhood BPA and phthalate metabolites. Externalizing problem scores for both parent-reports and self-reports were obtained at ages 10, and 14 years, and standardized. Vertical lines represent 95% Confidence Intervals.
